# Supplementary material for: A checkpoint function for Nup98 in nuclear pore formation suggested by novel inhibitory nanobodies
Source: EMBO J. 2024 Apr 22;43(11):6. doi: 10.1038/s44318-024-00081-w (PMC11148069; doi:10.1038/s44318-024-00081-w)
Supplement: Supplementary file 1 — Appendix [file 44318_2024_81_MOESM1_ESM.pdf]

Appendix

A checkpoint function for Nup98 in nuclear pore formation suggested by inhibitory nanobodies

Mireia Solà Colom[1,2], Zhenglin Fu[1], Philip Gunkel[1], Thomas Güttler[1,3], Sergei Trakhanov[1], Vasundara Srinivasan[1,4], Kathrin Gregor[1], Tino Pleiner[1,5], & Dirk Görlich[1*]

[1] Department of Cellular Logistics, Max Planck Institute for Multidisciplinary Sciences, Göttingen, Germany; [2] present address: AI Proteins, 20 Overland St., MA Boston, Massachusetts, USA; [3] present address: Octapharma Biopharmaceuticals, Im Neuenheimer Feld 590, 69120 Heidelberg; [4] present address: Department of Chemistry, Institute of Biochemistry and Molecular Biology, Universität Hamburg; [5] present address: Department of Molecular and Cellular Physiology, Stanford University School of Medicine, Stanford, CA, USA.

* Correspondence: goerlich@mpinat.mpg.de

Contents:

Appendix Figures S1-S5

Appendix Tables S1-S5

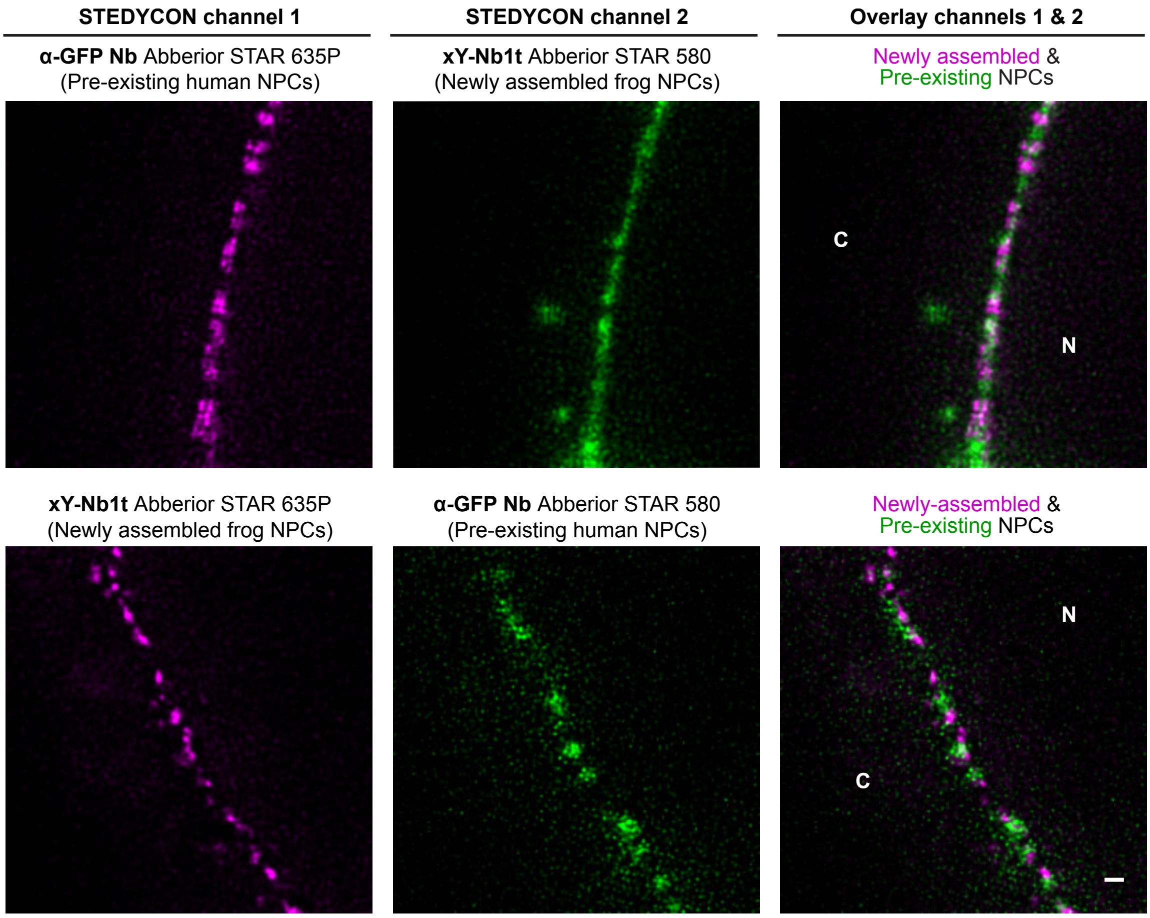

Appendix Figure S1. *In vitro* insertion of new *Xenopus* NPCs into human NEs in interphase mode. Experiment is identical to Fig. 5A-B, but STED images show the equatorial planes of the nuclei. Scale bar, 200 nm.

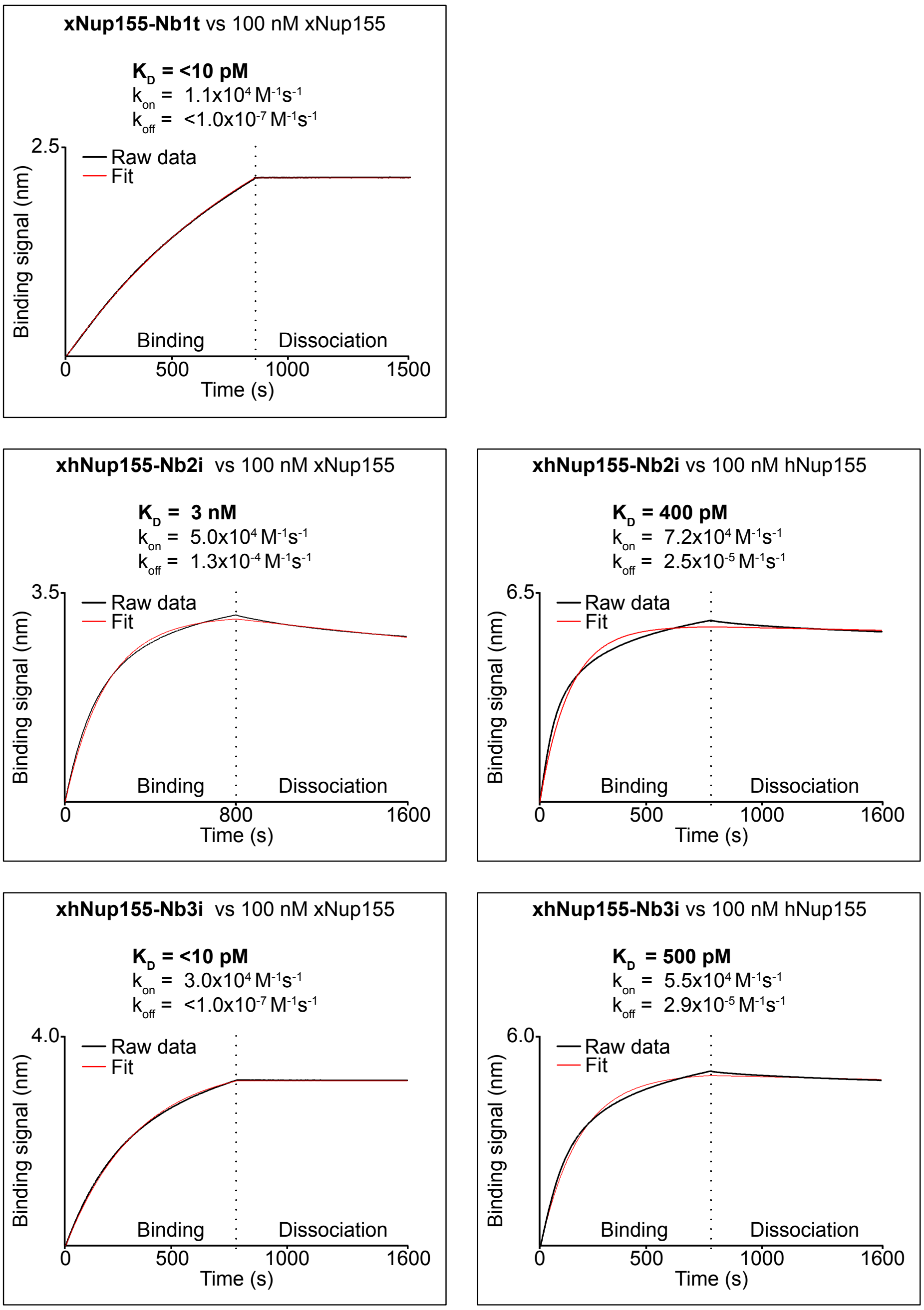

Appendix Figure S2A. Affinity estimations for anti-Nup155 nanobodies. Indicated biotinylated nanobodies were bound to a binding signal of 1 nm on High Precision Streptavidin sensor chips of an Octet RED96e instrument. After a wash step, the nanobodies were allowed to bind full-length *Xenopus* (left) or human Nup155 as an analyte. The binding and subsequent dissociation steps were each for 800 seconds. The analyte concentration (here 100 nM) is indicated. Graphs show the corresponding biolayer interferometry (BLI) traces. On-rates (k_{on}), off-rates (k_{off}), and dissociation constants (K_Ds) were estimated by data fitting using the Octet Analysis HT 12.0 software.

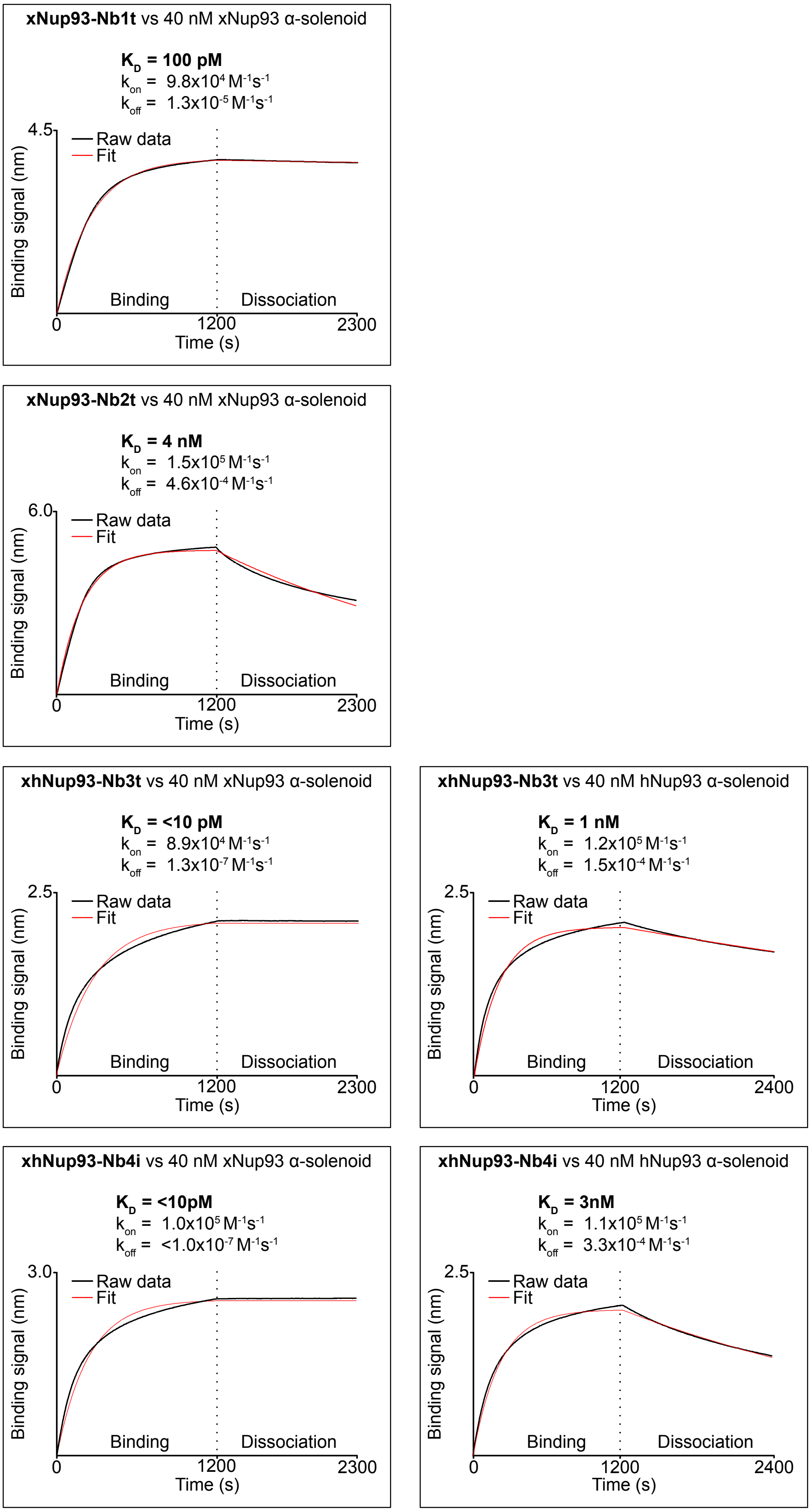

Appendix Figure S2B. BLI of anti-Nup93 nanobodies. Measurements as in Fig. S2A but with indicated nanobodies and either the *Xenopus* or the human Nup93 α-solenoid domain as an analyte.

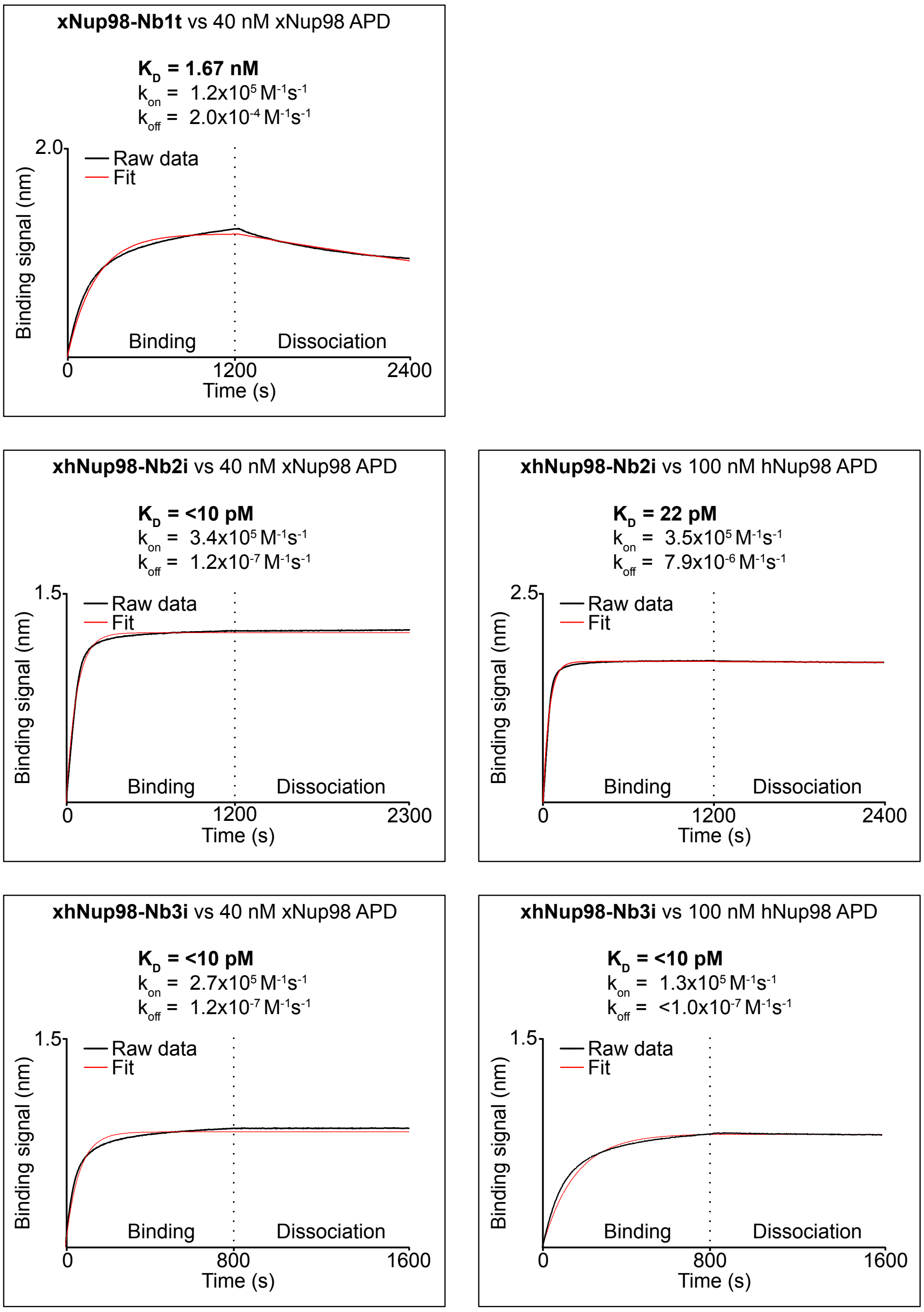

Appendix Figure S2B. BLI of anti-Nup98 nanobodies. Measurements as in Fig. S2A but with indicated nanobodies and either the *Xenopus* Nup98 APD or the human Nup98 APD as an analyte.

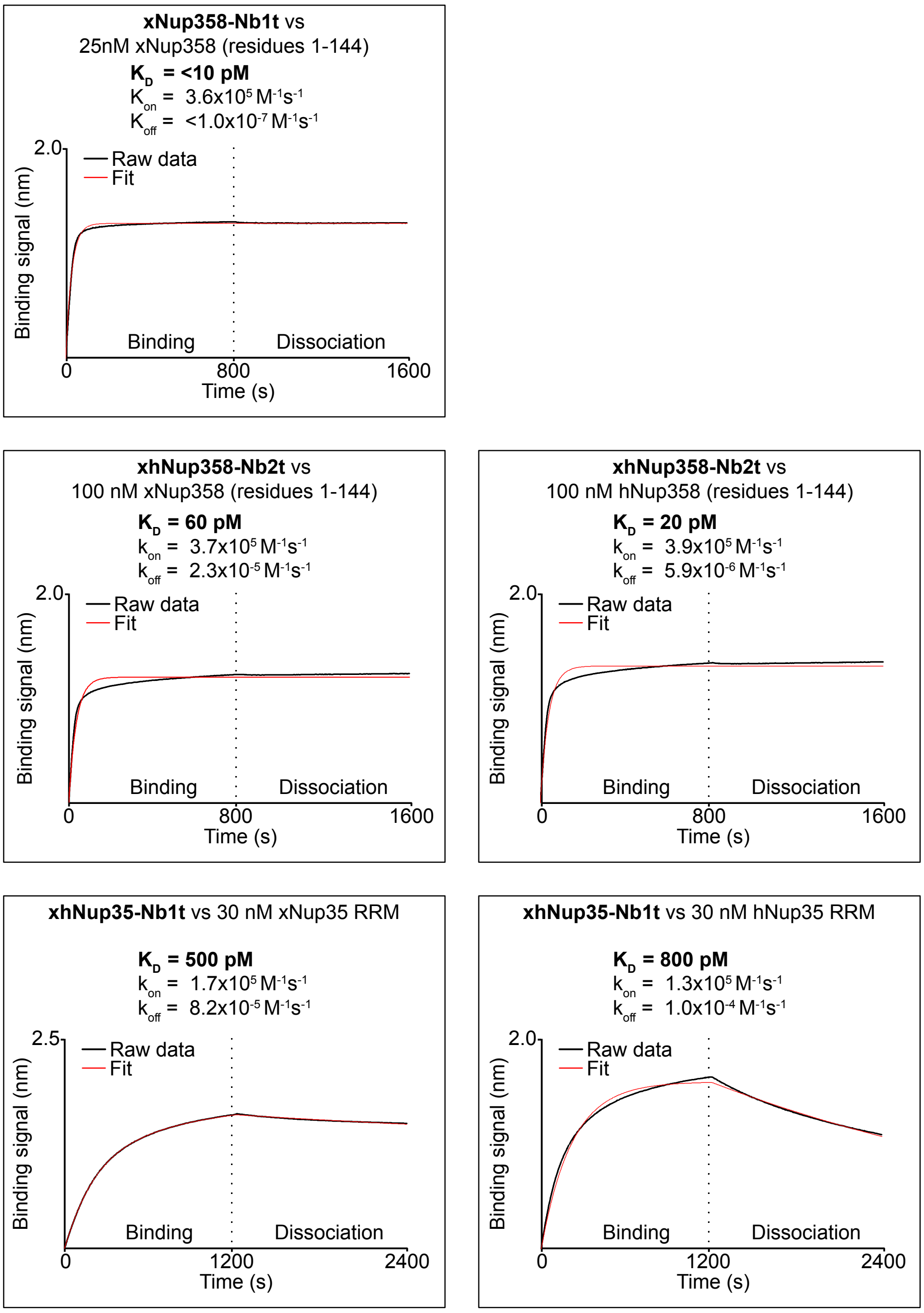

Appendix Figure S2D. Affinity measurements of anti-Nup358 and anti-Nup35 nanobodies. Measurements as in Fig. S2A but with indicated nanobodies and analytes.

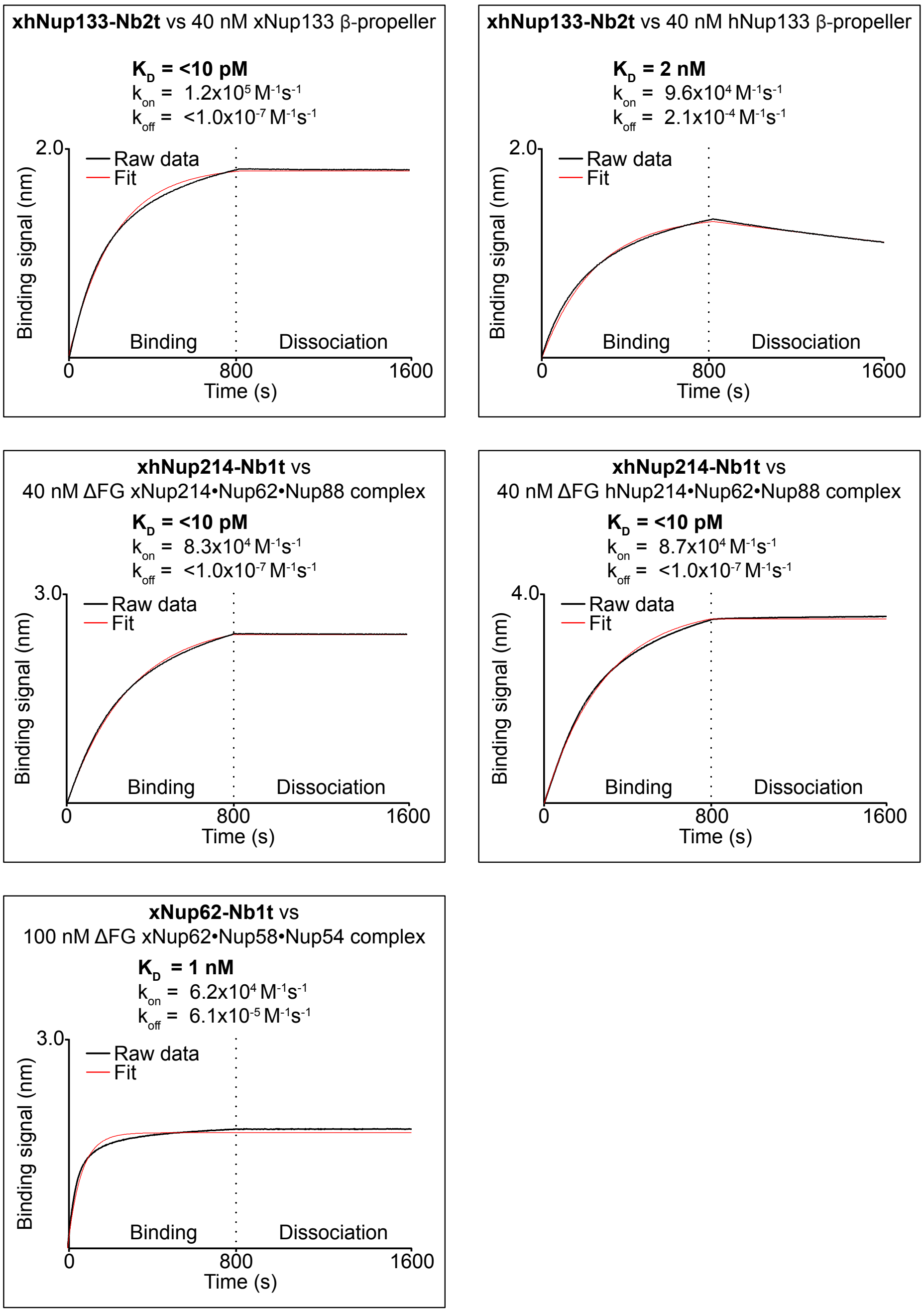

Appendix Figure S2E. Affinity measurements of nanobodies directed against Nup133, and the Nup214·Nup88·Nup62 and Nup62·Nup58·Nup54 complexes. Measurements as in Fig. S2A but with indicated nanobodies and analytes.

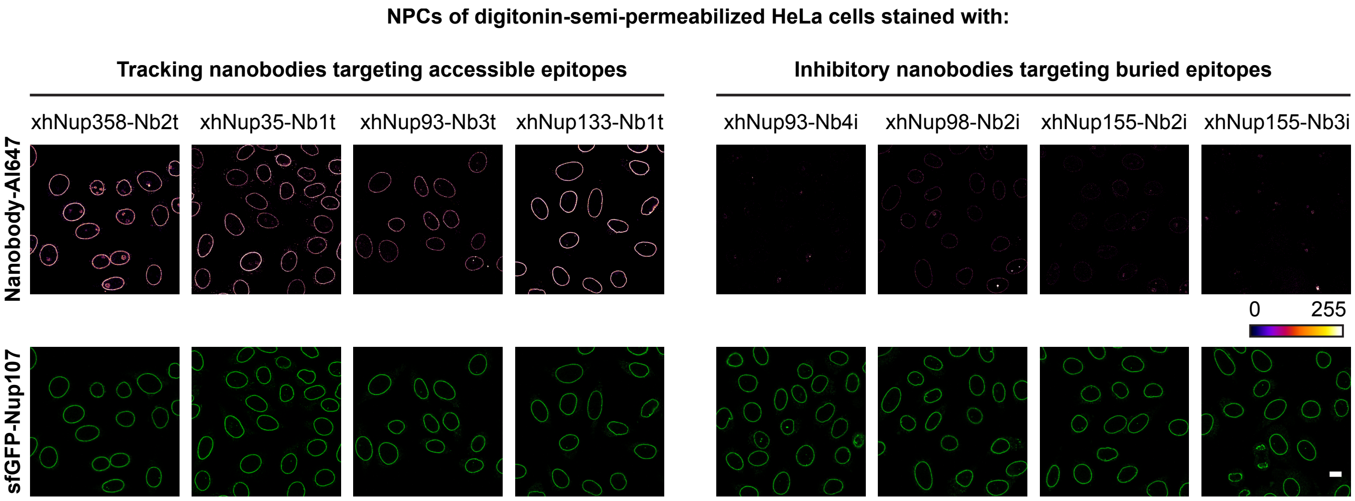

Appendix Figure S3. Tracking but not inhibitory nanobodies produce bright NPC signals in unfixed, semi-permeabilized HeLa cells. Same experiment as in Fig. 6A but analyzing unfixed HeLa cells permeabilized with 30 µg/ml digitonin and thus with intact nuclear membranes. Staining was with 30 nM of indicated tracking or inhibitory nanobodies. Cells were imaged live using a Leica SP8 confocal microscope and identical settings. Scale bar, 10 µm.

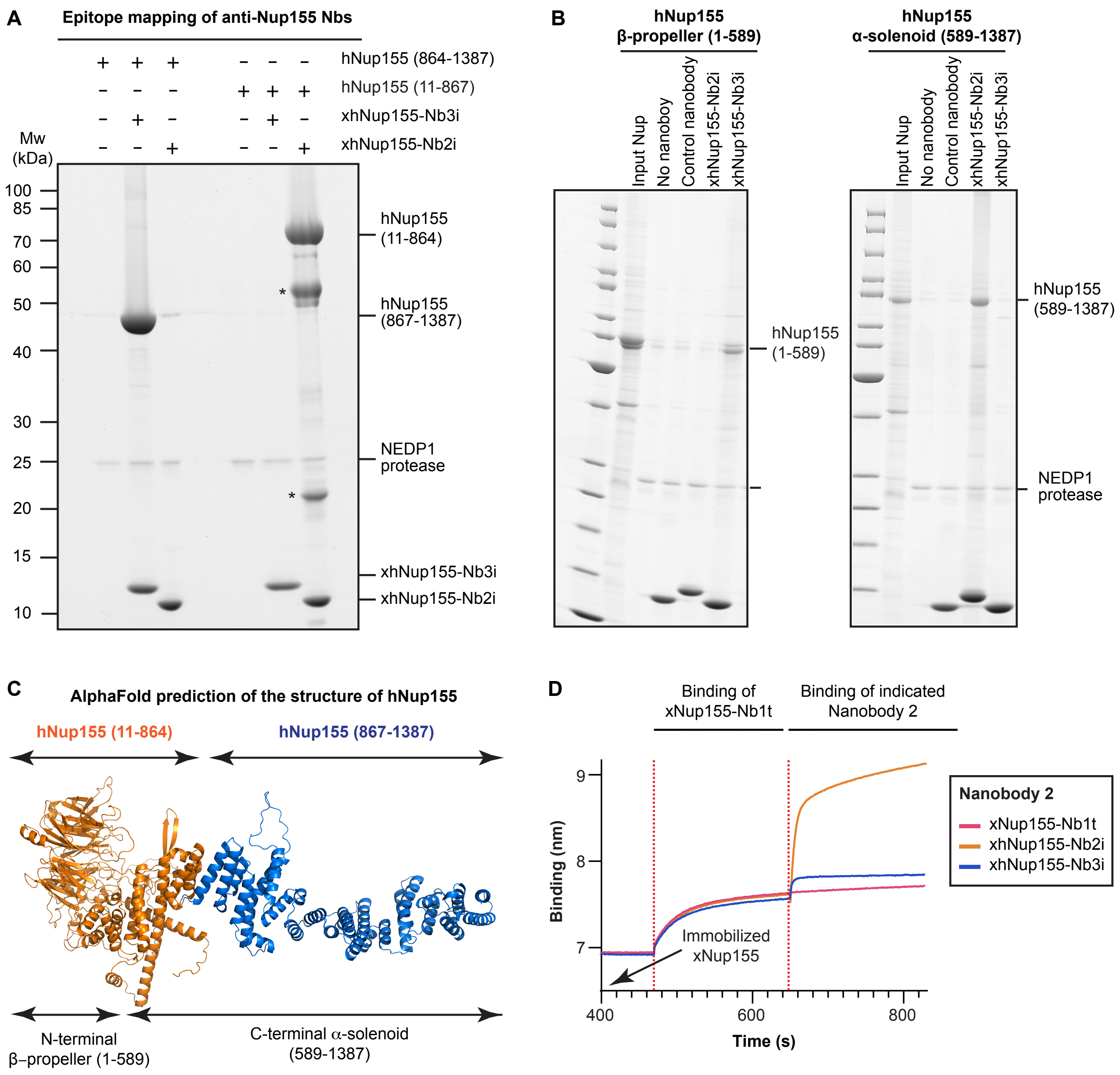

Appendix Figure S4. Epitope mapping of the anti-Nup155 nanobodies used in this study. (A, B) xhNup155-Nb2i binds to the N-terminal β-propeller domain of Nup155, whereas xhNup155-Nb3i binds to its C-terminal α-solenoid. His_{14}-NEDD8-nanobody fusions were immobilized onto a Ni^{2+} chelate matrix and incubated with the indicated Nup155 fragments. Nanobodies with bound targets were then eluted by the NEDP1 protease (Frey and Görlich, 2014) and analyzed by SDS-PAGE/Coomassie-staining. (*) Cleavage products of the N-terminal Nup155 fragment that occurred spontaneously during expression/purification from *E. coli*. **(C)** AlphaFold prediction of human Nup155, colored to indicate the two fragments analyzed in (A). **(D)** Epitope binning experiments by bio-layer interferometry (BLI) confirmed that xhNup155-Nb2i and xhNup155-Nb3i are each orthogonal to xNup155-Nb1t. Biotinylated full-length xNup155 was loaded onto streptavidin sensor chips. The loaded sensors were then dipped into wells containing saturating amounts of the xNup155-Nb1t and subsequently into wells containing one of the three different nanobodies indicated.

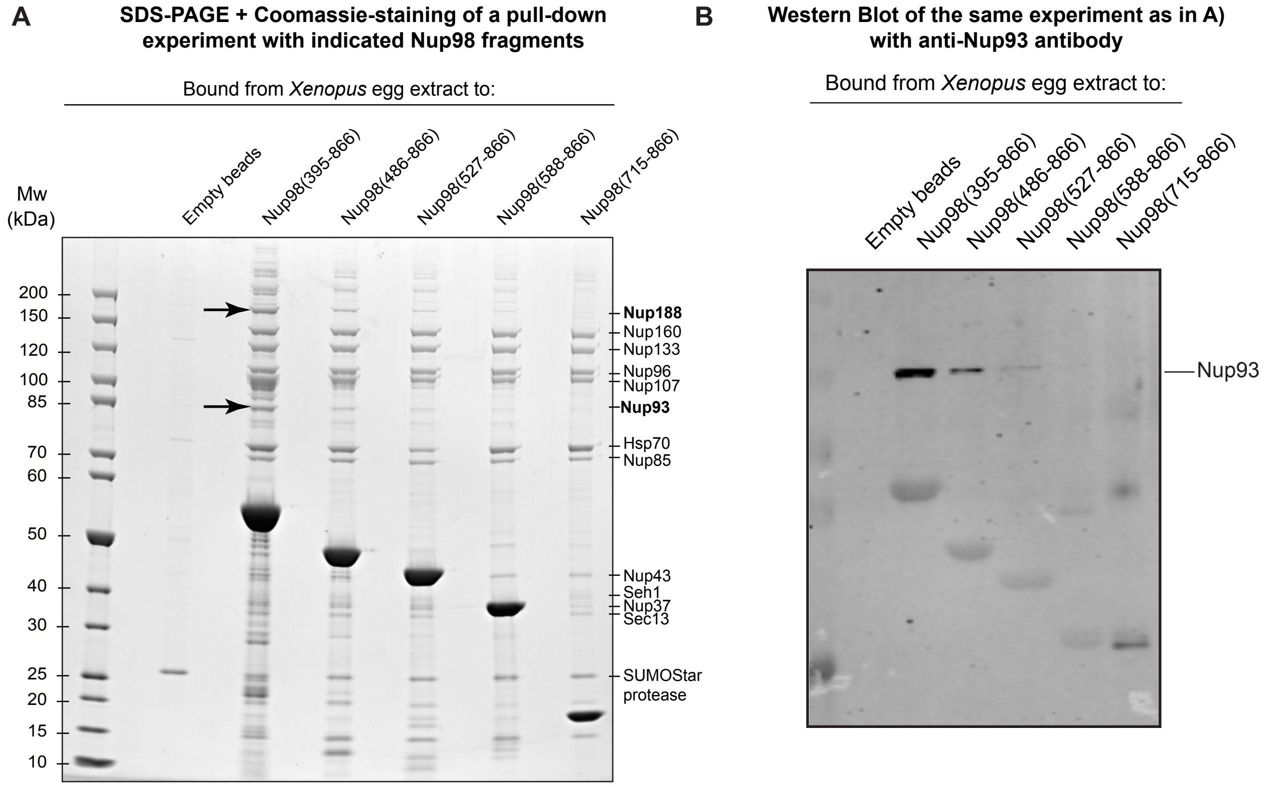

Appendix Figure S5. Nup98 fragments that interact with the Nup188·Nup93 complex from *Xenopus* egg extract. (A) The indicated Nup98 truncations were purified from *E. coli* as H_{14}-Avi-biotin-SumoSTAR fusions, immobilized on streptavidin-agarose and incubated with the soluble fraction from *Xenopus* egg extract. After washing, formed Nup98·Nup complexes were eluted by SumoSTAR protease (Peroutka et al., 2008) and analyzed by SDS-PAGE/Coomassie-staining. Identity of protein bands was confirmed by mass spectrometry. Bands corresponding to Nup93 and Nup188 are indicated with arrows. **(B)** The binding assay was performed as described in (A), but analysis was an immunoblot using an anti-xNup93 antibody.

Appendix Table S1. X-ray data collection and refinement statistics for the homodimeric Nup35 RRM domain with bound xhNup35-Nb1t tracking nanobody

**Structure**	**hNup35 RRM·xhNup35-Nb1t**
**PDB data entry**	8OZB
**Space group**	P2221
**Unit cell dimensions**<br>a, b, c (Å)<br>α, β, γ (°)	 49.12, 77.22, 127.47<br>90.00, 90.00, 90.00
**Resolution (Å)**	77.02–2.09 (2.15-2.09)[a]
**Wavelength (Å)**	0.9763
**R_{merge}**	0.193 (1.89)
**R_{factor}**	0.2416
**R_{free}**	0.2579
**<I/σ(I)>**	8.1 (1.4)
**$CC_{1/2}$**	
**Reflections**<br>Measured<br>Unique	 363326 (17256)<br>29113 (1993)
**Completeness**	98.6 (89.7)
**Multiplicity**	12.5 (8.7)
**Number of atoms**	3053
**Protein/Solvent**	2983/70
**R.m.s. deviation from ideal**<br>Bond lengths (Å)<br>Bond angles (°)	 0.010<br>1.739
**Ramachandran statistics (%)**<br>Favored<br>Allowed<br>Outliers	 96.6<br>3.2<br>0.0
**<B-factor> (A^2)**	38.23

[a] Values in parentheses are for the highest resolution cell.

Appendix Table S2. X-ray data collection and refinement statistics for the unliganded xhNup93-Nb4i and xNup93-Nb2t nanobodies

Structure	xhNup93-Nb4i	xNup93-Nb2t
**PDB data entry**	8CDS	8CDT
**Space group**	$P12_11$	$P6_3$
**Unit cell dimensions**		
a, b, c (Å)	26.88, 42.11, 44.95	99.97, 99.97, 30.71
α, β, γ (°)	90.00, 93.06, 90.00	90, 90, 120
**Resolution (Å)**	30.71–1.53 (1.59–1.53)	32.72–1.41 (1.46–1.41)
**Reflections**		
Total	91577 (8479)	677703 (64109)
Unique	14726 (1455)	34229 (3378)
**Multiplicity**	6.2 (5.8)	19.8 (19.0)
**Completeness (%)**	96.37 (94.05)	99.94 (99.85)
**Mosaicity (°)**	0.572	0.152
**R_{merge} (%)**	5.4 (40.2)	4.4 (72.9)
**R_{pim} (%)**	2.3 (18.2)	1.0 (17.3)
**Mean I/σI**	15.09 (0.77)	37.91 (3.98)
**CC1/2 (%)**	99.7 (91.4)	100 (97.0)
**R_{work}** (%)	13.63 (17.21)	15.80 (30.89)
**R_{free}** (%)	17.67 (24.28)	18.83 (26.61)
**Number of atoms**		
Protein	1020	961
Ligands	11	12
Water	144	125
**B-factors (Å^2)**		
Average	22.05	25.24
Protein	20.16	22.97
Ligands	41.71	49.24
Solvent	33.93	40.39
**R.m.s. deviation from ideal**		
Bond lengths (Å)	0.006	0.019
Bond angles (°)	0.80	1.54
**MolProbity analysis**		
Ramachandran favored (%)	99.19	99.16
Ramachandran allowed (%)	0.81	0.84
Ramachandran outliers (%)	0.00	0.00
Rotamer outliers (%)	0.00	0.97
Clash score	1.46	4.07

Statistics for the highest-resolution shell are shown in parentheses. Friedel pairs are counted as one reflection

Appendix Table S3. Cryo-EM data collection and refinement statistics for a complex of the xNup93 α-solenoid with an inhibitory and a tracking nanobody

**Structure**	**xNup93·xhNup93-Nb4i·xNup93-Nb2t**
**PDB ID**	7ZOX
**EMDB ID**	EMD-14849
**Data collection and Processing**	
Detector	Gatan K3
Magnification	105,000
Voltage (Kv)	300
Camera Mode	Counting
Electron exposure ($e^{-}/Å^2$)	54
Defocus range (µm)	1.0-2.5
Pixel size (Å)	0.834
Symmetry imposed	C1
Initial particle images (no.)	808,840
Final particle images (no.)	202,725
Map resolution (Å)	4.4
FSC threshold	0.143
Map resolution range (Å)	4.3-6.5
**Refinement**	
Initial model used (PDB code)	-
Model Resolution (Å)	4.4
FSC threshold	0.5
Map sharpening *B* factor ($Å^2$)	-223
Model composition	
Non-hydrogen atoms	67,778
Protein residues	852
Ligand/ion	0
B factors ($Å^2$)	
Protein	131.79
Ligand/ion	-
R.m.s. deviations	
Bond lengths (Å)	0.005
Bond angles (°)	0.708
**Validation**	
MolProbity score	2.2
Clashscore	16.59
EMRinger score	1.89
Map CC (CC mask)	0.8
Poor rotamers (%)	0.14
Ramachandran analysis (%)	
Preferred	92.34
Allowed	7.66
Outliers	0

Appendix Table S4. X-ray data collection and refinement statistics for Nup98 APD complexes with inhibitory nanobodies

**Structure**	**xNup98 APD·xhNup98-Nb2i**	**xNup98 APD·xhNup98-Nb3i**
**PDB data entry**	7NQA	7NOW
**Space group**	$P2_12_12_1$	C2
**Unit cell dimensions**		
a, b, c (Å)	83.76, 96.63, 100.87	113.8, 149.8, 85.1
α, β, γ (º)	90.00, 90.00, 90.00	90.00, 113.1, 90.00
**Resolution (Å)**	48.3-2.2 (2.26-2.2)	47.8-1.85 (1.90-1.85)
**Mosaicity (°)**	0.18	0.19
**R_{meas}**	0.125 (>1)	0.07 (>1)
**$R_{p.i.m.}$**	0.034 (0.474)	0.029 (1.66)
**<I/σ(I)>**	21.2 (1.9)	16.9 (0.64)
**$CC_{1/2}$**	99.9 (72.2)	99.9 (39.5)
**Reflections**		
Measured	552688	597716
Unique	41309	88309
**Completeness**	97.9 (89.2)	99.3 (98.4)
**Multiplicity**	13.9 (12.3)	6.8 (5.9)
**Wilson B factor (Å^2)**	46.8	45.5
**No of reflections**		
Work set	39261	79698
Test set	2065	1848
**R_{work}**	0.176 (0.292)	0.184 (0.466)
**R_{free}**	0.226 (0.314)	0.209 (0.478)
**R.m.s. deviation from ideal**		
Bond lengths (Å)	0.007	0.007
Bond angles (°)	0.806	0.896
**Ramachandran statistics (%)**		
Favored	96.5	97.1
Allowed	3.1	2.9
Outliers	0.4	0
**<B-factor> (A^2)**	55.8	54.5

Appendix Table S5. Plasmids for nanobody expression in *E.coli*

Plasmid#	Expressed protein	Use	Addgene
pMSC168	H_{14}-NEDD8-xhNup35-Nb1t (+2Cys*; NT, CT)	IF	216350
pMSC227	H_{14}-NEDD8-xhNup35-Nb1t (+3Cys*; NT, S7, CT)	IF	216351
pDG04477	H_{14}-NEDD8-xhNup35-Nb1t	Crystallization	216352
pTP487	H_{14}-NEDD8-xNup62-Nb1t (+3Cys; NT, S7, CT)	IF	216353
pMSC96	H_{14}-NEDD8-xNup93-Nb1t	NPC-AI (non-inhibitory control)	216354
pTP416	H_{14}-NEDD8-xNup93-Nb1t (+1Cys; CT)	IF	216355
pMSC115	H_{14}-NEDD8-xNup93-Nb2t	Crystallization, cryo-EM	216356
pMSC234	H_{14}-NEDD8-xhNup93-Nb3t (+2Cys; NT, CT)	IF	216357
pMSC98	H_{14}-NEDD8-xhNup93-Nb4i	Crystallization, cryo-EM	216358
pMSC262	H_{14}-Avi-$SUMO^{Eu1}$-xhNup93-Nb4i	NPC-AI, AC	216359
pMSC233	H_{14}-NEDD8-xhNup93-Nb4i (+2Cys; NT, CT)	IF	216360
pTP443	H_{14}-NEDD8-xNup98-Nb1t (+1Cys; NT)	IF	216361
pMSC129	H_{14}-NEDD8-xhNup98-Nb2i	Crystallization, NPC-AI, AC	216362
pMSC264	H_{14}-Avi-$SUMO^{Eu1}$-xhNup98-Nb2i	NPC-AI, AC	216363
pMSC130	H_{14}-NEDD8-xhNup98-Nb3i	Crystallization, NPC-AI	216364
pDG04439	H_{14}-NEDD8-xhNup133-Nb2t (+2Cys; NT, CT)	IF	216365
pTP489	H_{14}-NEDD8-xNup155-Nb1t (+3Cys; NT, S7, CT)	IF	216366
pMSC263	H_{14}-Avi-$SUMO^{Eu1}$-xhNup155-Nb2i	NPC-AI, AC	216367
pMSC236	H_{14}-NEDD8-xhNup155-Nb2i (+2Cys; NT, CT)	IF	216368
pMSC100	H_{14}-NEDD8-xhNup155-Nb2i	NPC-AI	216369
pMSC99	H_{14}-NEDD8-xhNup155-Nb3i	NPC-AI	216370
pMSC261	H_{14}-Avi-$SUMO^{Eu1}$-xhNup155-Nb3i	NPC-AI, AC	216371
pMSC235	H_{14}-NEDD8-xhNup155-Nb3i (+2Cys; NT, CT)	IF	216372
pDG04163	H_{14}-scSUMO-Cys-xhNup214-Nb1t-Cys	IF	216373
pTP728	H_{14}-NEDD8-xNup358-Nb1t (+3Cys, NT, S7, CT)	IF	216374
pMSC226	H_{14}-NEDD8-xhNup358-Nb2t (+3Cys; NT, S7, CT)	IF	216375
pTP789	H_{14}-NEDD8-xY-Nb1t (+2Cys; NT, S7)	IF	216376

IF, Immunofluorescence; NPC-AI, NPC assembly inhibition experiments; AC, affinity chromatography; *extra cysteines for fluorophore-maleimide-labelling, introduced as indicated on either N-terminus (NT), C-terminus (CT) or by mutating S7 to cysteine.
