# Supplementary material for: A checkpoint function for Nup98 in nuclear pore formation suggested by novel inhibitory nanobodies
Source: EMBO J. 2024 Apr 22;43(11):6. doi: 10.1038/s44318-024-00081-w (PMC11148069; doi:10.1038/s44318-024-00081-w)
Supplement: Supplementary file 2 — Source data Fig. 1 [file 44318_2024_81_MOESM2_ESM.zip › README_Source_Data_Sola Colom et al.pdf]

Fig. 1C

Folder contains microscopic images for the six time points shown. Each TIF file is a composite of four (16-bit) channels:

- Channel 1: xNup155-Nb1t (Al568)
- Channel 2: xNup93Nb1t (Al488)
- Channel 3: DAPI, not included in the figure
- Channel 4: xY-Nb1t (Al647)

Fig. 1D

Folder contains microscopic images for the six time point with the following channels:

- Channel 1: xNup98-Nb1t (Al568)
- Channel 2: import substrate (IBB-MBP-GFP). Import substrate is shown in the figure both in green and false color (LUT "Fire").
- Channel 3: DAPI, not included in the figure
- Channel 4: xY-Nb1t (Al647)

Fig. 3B

Folder includes 3-channel images for the nuclear mid-plane images (Fig. 3B - top) and Z-stacks of 3-channel images of the nuclear surface (Fig. 3B - bottom). For the images of the nuclear surface, Z-projections with MAX intensity are shown in the figure.

- Channel 1: GFP-Nup107, shown as a green LUT in the figure.
- Channel 2: staining with xY-Nb1t (Al568), shown as a gray LUT in the figure.
- Channel 3: staining with xNup155-Nb1t (Al647), shown as a magenta LUT in the figure.

Fig. 4

Folder includes 3-channel images with the same LUTs as shown in the figure. All the images are 4 μm^2 ROIs of maximum intensity projections of the recorded Z-stacks. The same brightness and contrast settings were applied to all the images on the same panel.

The folder also includes the FiJi script that was used to detect and count old (GFP+) and newly-assembled NPCs.

Fig. 4A

xY-Nb1t & xNup155-Nb1t:

- Channel 1: sfGFP-Nup107
- Channel 2: xNup155-Nb1t (Al568)
- Channel 3: xY-Nb1t (Al647)

xNup155-Nb1t & xNup93-Nb1t:

- Channel 1: sfGFP-Nup107
- Channel 2: xNup155-Nb1t (Al568)
- Channel 3: xNup93-Nb1t (Al647)

xY-Nb1t & xNup62-Nb1t:

- Channel 1: sfGFP-Nup107
- Channel 2: xY-Nb1t (Al568)
- Channel 3: xNup62-Nb1t (Al647)

xY-Nb1t & xNup358-Nb1t:

- Channel 1: sfGFP-Nup107
- Channel 2: xY-Nb1t (Al568)
- Channel 3: xNup358-Nb1t (Al647)

Fig. 4B

xNup155-Nb1t & xNup98-Nb1t:

- Channel 1: sfGFP-Nup107
- Channel 2: xNup155-Nb1t (Al568)
- Channel 3: xNup98-Nb1t (Al647)

xY-Nb1t & xNup153 Ab:

- Channel 1: sfGFP-Nup107
- Channel 2: xNup155-Nb1t (Al568)
- Channel 3: xNup98-Nb1t (Al647)

Fig. 4C

xY-Nb1t & xhNup35-Nb1t:

- Channel 1: sfGFP-Nup107
- Channel 2: xNup35-Nb1t (Al568)
- Channel 3: xY-Nb1t (Al647)

xY-Nb1t & xhNup214-Nb1t:

- Channel 1: sfGFP-Nup107
- Channel 2: xY-Nb1t (Al568)
- Channel 3: xNup214-Nb1t (Al647)

Fig. 5 A, B

The folder contain the STED images used in the figures, along with an additional replicate for Fig. 5A and 5B. The first channel nanobodies are coupled to Abberior STAR 645P and visualized with a magenta LUT, while the second channel detects nanobodies coupled to Abberior STAR 580 with a green LUT.

- Channel 1: nanobodies coupled to Abberior STAR 645P and visualized with a magenta LUT
- Channel 2: nanobodies coupled to Abberior STAR 580 and visualized with a green LUT.

Folder also contains images detailing the ring measurements. and the raw data used for quantification. Statistical analyses were conducted using an unpaired t-test (parametric), where a p-value <0.0001 is denoted by "****"

Fig. 5C

It contains images detailing the ring measurements and the raw data necessary for quantification analyses. The statistical analyses were conducted using an unpaired t-test (parametric), where a p-value <0.0001 is denoted by "****".

Fig. 6A

3-channel images are included: Channel 1 corresponds to the GFP signal from the genomically-tagged GFP*Nup107, which was used as a reference signal. Channels 2 and 3 correspond to stainings using the indicated nanobodies (Alexa fluor 647) acquired at different laser intensities. In all cases, the images of channel 2 were used for the figure with the same brightness and contrast settings being applied.

Fig. 6B

The folder contains images that have been deconvolved using Huygens software. For each tracking nanobody, it includes the images displayed in the figure as well as four additional replicates. All nanobodies were coupled to Abberior-635P.

The diameter of the NPC rings was measured using the FiJi software. Images with measurements and raw data of the measurements are also included. Statistical analysis was by an unpaired t-test (parametric). **** indicates a p-value of <0.0001.

Fig. 6C

The folder contains the raw data corresponding to the measurements of NPC rings.

The diameter of the NPC rings was measured using the FiJi software. Images with measurements and raw data of the measurements are also included. Statistic analysis was by an unpaired t-test (parametric). **** indicates a p-value of <0.0001.

Fig. 6D

The folder contains the image of the original gel scan and the image after having adjusted the brightness and contrast as it appears in the figure. Both images are included in .tif format. The (proportional) brightness adjustments were done using Photoshop.

Fig. 7ABC

The folder contains the images shown in Figures 7A, B, and C, as well as three additional image examples for documenting the phenotype of xhNup93-Nb4i.

3-channel images are included for active import of IBB-MBP-GFP and passive exclusion of MBP-mCherry:

- Channel 1: DAPI
- Channel 2: import substrate (IBB-MBP-GFP), shown in the figure with "Green" and "Fire" LUTs.
- Channel 3: passive exclusion (MBP-mCherry), shown in the figure with the "Hot Orange" LUT.

2-channel images are included for the active exclusion of NES-GFP:

- Channel1: DAPI
- Channel 2: active exclusion (NES-GFP), shown in the figure with a yellow LUT.

Fig. 8A

Images are located in subfolders according to the tracking nanobodies. File names begin with the name of the tracking nanobody/nanobodies followed by the name of the inhibitory nanobody used.

All images are included as multi-channel composites. Some of the channels were not included in the manuscript figure, but are also described below and serve as replicate images.

xNup93-Nb1t & xhNup35-Nb1t

- Channel 1: xY-Nb1t (Al568), not shown in the figure.
- Channel 2: xNup93-Nb1t (Al488), shown with the “Cyan Hot ” LUT (the same as in the figure).
- Channel 3: DAPI, not shown in the figure.
- Channel 4: xhNup35-Nb1t (Al647), shown in a magenta LUT (the same as in the figure).

xY-Nb1t, xNup62-Nb1t, xhNup214-Nb1t

- Channel 1: xY-Nb1t (Al568), shown with a Cyan LUT
- Channel 2: xNup62-Nb1t (Al488), shown with a Green LUT.
- Channel 3: xhNup214-Nb1t (Al647), shown with a Orange LUT.
- Channel 4: DAPI, not shown in the figure

xNup98-Nb1t

- Channel 1: xY-Nb1t (Al568), not shown in the figure
- Channel 2: xNup62-Nb1t (Al488), also not shown in the figure
- Channel 3: DAPI, also not shown in the figure
- Channel 4: xNup98-Nb1t (Al647), shown with a Red LUT as in the figure.

xNup155-Nb1t

- Channel 1: xY-Nb1t (Al568), not shown in the figure.
- Channel 2: xNup93-Nb1t (Al488), not shown in the figure.
- Channel 3: DAPI, not shown in the figure.
- Channel 4: Nup155 (Al647). Shown with a magenta LUT as in the figure.

xNup358-Nb1t

- Channel 1: xY-Nb1t (Al568), not shown in the figure.
- Channel 2: xhNup214-Nb1t (Al488), not shown in the figure.
- Channel 3: xNup358-Nb1t (Al647), shown with a Gray LUT as in the figure.
- Channel 4: DAPI

Fig. 8B

Images are in subfolders according to the tracking nanobodies. Image names begin with the name of the tracking nanobody/nanobodies followed by the name of the inhibitory nanobody used. All images are included as multi-channel composites. Some of the channels were not included in the manuscript figure, but are also described below and serve as replicate images. The images are included as Z stacks. In the figure, all images are shown as Maximum intensity projections obtained by FiJi and displayed with the same values of brightness and contrast.

xNup93-Nb1t & xhNup35-Nb1t

- Channel 1: xNup93-Nb1t (Al488). Images shown with the “Cyan Hot ” LUT as in the figure.
- Channel 2: xhNup35-Nb1t (Al568). Images shown in a magenta LUT as in the figure.
- Channel 3: DAPI, not included in the figure.
- Channel 4: xY-Nb1t (Al647), not shown in the figure.

xNup155-Nb1t & xNup98-Nb1t

- Channel 1: xNup155-Nb1t (Al488). Shown as in the figure with a Yellow LUT.
- Channel 2: xNup98-Nb1t (Al568). Shown with a Red LUT as in the figure.
- Channel 3: DAPI, not included in the figure.
- Channel 4: xY-Nb1t (Al647), not shown in the figure.

xNup358-Nb1t

- Channel 1: xNup358-Nb1t (Al568), shown in the figure with a Gray LUT.
- Channel 2: xhNup214-Nb1t (Al488), not shown in the figure.
- Channel 3: DAPI, not shown in the figure.
- Channel 4: xY-Nb1t (Al647), not shown in the figure.

xY-Nb1t, xNup62-Nb1t and xhNup214-Nb1t

- Channel 1: xY-Nb1t (Al568), shown with a Cyan LUT as in the figure.
- Channel 2: xNup62-Nb1t (Al488), shown with a green LUT as in the figure.
- Channel 3: xhNup214-Nb1t (Al647), shown in the figure with an orange LUT.
- Channel 4: DAPI, not shown in the figure.

Fig. 9

The source data includes five spreadsheets, corresponding to the numerical data of the measurements of pores detected by the 5 different tracking nanobodies (i.e. each file is a row in Figure 9). In each spreadsheet file, each row represents a counted pore, and the different columns indicate:

- **Experiment date:** Two independent experiments for each tracking nanobody are included.
- **Mean_tracking nanobody:** Measured mean intensity on the tracking nanobody channel
- **Normalized_mean_tracking nanobody**: Measured mean intensity of the respective pore on the tracking nanobody channel - normalized to the average intensity of the buffer control samples.
- **Ch_tracking nanobody**: microscope channel where this image was acquired.
- **Mean_ch_Ycmplx**: Mean intensity of the respective pore on the xY-Nb1t channel.
- **Normalized_mean_ch_Y complex**: Mean intensity on the xY-Nb1t channel - normalized by the average intensity of the Buffer (i.e. control) samples.
- **Ch_Y complex**: microscope channel where the Y complex images were acquired.
- **Inhibitory Nb**: Indicates the inhibitory nanobody or control in the presence of which the nuclei were assembled.
- **# Nucleus**: Nucleus index from which the NPC was acquired. Between 3 and 7 nuclei per inhibitory nanobody were included in the analysis.
- **Average ch tracking nanobody**: Mean of the buffer mean intensity values on the tracking nanobody channel that was used for the normalizations.
- **Average ch Y complex**: Mean of the Buffer mean intensity values on the Y complex nanobody channel that was used for the normalizations.

The folder also includes the FiJi script that was used to detect, count NPCs and measure the signal intensities.

Fig. 10A

Folder includes three 4-channel images.

- Channel 1: DAPI, not included in the figure.
- Channel 2: xhNup358-Nb2t (Al568), shown with a magenta LUT in the figure.
- Channel 3: xhNup133-Nb2t (Al488), with a gray LUT shown in the figure.
- Channel 4: xhNup93-Nb4i (Al647), shown with a green LUT in the figure.

Besides the two images shown in Fig. 10A, an additional replicate image is included showing the increase of the xhNup93-Nb4i signal in Nup358-silenced nuclei.

Fig. 11C

Gels are labeled as Nup98_Nup96 for the one shown on the left on the figure and Nup98_Nup88 for the one shown on right in figure 11D. For the final figure, the gel ladders and not relevant lanes on the gel edges were cropped. The brightness was adjusted using Photoshop.

Fig. 12

3-channel images with the same LUTs as shown in the figure. All the images are 4 μm^2 of maximum intensity projections of the acquired Z-stacks. The same brightness and contrast settings were applied to all the images of the same panel.

Fig. 12A

- Channel1: sfGFP-Nup107
- Channel2: xY-Nb1t (Al568)
- Channel3: xNup155-Nb1t (Al647)

Fig. 12B

- Channel1: sfGFP-Nup107
- Channel2: xY-Nb1t (Al568)
- Channel3: xNup98-Nb1t (Al647)

Fig. EV1A

Folder includes raw data (.czi files) of the acquired Z-stacks. This format is readable in FiJi or ImageJ. Four examples of images of HeLa nuclei with and without frog egg extract incubation are included. The images were used for the calculation of the nuclei volume. The raw data for the volume calculations is also included. The 3D-reconstructions shown in figure EV1 were obtained using the Arivis software.

Fig. EV1B

The images from EV1A were used for the calculation of the nuclei volume. The raw data for the volume calculations is included in a spreadsheet format.

The spreadsheet contains two tabs corresponding to the “No extract” and “Extract” samples. Each row corresponds to the Area measurement of each of the nuclei Z stacks that was then used to estimate the volume. All measurements were done using FiJi.

Fig. EV1C

Contains the raw data for the measurements of the number of new inserted pores in a spreadsheet format. The number of detected new pores, GFP+ pores using FiJi, and the % that the new pores represent is indicated. Data from different experiments is included in different tables.

Fig. EV3

Folder includes 3-channel images with the same LUTs as shown in the figure. All the images are 4 μm^2 ROIs of maximum intensity projections of the recorded Z-stacks. The same brightness and contrast settings were applied to all the images on the same panel.

- Channel1: sfGFP-Nup107
- Channel2: hNdc1 or hGp210 Ab (Al568)
- Channel3: xY-Nb1t (Al647)

Fig. EV5B

Microscopic images. Images are named as the corresponding inhibitory nanobody and the time point at which it was added to the assembly reaction. Three images corresponding to 3 different channels are included as a composite in the same .tif file:

- Channel 1: DAPI
- Channels 2 and 3: import substrate-GFP acquired at different laser intensities. Channel 2 was used for the figure with the Green LUT and also shown with a false color (i.e. LUT “Fire”).
