# Supplementary figures and images for: A checkpoint function for Nup98 in nuclear pore formation suggested by novel inhibitory nanobodies (part 1 of 2)
Source: EMBO J. 2024 Apr 22;43(11):6. doi: 10.1038/s44318-024-00081-w (PMC11148069; doi:10.1038/s44318-024-00081-w)

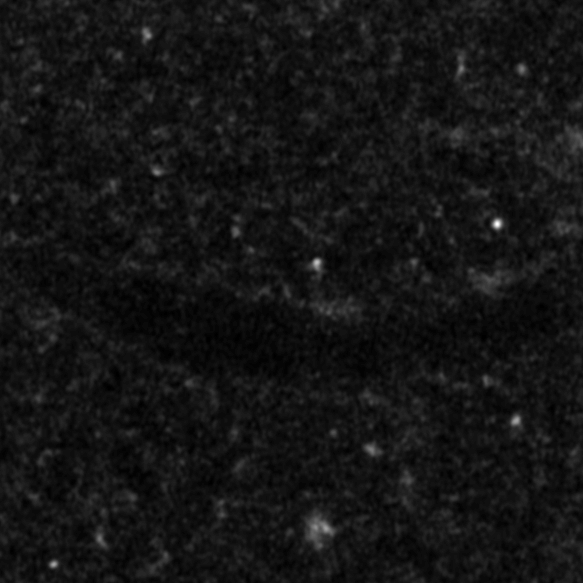

Supplement: Supplementary file 2 — Source data Fig. 1 [file 44318_2024_81_MOESM2_ESM.zip › Fig1C/Fig1C-0mins.tif]

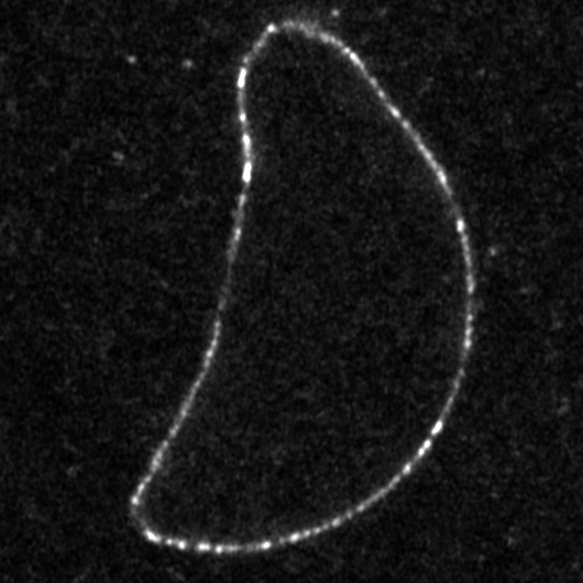

Supplement: Supplementary file 2 — Source data Fig. 1 [file 44318_2024_81_MOESM2_ESM.zip › Fig1C/Fig1C-120mins.tif]

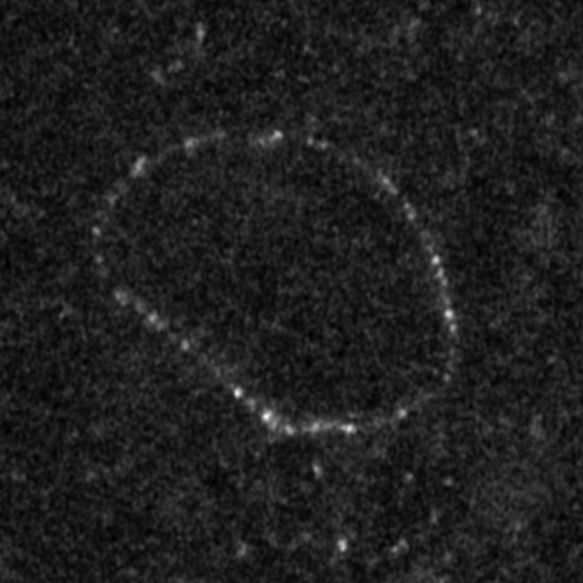

Supplement: Supplementary file 2 — Source data Fig. 1 [file 44318_2024_81_MOESM2_ESM.zip › Fig1C/Fig1C-15mins.tif]

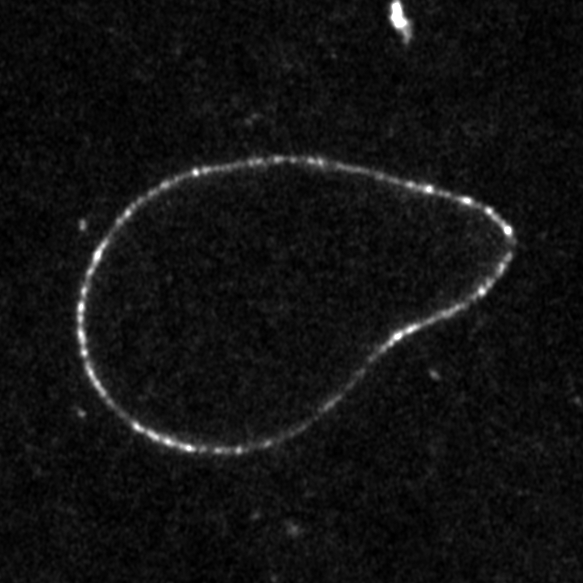

Supplement: Supplementary file 2 — Source data Fig. 1 [file 44318_2024_81_MOESM2_ESM.zip › Fig1C/Fig1C-30mins.tif]

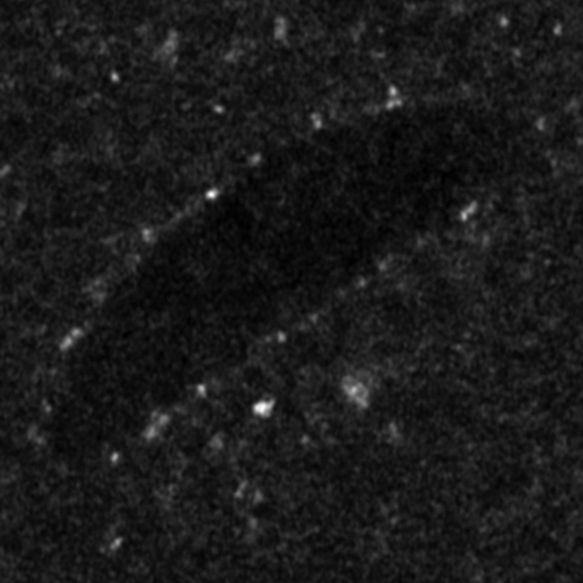

Supplement: Supplementary file 2 — Source data Fig. 1 [file 44318_2024_81_MOESM2_ESM.zip › Fig1C/Fig1C-5mins.tif]

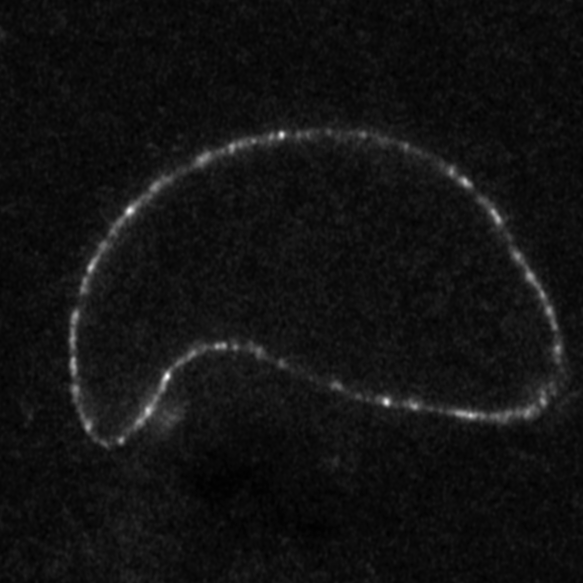

Supplement: Supplementary file 2 — Source data Fig. 1 [file 44318_2024_81_MOESM2_ESM.zip › Fig1C/Fig1C-60mins.tif]

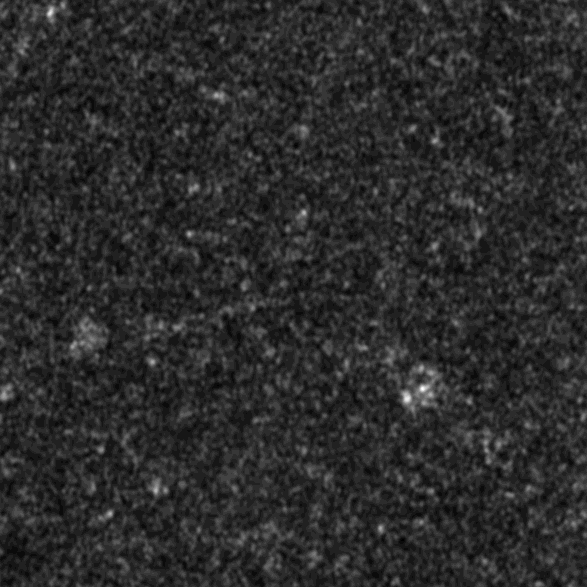

Supplement: Supplementary file 2 — Source data Fig. 1 [file 44318_2024_81_MOESM2_ESM.zip › Fig1D/Fig1D-0mins.tif]

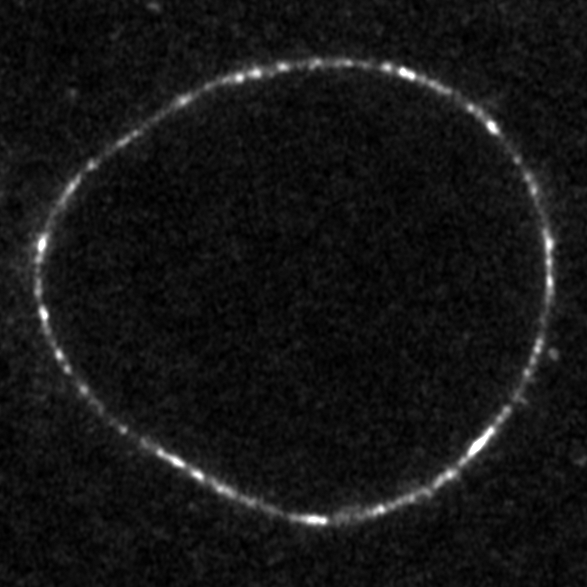

Supplement: Supplementary file 2 — Source data Fig. 1 [file 44318_2024_81_MOESM2_ESM.zip › Fig1D/Fig1D-120mins.tif]

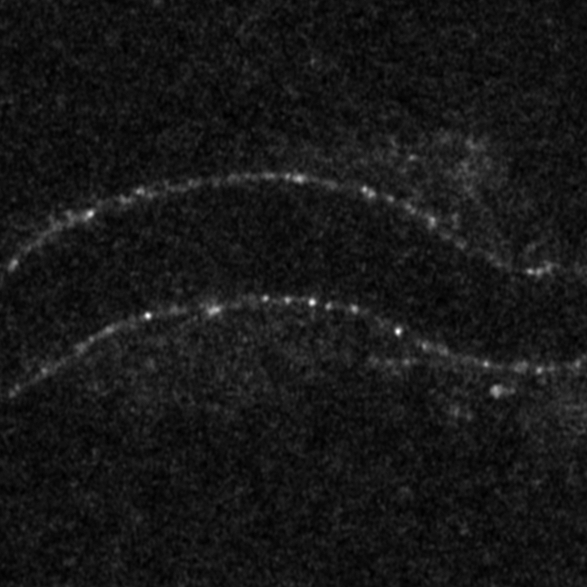

Supplement: Supplementary file 2 — Source data Fig. 1 [file 44318_2024_81_MOESM2_ESM.zip › Fig1D/Fig1D-15mins.tif]

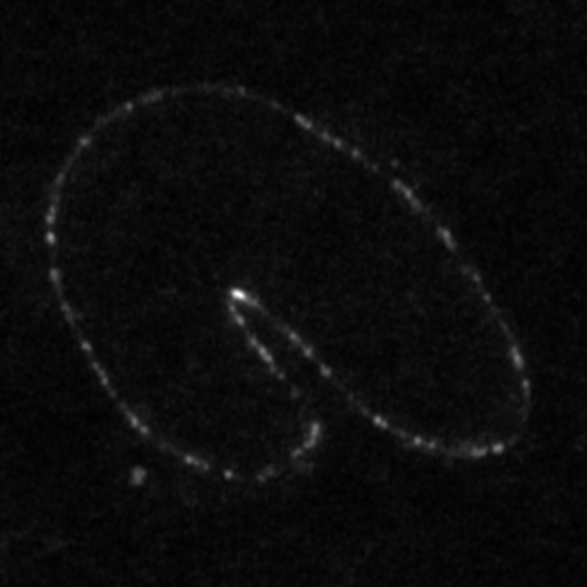

Supplement: Supplementary file 2 — Source data Fig. 1 [file 44318_2024_81_MOESM2_ESM.zip › Fig1D/Fig1D-30mins.tif]

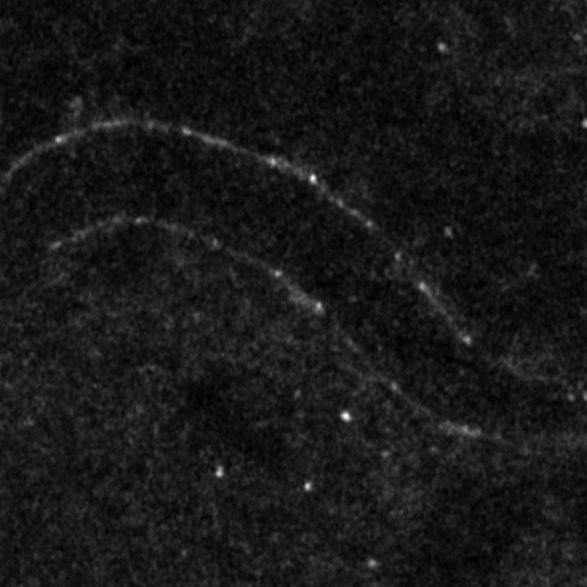

Supplement: Supplementary file 2 — Source data Fig. 1 [file 44318_2024_81_MOESM2_ESM.zip › Fig1D/Fig1D-5mins.tif]

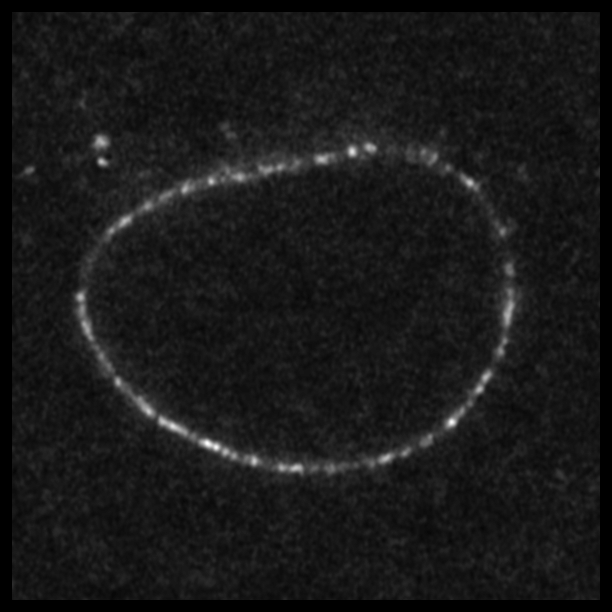

Supplement: Supplementary file 2 — Source data Fig. 1 [file 44318_2024_81_MOESM2_ESM.zip › Fig1D/Fig1D-60mins.tif]

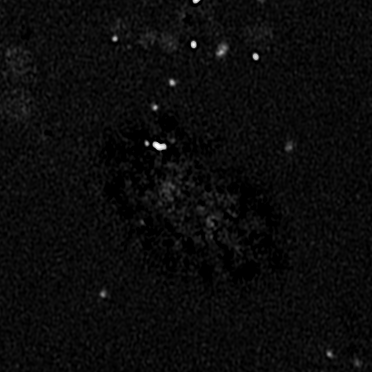

Supplement: Supplementary file 3 — Source data Fig. 3 [file 44318_2024_81_MOESM3_ESM.zip › Fig3B/Fig3B_bottom_Extract.tif]

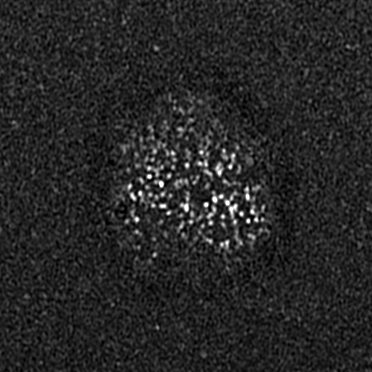

Supplement: Supplementary file 3 — Source data Fig. 3 [file 44318_2024_81_MOESM3_ESM.zip › Fig3B/Fig3B_bottom_Noextract.tif]

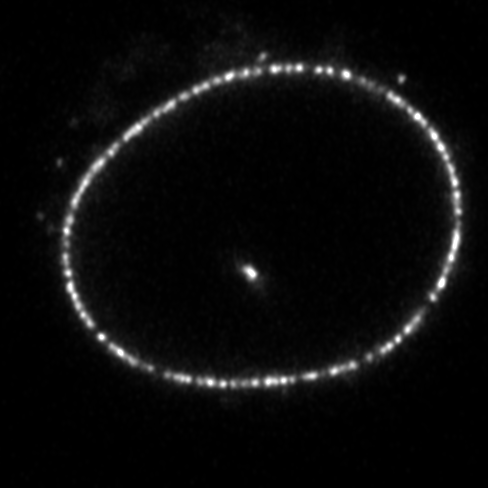

Supplement: Supplementary file 3 — Source data Fig. 3 [file 44318_2024_81_MOESM3_ESM.zip › Fig3B/Fig3B_middle_plane_Extract.tif]

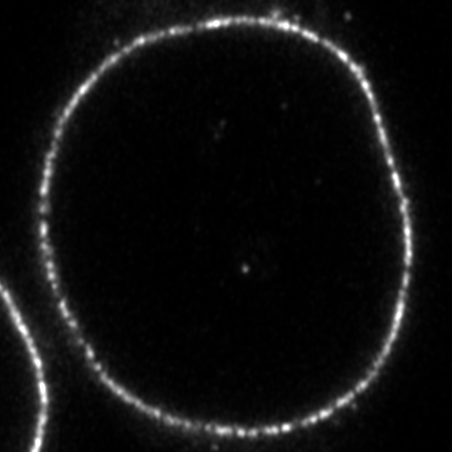

Supplement: Supplementary file 3 — Source data Fig. 3 [file 44318_2024_81_MOESM3_ESM.zip › Fig3B/Fig3B_middle_plane_NoExtract.tif]

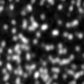

Supplement: Supplementary file 4 — Source data Fig. 4 [file 44318_2024_81_MOESM4_ESM.zip › Fig4A/Fig4A-Ycmplx_Nup155_Extract.tif]

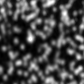

Supplement: Supplementary file 4 — Source data Fig. 4 [file 44318_2024_81_MOESM4_ESM.zip › Fig4A/Fig4A-Ycmplx_Nup155_NoExtract.tif]

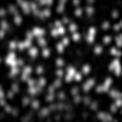

Supplement: Supplementary file 4 — Source data Fig. 4 [file 44318_2024_81_MOESM4_ESM.zip › Fig4A/Fig4A_Nup155_Nup93_Extract.tif]

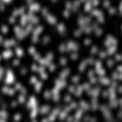

Supplement: Supplementary file 4 — Source data Fig. 4 [file 44318_2024_81_MOESM4_ESM.zip › Fig4A/Fig4A_Nup155_Nup93_Noextract.tif]

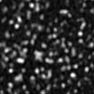

Supplement: Supplementary file 4 — Source data Fig. 4 [file 44318_2024_81_MOESM4_ESM.zip › Fig4A/Fig4A_Ycmplx_Nup358_Extract.tif]

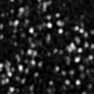

Supplement: Supplementary file 4 — Source data Fig. 4 [file 44318_2024_81_MOESM4_ESM.zip › Fig4A/Fig4A_Ycmplx_Nup358_Noextract.tif]

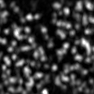

Supplement: Supplementary file 4 — Source data Fig. 4 [file 44318_2024_81_MOESM4_ESM.zip › Fig4A/Fig4A_Ycmplx_Nup62cmplx_Extract.tif]

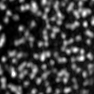

Supplement: Supplementary file 4 — Source data Fig. 4 [file 44318_2024_81_MOESM4_ESM.zip › Fig4A/Fig4A_Ycmplx_Nup62cmplx_Noextract.tif]

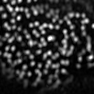

Supplement: Supplementary file 4 — Source data Fig. 4 [file 44318_2024_81_MOESM4_ESM.zip › Fig4B/Fig4B-Ycmplx_Nup153-Extract.tif]

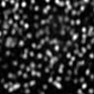

Supplement: Supplementary file 4 — Source data Fig. 4 [file 44318_2024_81_MOESM4_ESM.zip › Fig4B/Fig4B-Ycmplx_Nup153-NoExtract.tif]

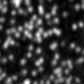

Supplement: Supplementary file 4 — Source data Fig. 4 [file 44318_2024_81_MOESM4_ESM.zip › Fig4B/Fig4B_Nup155_Nup98_Extract.tif]

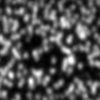

Supplement: Supplementary file 4 — Source data Fig. 4 [file 44318_2024_81_MOESM4_ESM.zip › Fig4B/Fig4B_Nup155_Nup98_Noextract.tif]

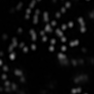

Supplement: Supplementary file 4 — Source data Fig. 4 [file 44318_2024_81_MOESM4_ESM.zip › Fig4C/Fig4C-Ycmplx_Nup214-Extract.tif]

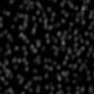

Supplement: Supplementary file 4 — Source data Fig. 4 [file 44318_2024_81_MOESM4_ESM.zip › Fig4C/Fig4C-Ycmplx_Nup214-NoExtract.tif]

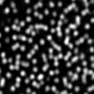

Supplement: Supplementary file 4 — Source data Fig. 4 [file 44318_2024_81_MOESM4_ESM.zip › Fig4C/Fig4C-Ycmplx_Nup53-Extract.tif]

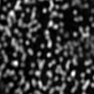

Supplement: Supplementary file 4 — Source data Fig. 4 [file 44318_2024_81_MOESM4_ESM.zip › Fig4C/Fig4C-Ycmplx_Nup53-NoExtract.tif]

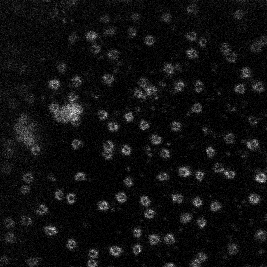

Supplement: Supplementary file 5 — Source data Fig. 5 [file 44318_2024_81_MOESM5_ESM.zip › Fig5A/Fig5A-raw.tif]

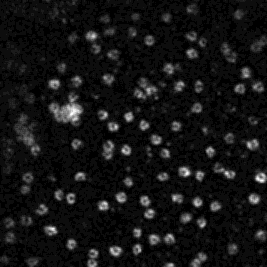

Supplement: Supplementary file 5 — Source data Fig. 5 [file 44318_2024_81_MOESM5_ESM.zip › Fig5A/Fig5A-replicate_raw.tif]

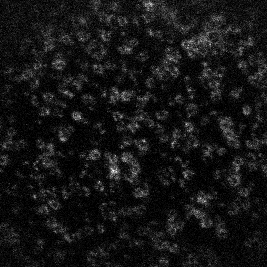

Supplement: Supplementary file 5 — Source data Fig. 5 [file 44318_2024_81_MOESM5_ESM.zip › Fig5B/Fig5B-raw.tif]

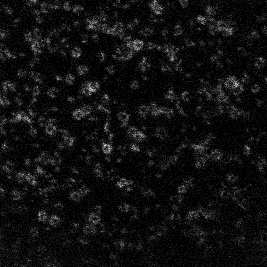

Supplement: Supplementary file 5 — Source data Fig. 5 [file 44318_2024_81_MOESM5_ESM.zip › Fig5B/Fig5B-replicate_raw.tif]

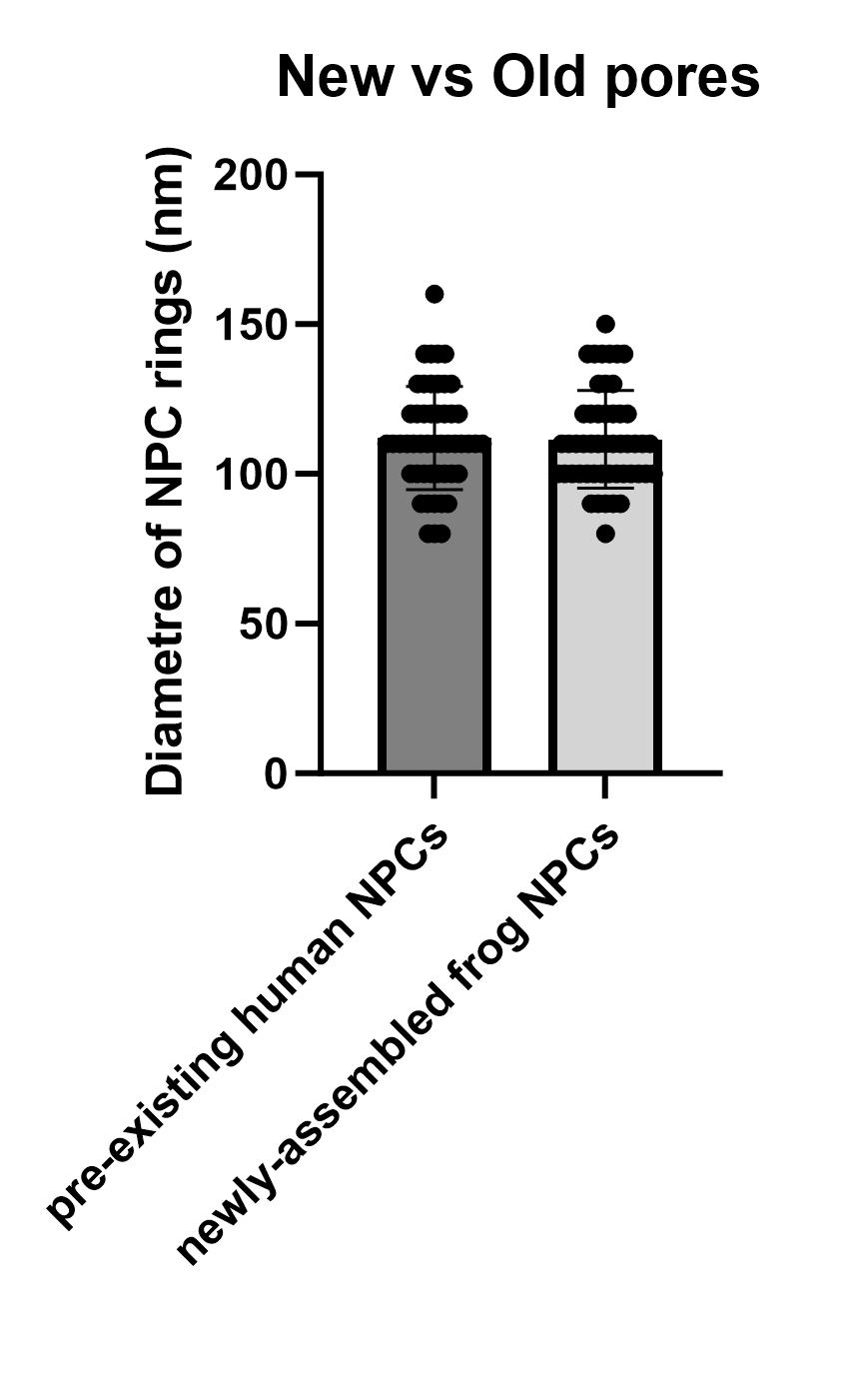

Supplement: Supplementary file 5 — Source data Fig. 5 [file 44318_2024_81_MOESM5_ESM.zip › Fig5C/Fig5C - histogram.jpg]

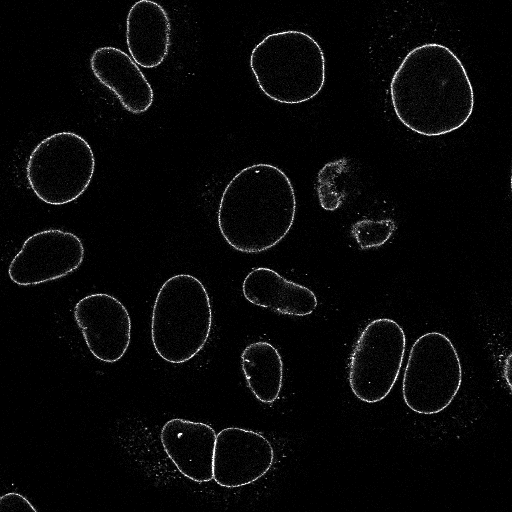

Supplement: Supplementary file 6 — Source data Fig. 6 [file 44318_2024_81_MOESM6_ESM.zip › Fig6A/xhNup133-Nb1t.tif]

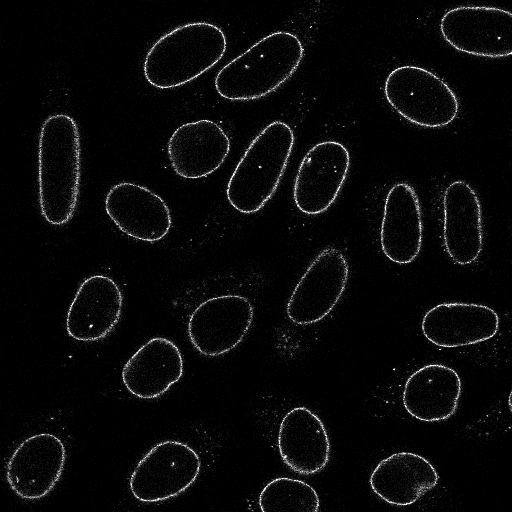

Supplement: Supplementary file 6 — Source data Fig. 6 [file 44318_2024_81_MOESM6_ESM.zip › Fig6A/xhNup155-Nb2i.tif]

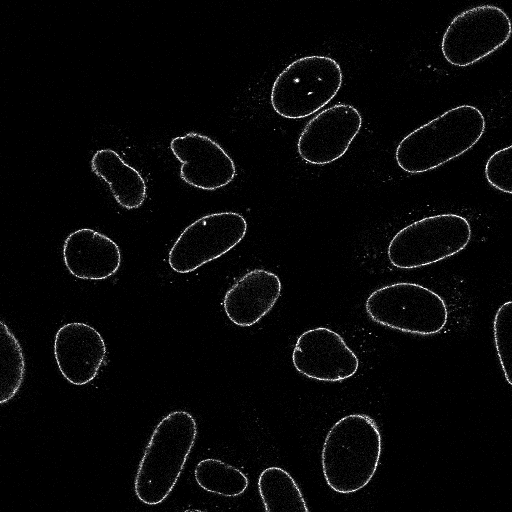

Supplement: Supplementary file 6 — Source data Fig. 6 [file 44318_2024_81_MOESM6_ESM.zip › Fig6A/xhNup155-Nb3i.tif]

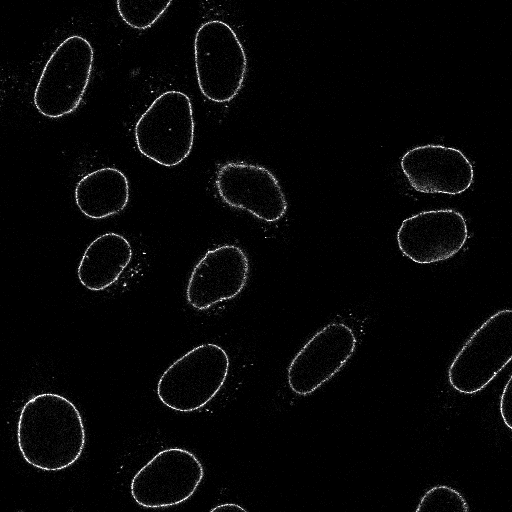

Supplement: Supplementary file 6 — Source data Fig. 6 [file 44318_2024_81_MOESM6_ESM.zip › Fig6A/xhNup35-Nb1t.tif]

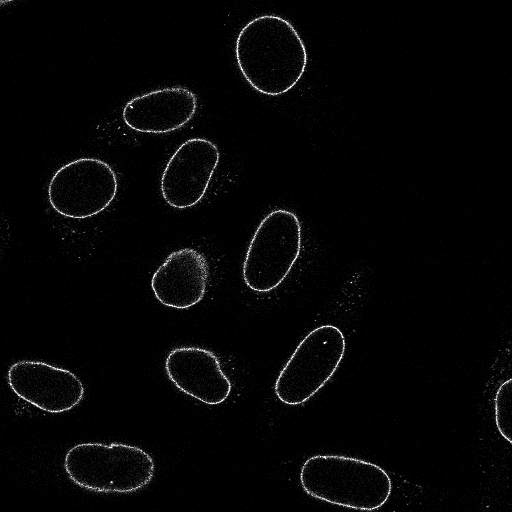

Supplement: Supplementary file 6 — Source data Fig. 6 [file 44318_2024_81_MOESM6_ESM.zip › Fig6A/xhNup358-Nb2t.tif]

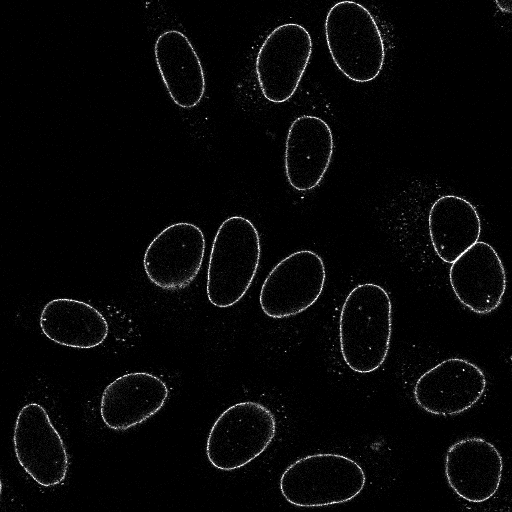

Supplement: Supplementary file 6 — Source data Fig. 6 [file 44318_2024_81_MOESM6_ESM.zip › Fig6A/xhNup93-Nb3t.tif]

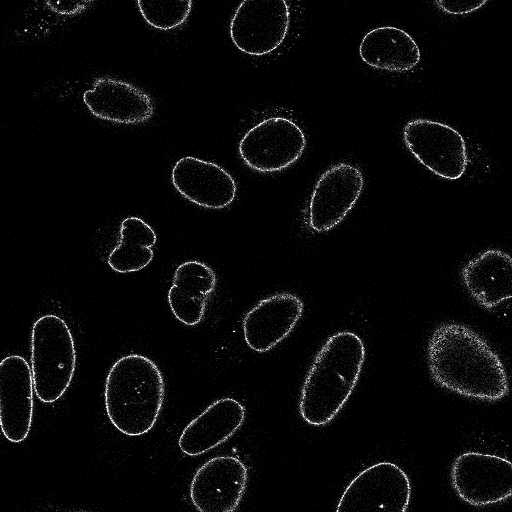

Supplement: Supplementary file 6 — Source data Fig. 6 [file 44318_2024_81_MOESM6_ESM.zip › Fig6A/xhNup93-Nb4i.tif]

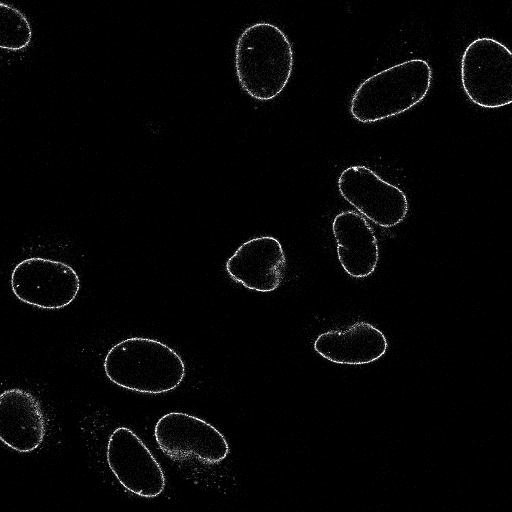

Supplement: Supplementary file 6 — Source data Fig. 6 [file 44318_2024_81_MOESM6_ESM.zip › Fig6A/xhNup98-Nb2i.tif]

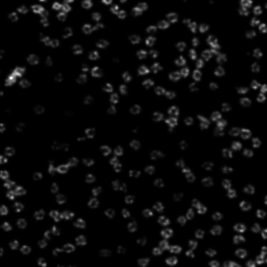

Supplement: Supplementary file 6 — Source data Fig. 6 [file 44318_2024_81_MOESM6_ESM.zip › Fig6B/xhNup133-Nb1t.tif]

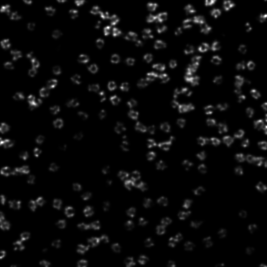

Supplement: Supplementary file 6 — Source data Fig. 6 [file 44318_2024_81_MOESM6_ESM.zip › Fig6B/xhNup133-Nb1t_replicate2.tif]

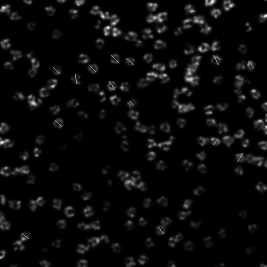

Supplement: Supplementary file 6 — Source data Fig. 6 [file 44318_2024_81_MOESM6_ESM.zip › Fig6B/xhNup133-Nb1t_replicate2_measurements.tif]

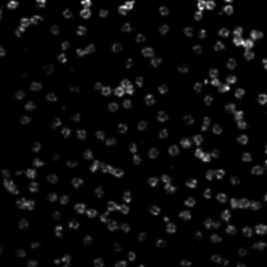

Supplement: Supplementary file 6 — Source data Fig. 6 [file 44318_2024_81_MOESM6_ESM.zip › Fig6B/xhNup133-Nb1t_replicate3.tif]

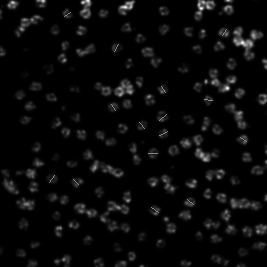

Supplement: Supplementary file 6 — Source data Fig. 6 [file 44318_2024_81_MOESM6_ESM.zip › Fig6B/xhNup133-Nb1t_replicate3_measurements.tif]

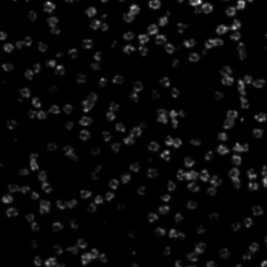

Supplement: Supplementary file 6 — Source data Fig. 6 [file 44318_2024_81_MOESM6_ESM.zip › Fig6B/xhNup133-Nb1t_replicate4.tif]

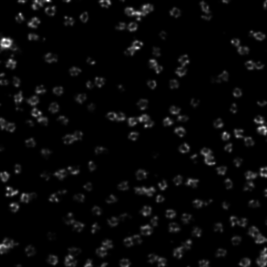

Supplement: Supplementary file 6 — Source data Fig. 6 [file 44318_2024_81_MOESM6_ESM.zip › Fig6B/xhNup133-Nb1t_replicate5.tif]

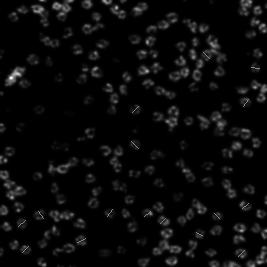

Supplement: Supplementary file 6 — Source data Fig. 6 [file 44318_2024_81_MOESM6_ESM.zip › Fig6B/xhNup133-Nb1t_withmeasurements.tif]

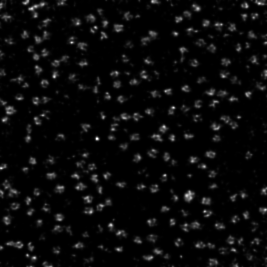

Supplement: Supplementary file 6 — Source data Fig. 6 [file 44318_2024_81_MOESM6_ESM.zip › Fig6B/xhNup35-Nb1t.tif]

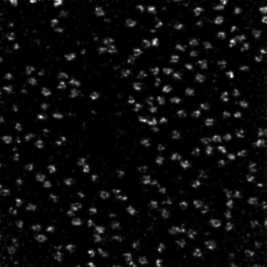

Supplement: Supplementary file 6 — Source data Fig. 6 [file 44318_2024_81_MOESM6_ESM.zip › Fig6B/xhNup35-Nb1t_replicate1.tif]

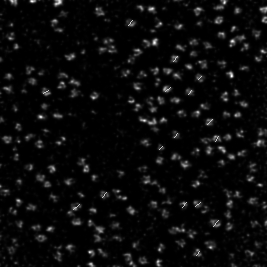

Supplement: Supplementary file 6 — Source data Fig. 6 [file 44318_2024_81_MOESM6_ESM.zip › Fig6B/xhNup35-Nb1t_replicate1_measurements.tif]

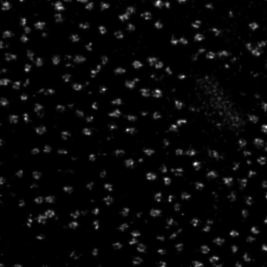

Supplement: Supplementary file 6 — Source data Fig. 6 [file 44318_2024_81_MOESM6_ESM.zip › Fig6B/xhNup35-Nb1t_replicate2.tif]

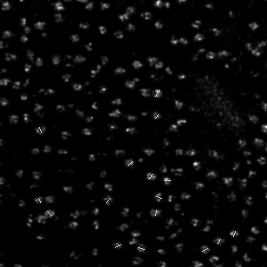

Supplement: Supplementary file 6 — Source data Fig. 6 [file 44318_2024_81_MOESM6_ESM.zip › Fig6B/xhNup35-Nb1t_replicate2_measurements.tif]

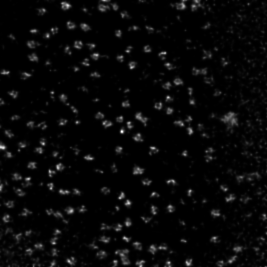

Supplement: Supplementary file 6 — Source data Fig. 6 [file 44318_2024_81_MOESM6_ESM.zip › Fig6B/xhNup35-Nb1t_replicate4.tif]

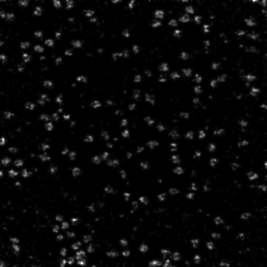

Supplement: Supplementary file 6 — Source data Fig. 6 [file 44318_2024_81_MOESM6_ESM.zip › Fig6B/xhNup35-Nb1t_replicate5.tif]

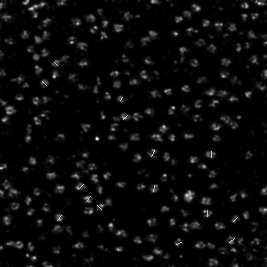

Supplement: Supplementary file 6 — Source data Fig. 6 [file 44318_2024_81_MOESM6_ESM.zip › Fig6B/xhNup35-Nb1t_withmeasurements.tif]

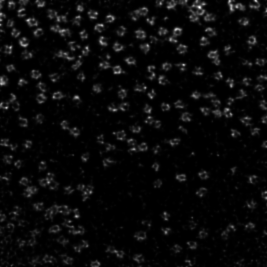

Supplement: Supplementary file 6 — Source data Fig. 6 [file 44318_2024_81_MOESM6_ESM.zip › Fig6B/xhNup358-Nb2t.tif]

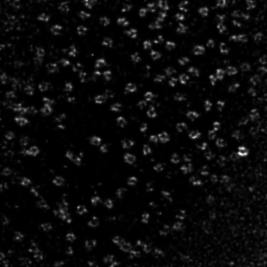

Supplement: Supplementary file 6 — Source data Fig. 6 [file 44318_2024_81_MOESM6_ESM.zip › Fig6B/xhNup358-Nb2t_replicate1.tif]

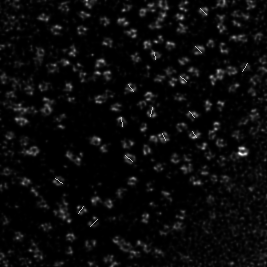

Supplement: Supplementary file 6 — Source data Fig. 6 [file 44318_2024_81_MOESM6_ESM.zip › Fig6B/xhNup358-Nb2t_replicate1_measurements.tif]

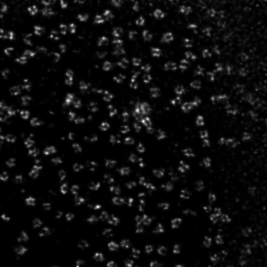

Supplement: Supplementary file 6 — Source data Fig. 6 [file 44318_2024_81_MOESM6_ESM.zip › Fig6B/xhNup358-Nb2t_replicate2.tif]

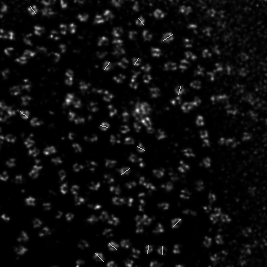

Supplement: Supplementary file 6 — Source data Fig. 6 [file 44318_2024_81_MOESM6_ESM.zip › Fig6B/xhNup358-Nb2t_replicate2_measurements.tif]

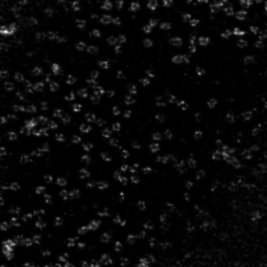

Supplement: Supplementary file 6 — Source data Fig. 6 [file 44318_2024_81_MOESM6_ESM.zip › Fig6B/xhNup358-Nb2t_replicate3.tif]

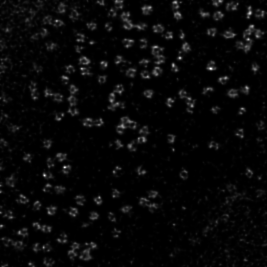

Supplement: Supplementary file 6 — Source data Fig. 6 [file 44318_2024_81_MOESM6_ESM.zip › Fig6B/xhNup358-Nb2t_replicate4.tif]

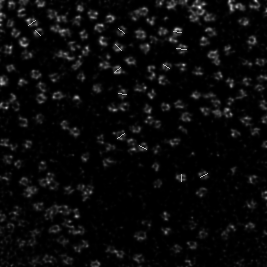

Supplement: Supplementary file 6 — Source data Fig. 6 [file 44318_2024_81_MOESM6_ESM.zip › Fig6B/xhNup358-Nb2t_withmeasurements.tif]

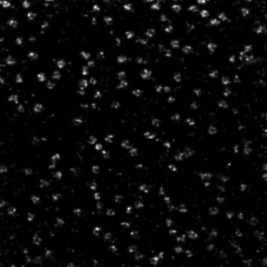

Supplement: Supplementary file 6 — Source data Fig. 6 [file 44318_2024_81_MOESM6_ESM.zip › Fig6B/xhNup93-Nb3t.tif]

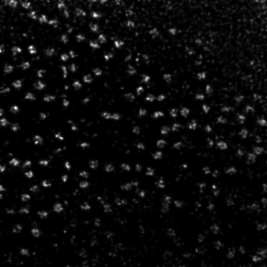

Supplement: Supplementary file 6 — Source data Fig. 6 [file 44318_2024_81_MOESM6_ESM.zip › Fig6B/xhNup93-Nb3t_replicate1.tif]

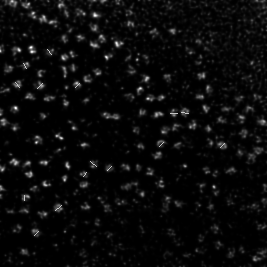

Supplement: Supplementary file 6 — Source data Fig. 6 [file 44318_2024_81_MOESM6_ESM.zip › Fig6B/xhNup93-Nb3t_replicate1_withmeasurements.tif]

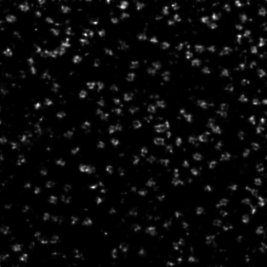

Supplement: Supplementary file 6 — Source data Fig. 6 [file 44318_2024_81_MOESM6_ESM.zip › Fig6B/xhNup93-Nb3t_replicate2.tif]

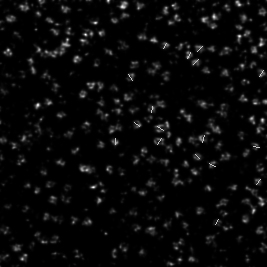

Supplement: Supplementary file 6 — Source data Fig. 6 [file 44318_2024_81_MOESM6_ESM.zip › Fig6B/xhNup93-Nb3t_replicate2_measurements.tif]

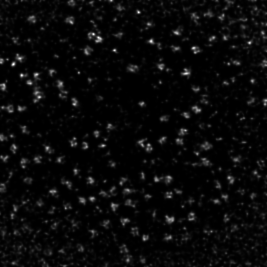

Supplement: Supplementary file 6 — Source data Fig. 6 [file 44318_2024_81_MOESM6_ESM.zip › Fig6B/xhNup93-Nb3t_replicate3.tif]

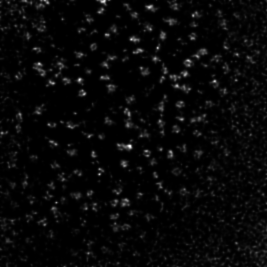

Supplement: Supplementary file 6 — Source data Fig. 6 [file 44318_2024_81_MOESM6_ESM.zip › Fig6B/xhNup93-Nb3t_replicate4.tif]

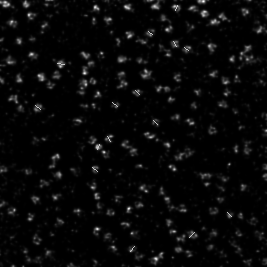

Supplement: Supplementary file 6 — Source data Fig. 6 [file 44318_2024_81_MOESM6_ESM.zip › Fig6B/xhNup93-Nb3t_withmeasurements.tif]

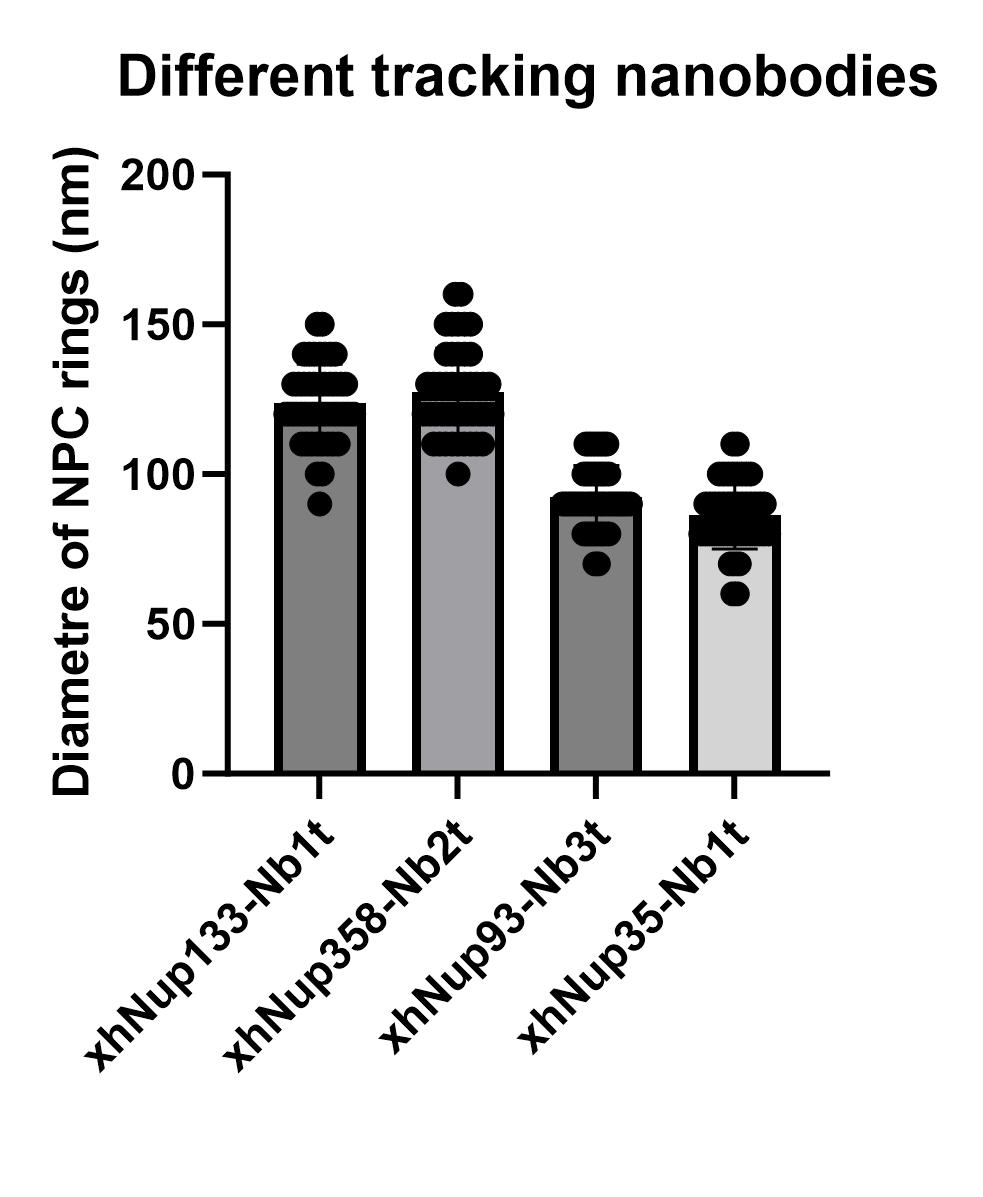

Supplement: Supplementary file 6 — Source data Fig. 6 [file 44318_2024_81_MOESM6_ESM.zip › Fig6C/Diameter of NPC rings.jpg]

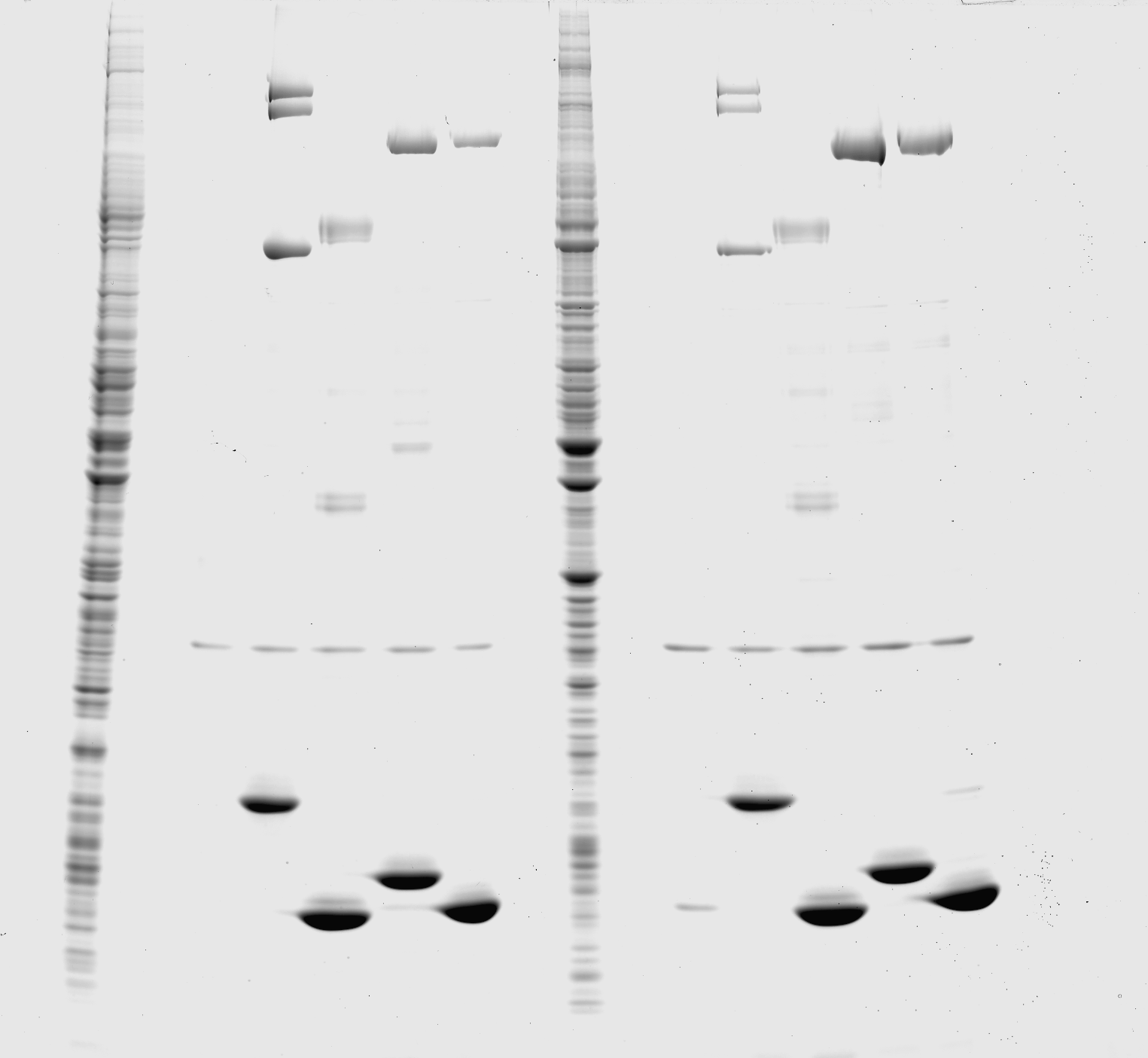

Supplement: Supplementary file 6 — Source data Fig. 6 [file 44318_2024_81_MOESM6_ESM.zip › Fig6D/Figure6D_Nups_pulldown.tif]

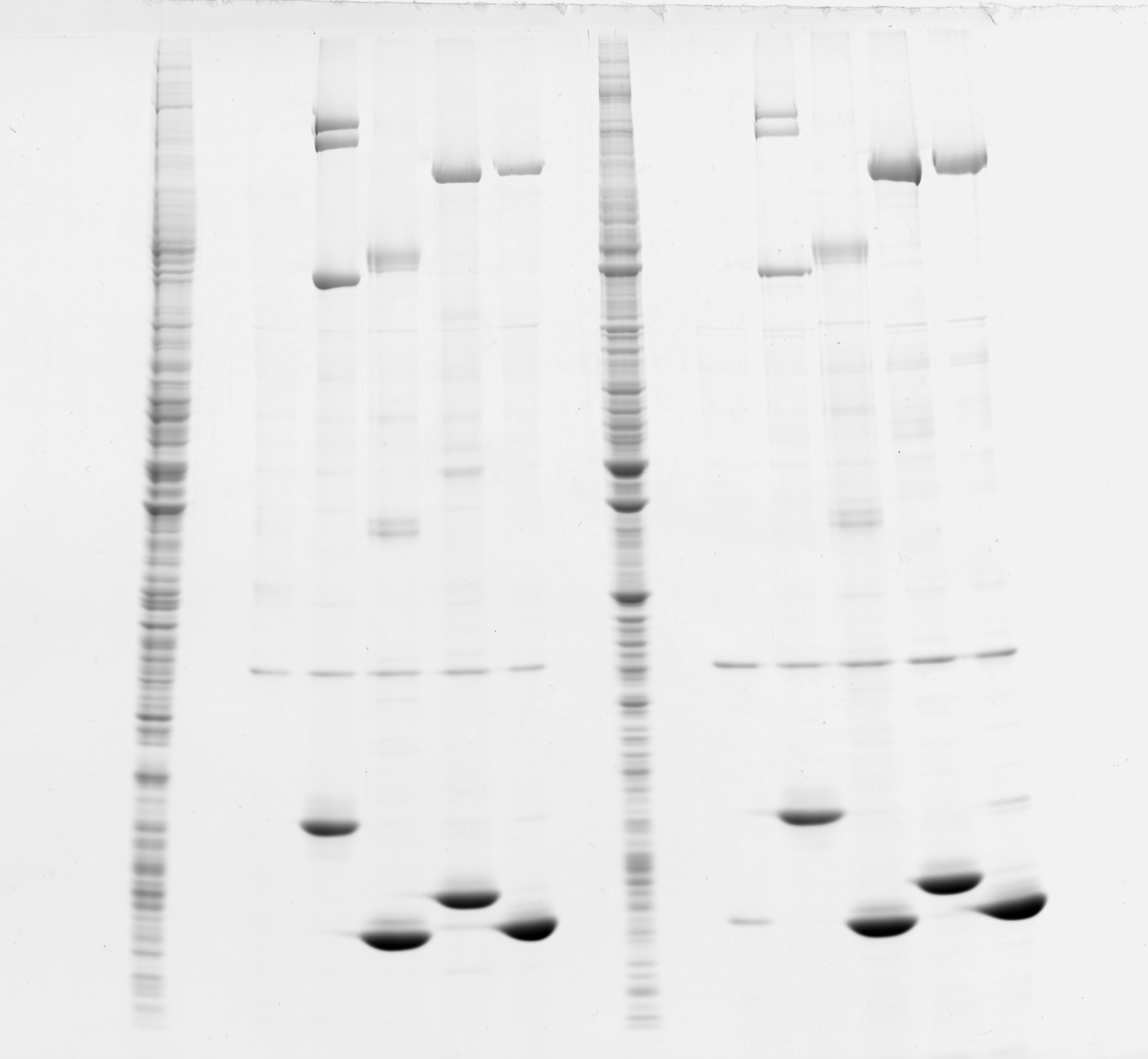

Supplement: Supplementary file 6 — Source data Fig. 6 [file 44318_2024_81_MOESM6_ESM.zip › Fig6D/Figure6D_Nups_pulldown_raw.tif]

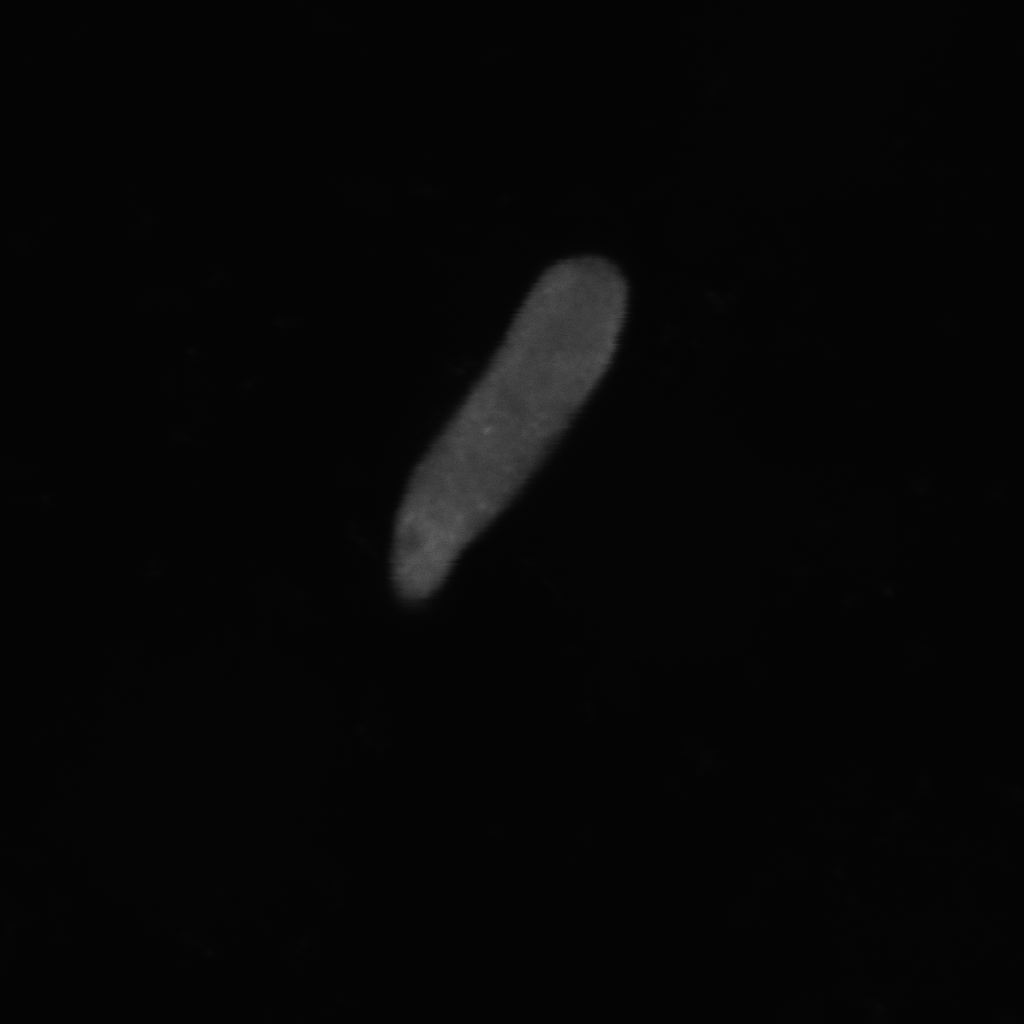

Supplement: Supplementary file 7 — Source data Fig. 7 [file 44318_2024_81_MOESM7_ESM.zip › Fig7ABC/Active_import&Passive_exclusion/BAPTA_IBB-MBP-GFP-MBP_mCherry.tif]

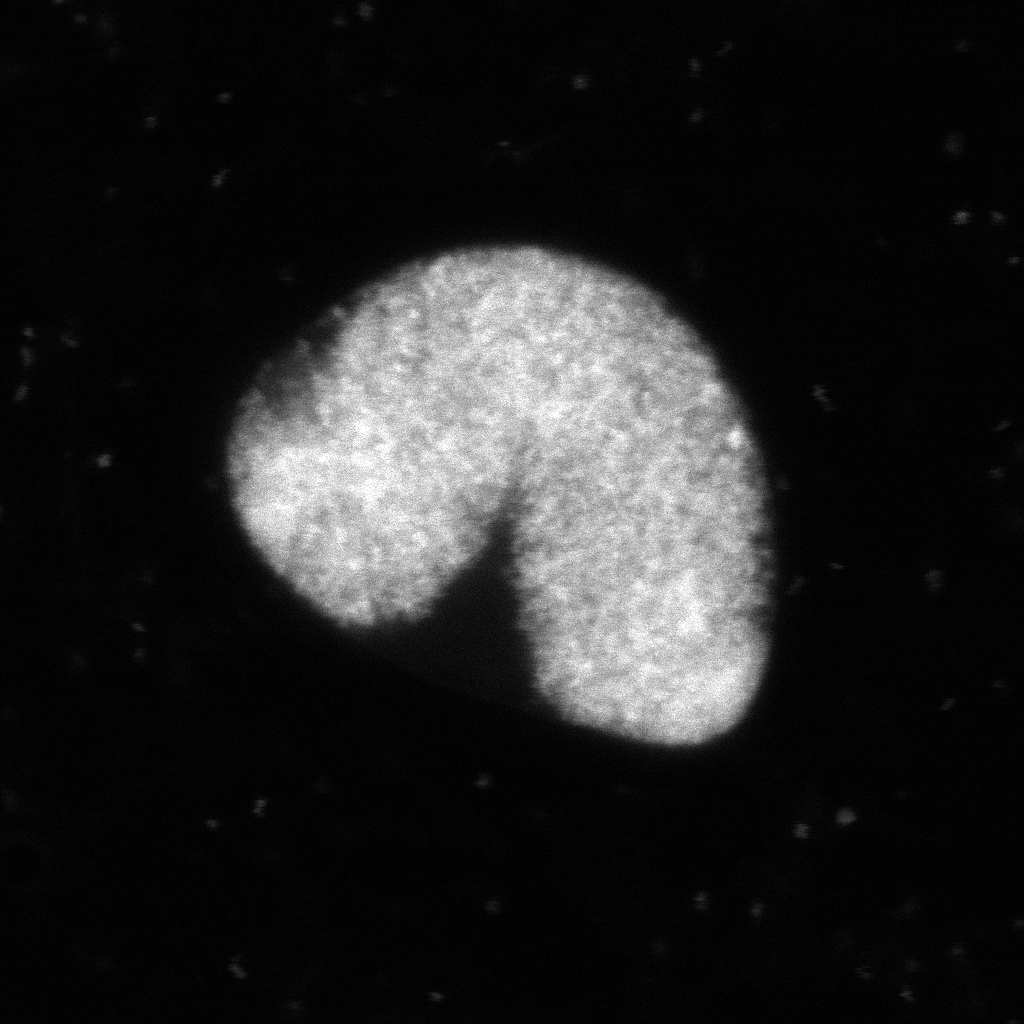

Supplement: Supplementary file 7 — Source data Fig. 7 [file 44318_2024_81_MOESM7_ESM.zip › Fig7ABC/Active_import&Passive_exclusion/Buffer_IBB-MBP-GFP-MBP_mCherry.tif]

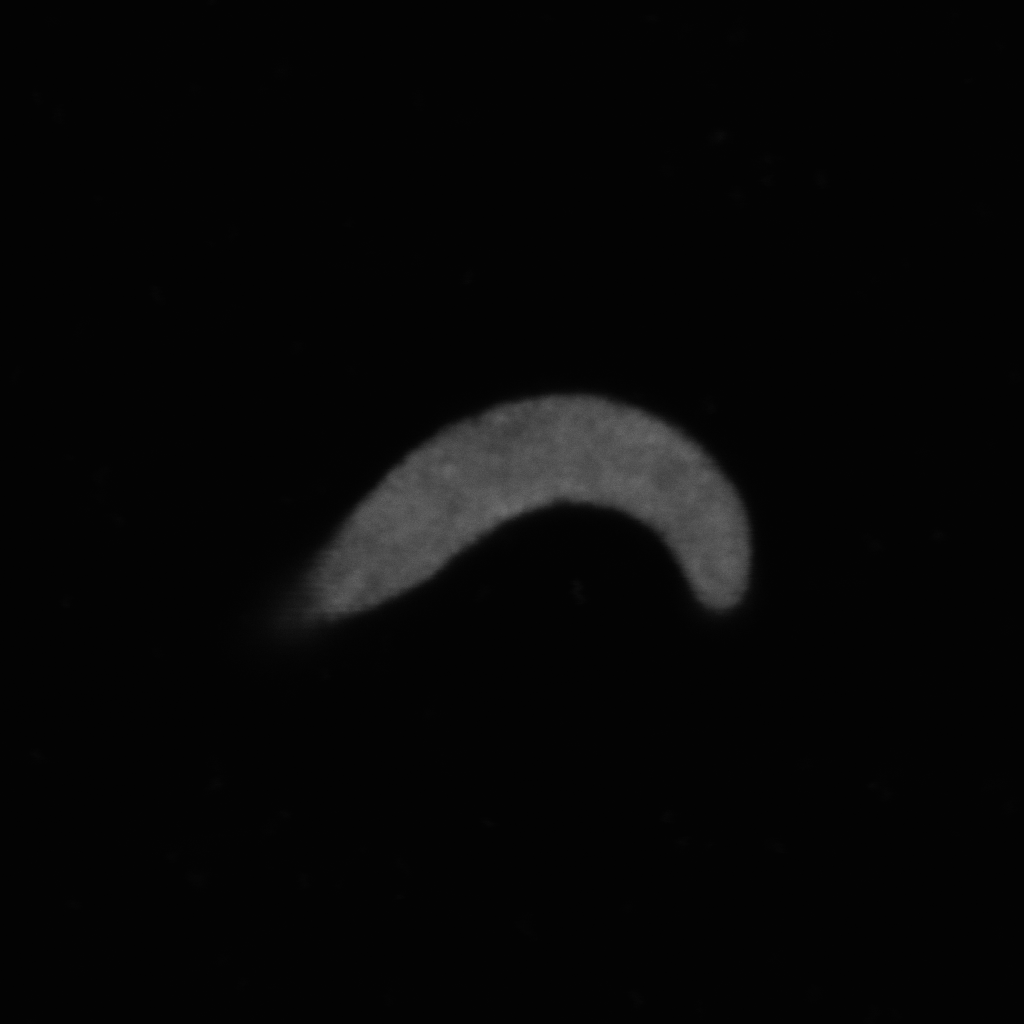

Supplement: Supplementary file 7 — Source data Fig. 7 [file 44318_2024_81_MOESM7_ESM.zip › Fig7ABC/Active_import&Passive_exclusion/Importinb_IBB-MBP-GFP-MBP_mCherry.tif]

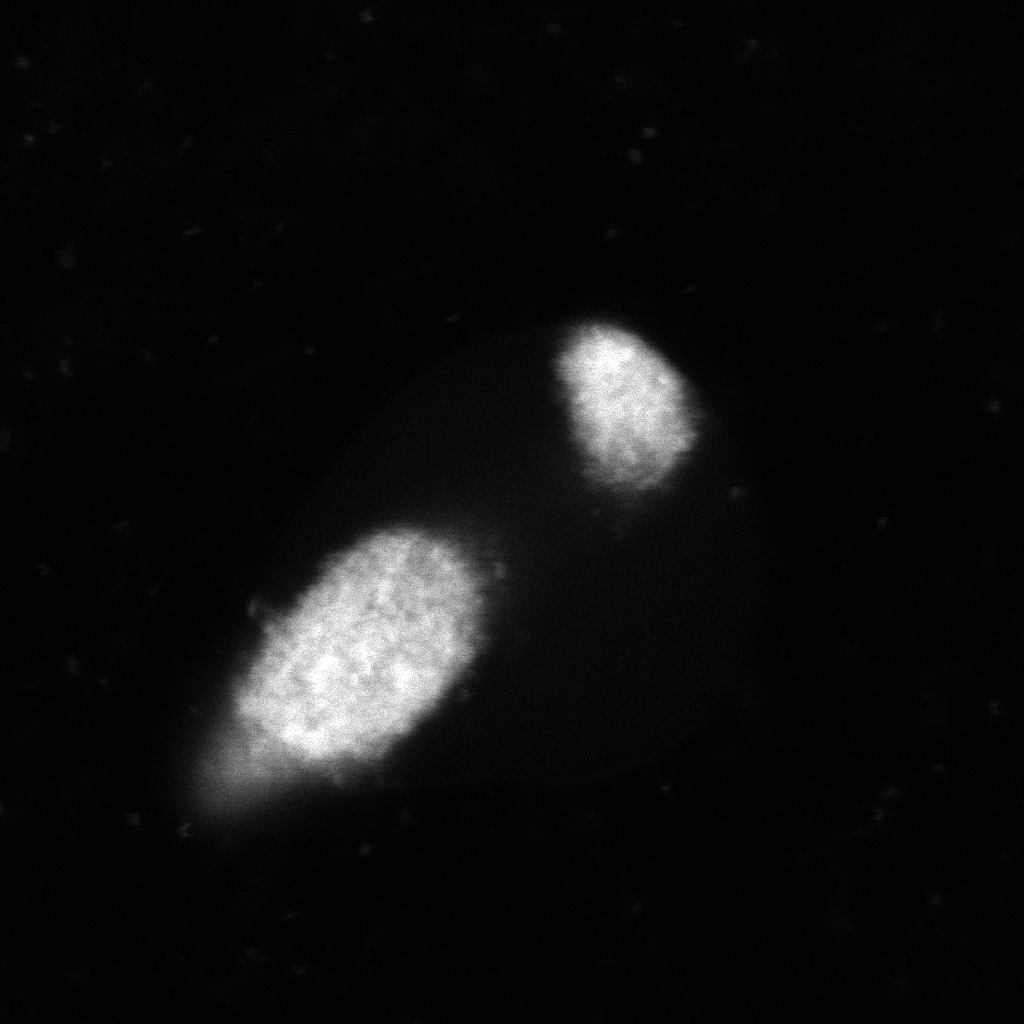

Supplement: Supplementary file 7 — Source data Fig. 7 [file 44318_2024_81_MOESM7_ESM.zip › Fig7ABC/Active_import&Passive_exclusion/NoninhibitoryNb_IBB-MBP-GFP-MBP_mCherry.tif]

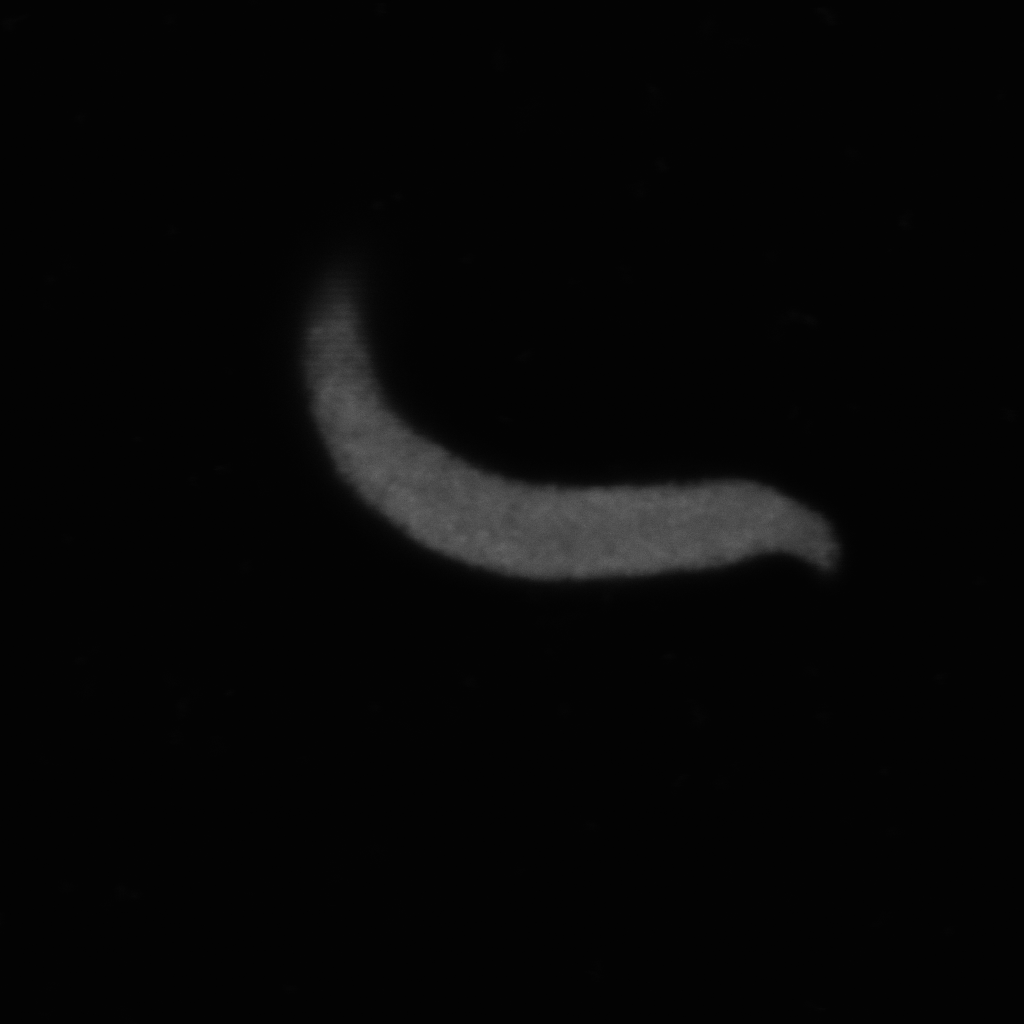

Supplement: Supplementary file 7 — Source data Fig. 7 [file 44318_2024_81_MOESM7_ESM.zip › Fig7ABC/Active_import&Passive_exclusion/WGA_IBB-MBP-GFP-MBP_mCherry.tif]

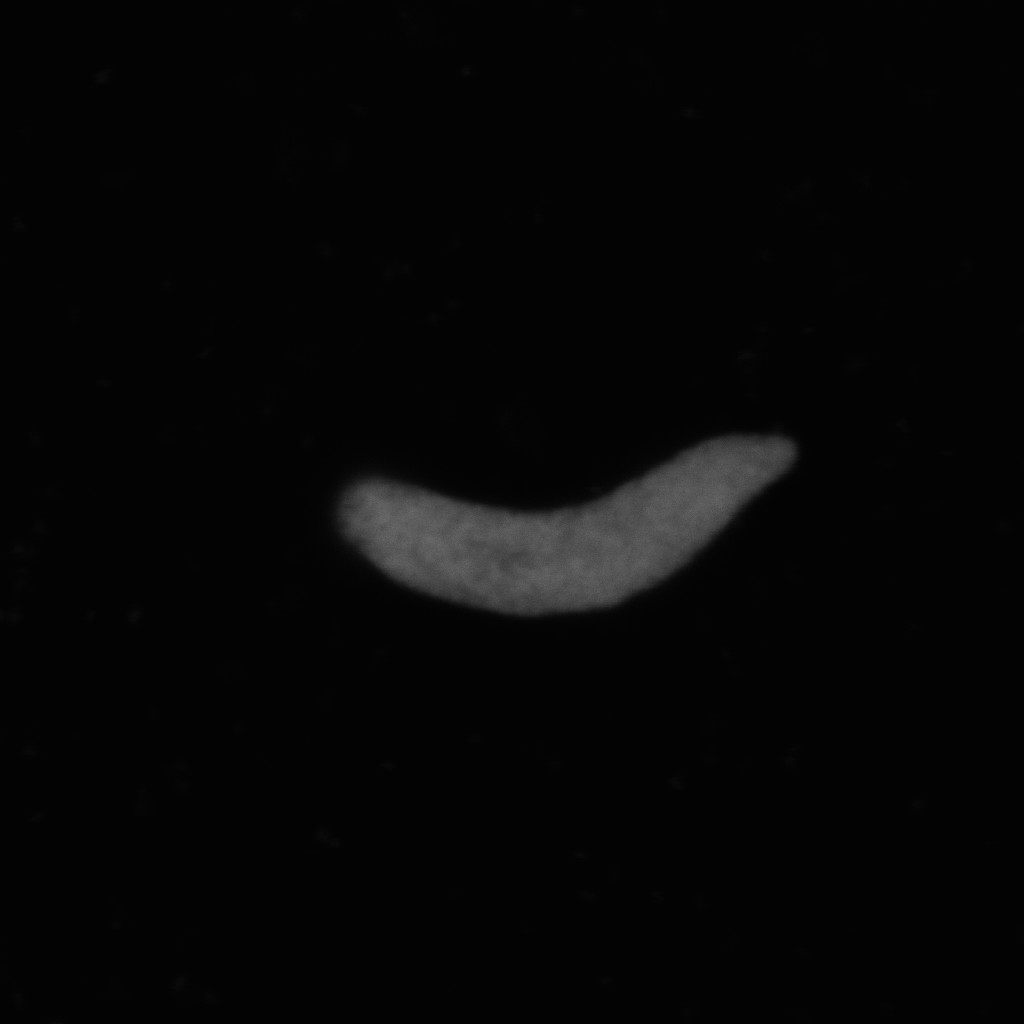

Supplement: Supplementary file 7 — Source data Fig. 7 [file 44318_2024_81_MOESM7_ESM.zip › Fig7ABC/Active_import&Passive_exclusion/xhNup155-Nb2i_IBB-MBP-GFP-MBP_mCherry.tif]

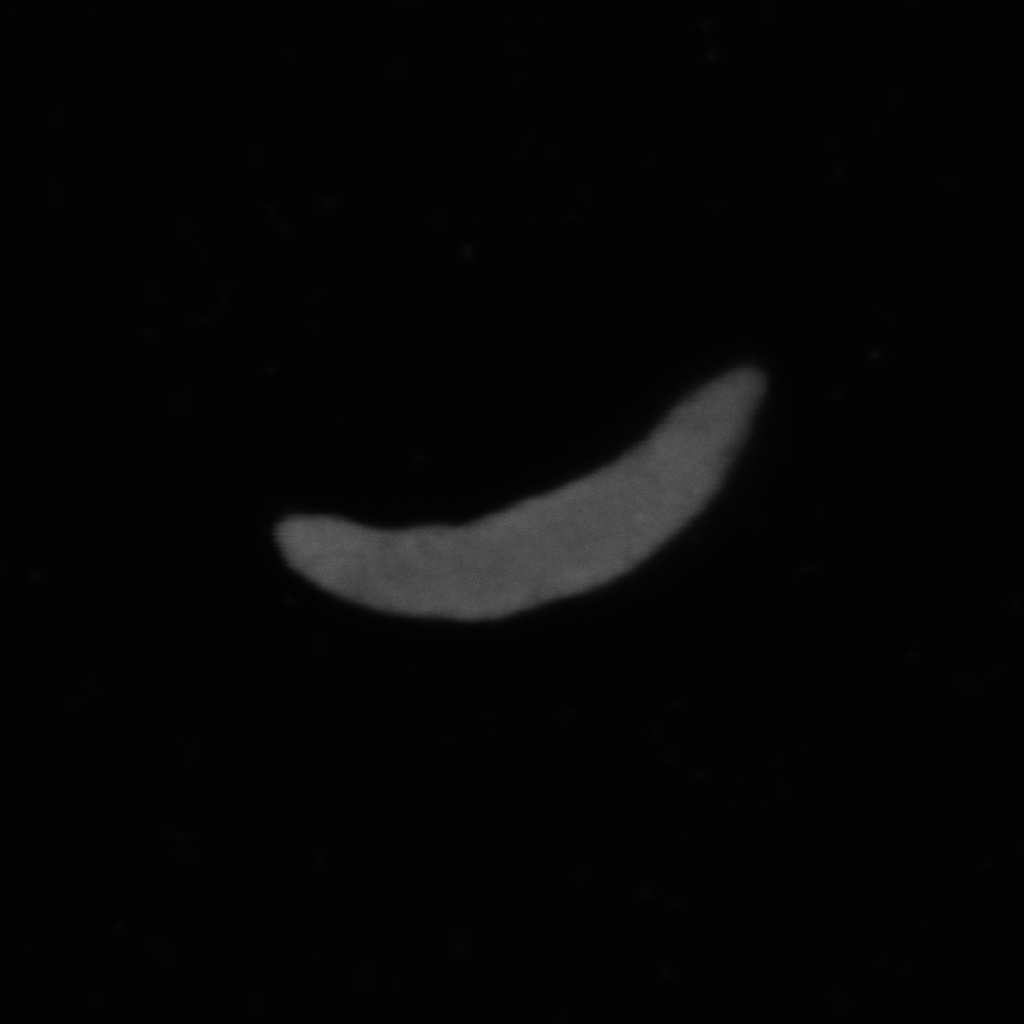

Supplement: Supplementary file 7 — Source data Fig. 7 [file 44318_2024_81_MOESM7_ESM.zip › Fig7ABC/Active_import&Passive_exclusion/xhNup155-Nb3i_IBB-MBP-GFP-MBP_mCherry.tif]

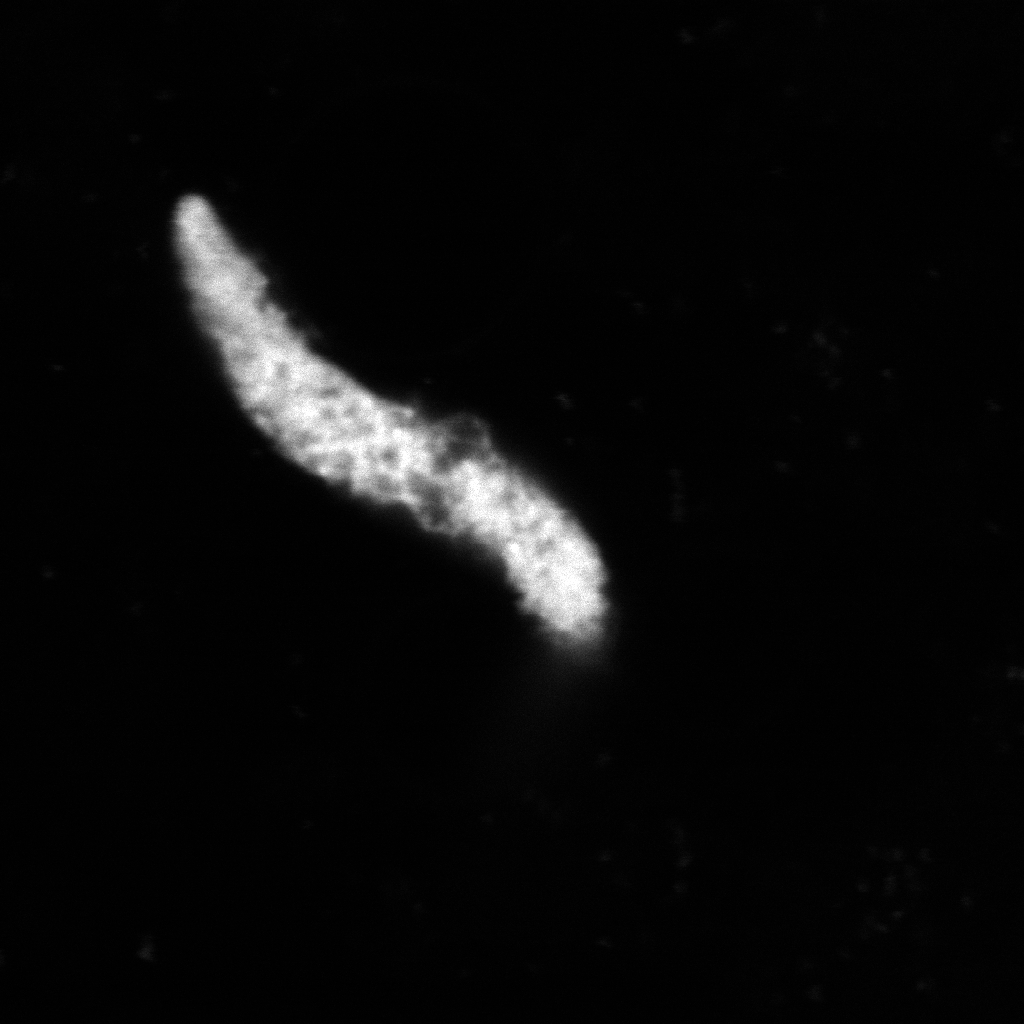

Supplement: Supplementary file 7 — Source data Fig. 7 [file 44318_2024_81_MOESM7_ESM.zip › Fig7ABC/Active_import&Passive_exclusion/xhNup93-Nb3i_IBB-MBP-GFP-MBP_mCherry-example2.tif]

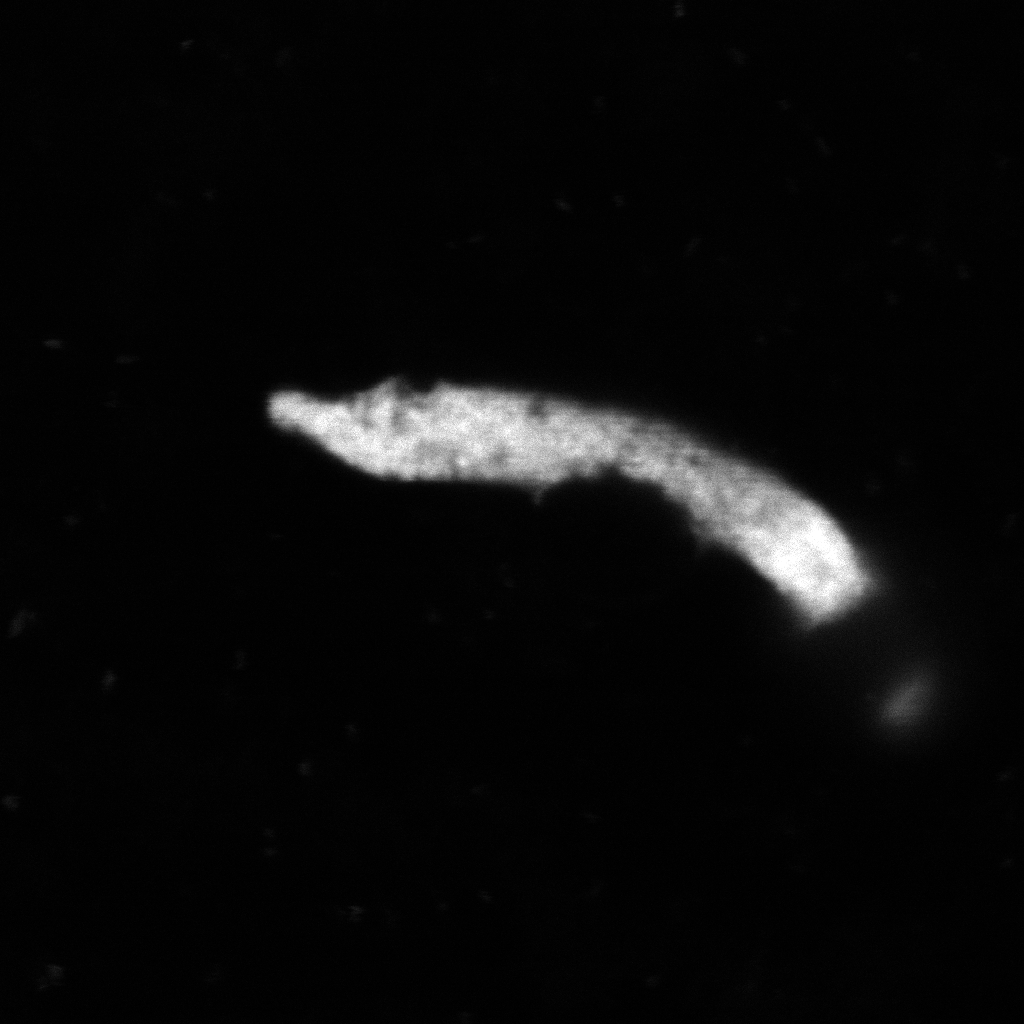

Supplement: Supplementary file 7 — Source data Fig. 7 [file 44318_2024_81_MOESM7_ESM.zip › Fig7ABC/Active_import&Passive_exclusion/xhNup93-Nb3i_IBB-MBP-GFP-MBP_mCherry-example3.tif]

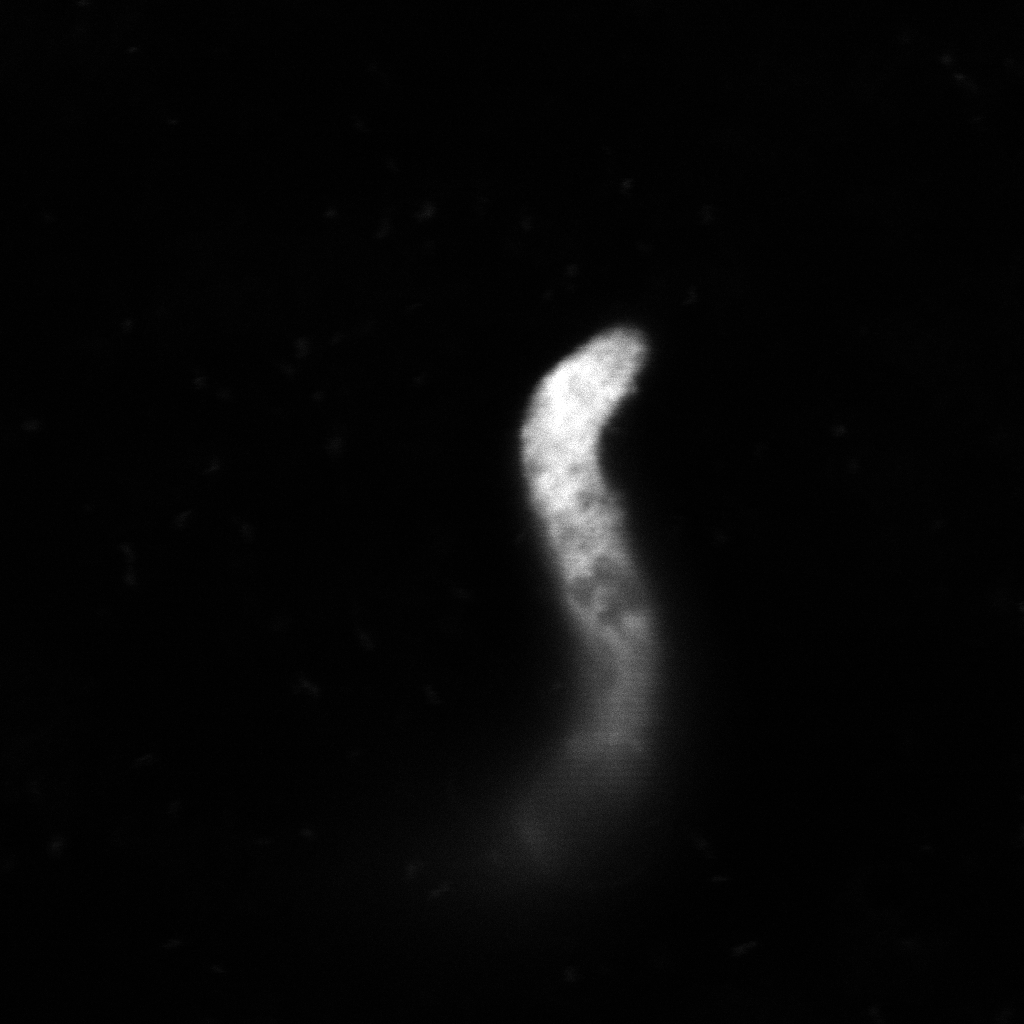

Supplement: Supplementary file 7 — Source data Fig. 7 [file 44318_2024_81_MOESM7_ESM.zip › Fig7ABC/Active_import&Passive_exclusion/xhNup93-Nb3i_IBB-MBP-GFP-MBP_mCherry-example4.tif]

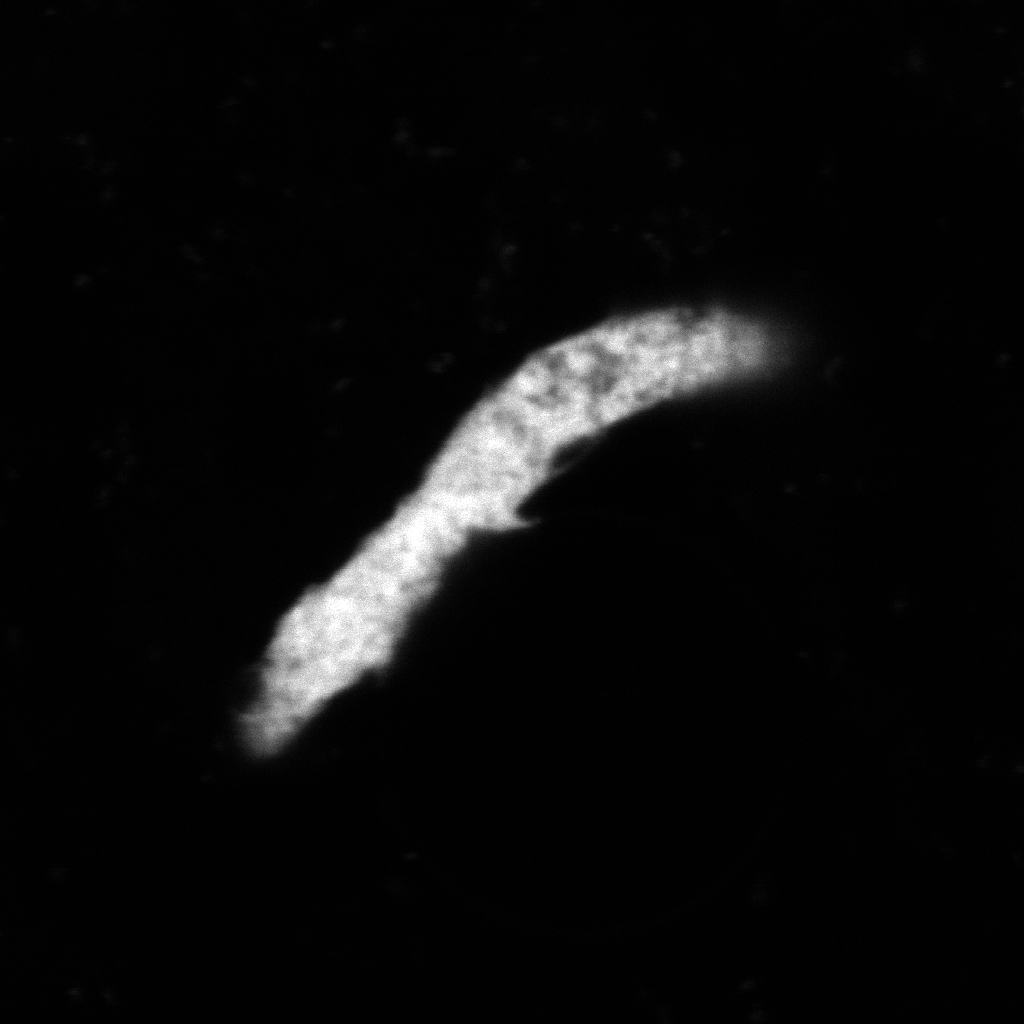

Supplement: Supplementary file 7 — Source data Fig. 7 [file 44318_2024_81_MOESM7_ESM.zip › Fig7ABC/Active_import&Passive_exclusion/xhNup93-Nb3i_IBB-MBP-GFP-MBP_mCherry-figure.tif]

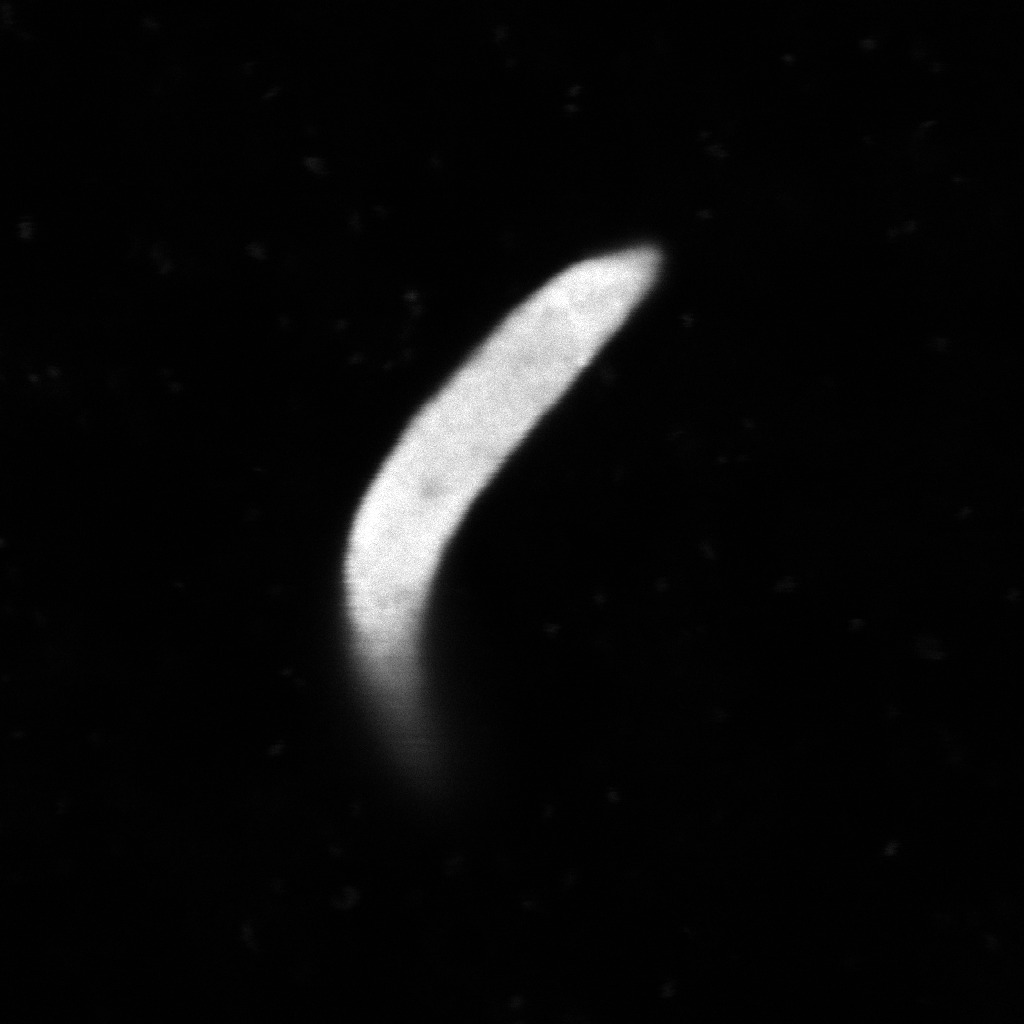

Supplement: Supplementary file 7 — Source data Fig. 7 [file 44318_2024_81_MOESM7_ESM.zip › Fig7ABC/Active_import&Passive_exclusion/xhNup98-Nb2i_IBB-MBP-GFP-MBP_mCherry.tif]

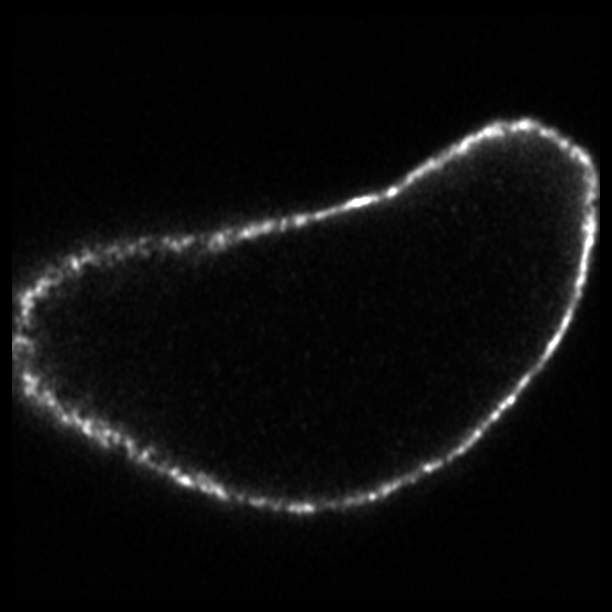

Supplement: Supplementary file 8 — Source data Fig. 8 [file 44318_2024_81_MOESM8_ESM.zip › Fig8A/Fig8A_Nup358-Nb1t/Fig8A_xNup358-Nb1t_Buffer.tif]

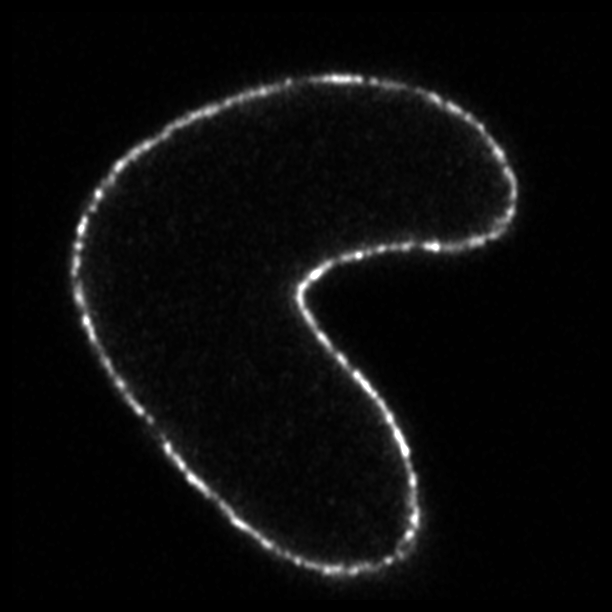

Supplement: Supplementary file 8 — Source data Fig. 8 [file 44318_2024_81_MOESM8_ESM.zip › Fig8A/Fig8A_Nup358-Nb1t/Fig8A_xNup358-Nb1t_NoninhibitoryNb.tif]

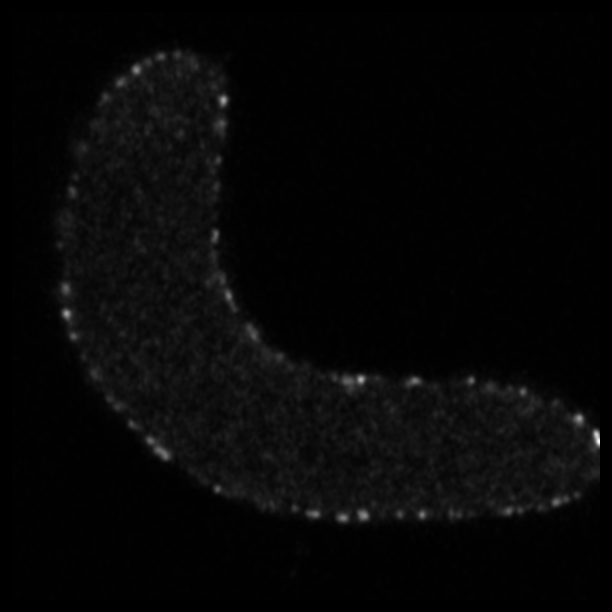

Supplement: Supplementary file 8 — Source data Fig. 8 [file 44318_2024_81_MOESM8_ESM.zip › Fig8A/Fig8A_Nup358-Nb1t/Fig8A_xNup358-Nb1t_xhNup155-Nb2i.tif]

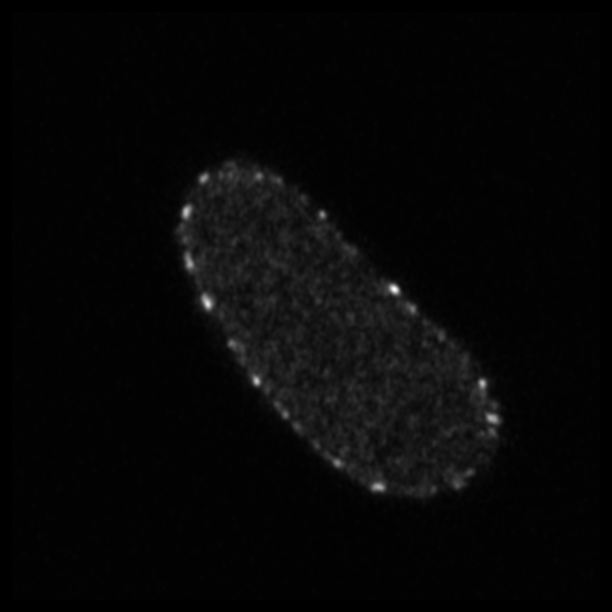

Supplement: Supplementary file 8 — Source data Fig. 8 [file 44318_2024_81_MOESM8_ESM.zip › Fig8A/Fig8A_Nup358-Nb1t/Fig8A_xNup358-Nb1t_xhNup155-Nb3i.tif]

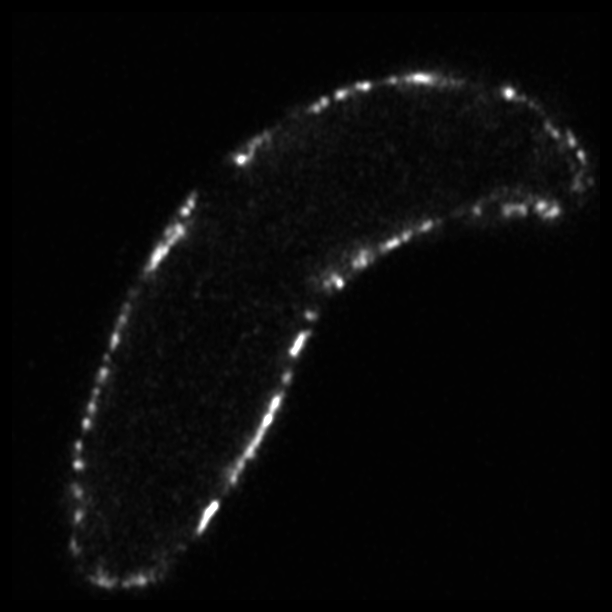

Supplement: Supplementary file 8 — Source data Fig. 8 [file 44318_2024_81_MOESM8_ESM.zip › Fig8A/Fig8A_Nup358-Nb1t/Fig8A_xNup358-Nb1t_xhNup93-Nb4i.tif]

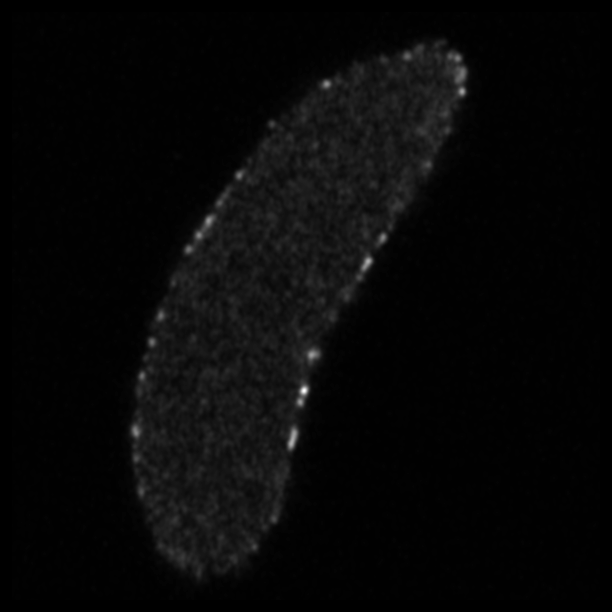

Supplement: Supplementary file 8 — Source data Fig. 8 [file 44318_2024_81_MOESM8_ESM.zip › Fig8A/Fig8A_Nup358-Nb1t/Fig8A_xNup358-Nb1t_xhNup98-Nb2i.tif]

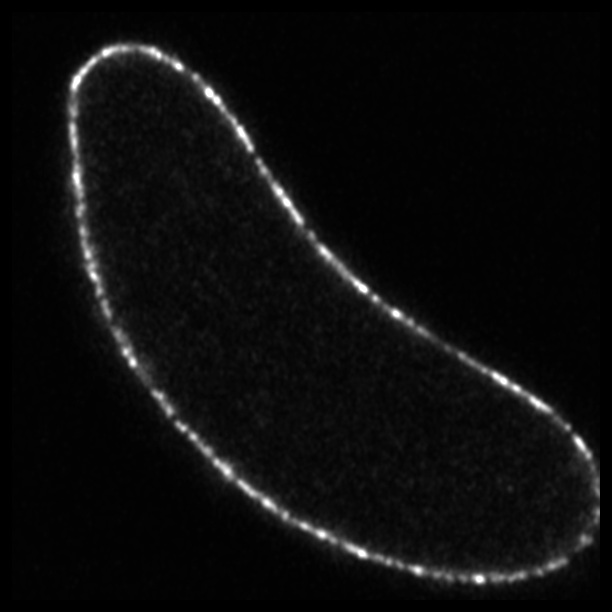

Supplement: Supplementary file 8 — Source data Fig. 8 [file 44318_2024_81_MOESM8_ESM.zip › Fig8A/Fig8A_xNup155-Nb1t/Fig8A_xNup155_Nb1t_Buffer.tif]

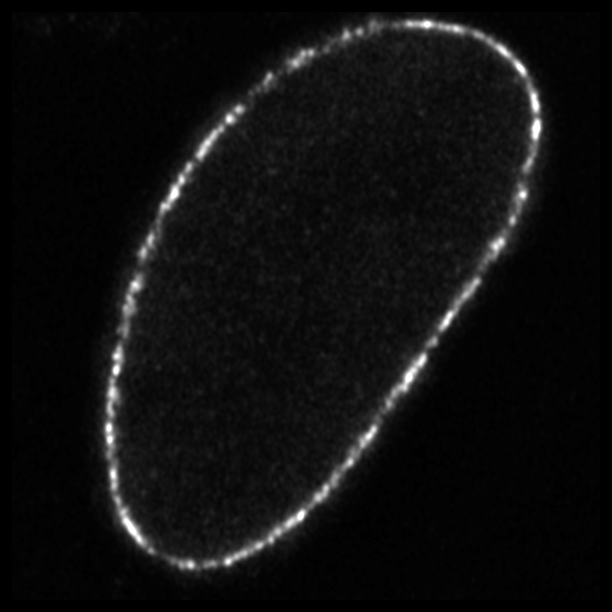

Supplement: Supplementary file 8 — Source data Fig. 8 [file 44318_2024_81_MOESM8_ESM.zip › Fig8A/Fig8A_xNup155-Nb1t/Fig8A_xNup155_Nb1t_NoninhibitoryNb.tif]
