# Supplementary figures and images for: A checkpoint function for Nup98 in nuclear pore formation suggested by novel inhibitory nanobodies (part 2 of 2)
Source: EMBO J. 2024 Apr 22;43(11):6. doi: 10.1038/s44318-024-00081-w (PMC11148069; doi:10.1038/s44318-024-00081-w)

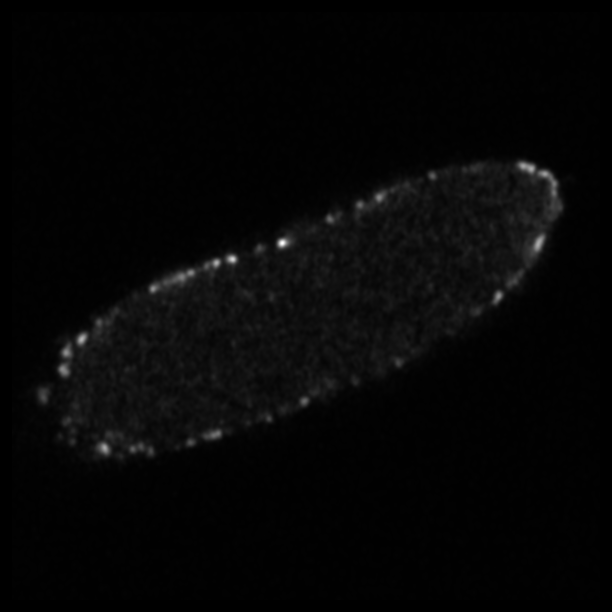

Supplement: Supplementary file 8 — Source data Fig. 8 [file 44318_2024_81_MOESM8_ESM.zip › Fig8A/Fig8A_xNup155-Nb1t/Fig8A_xNup155_Nb1t_xhNup155-Nb2i.tif]

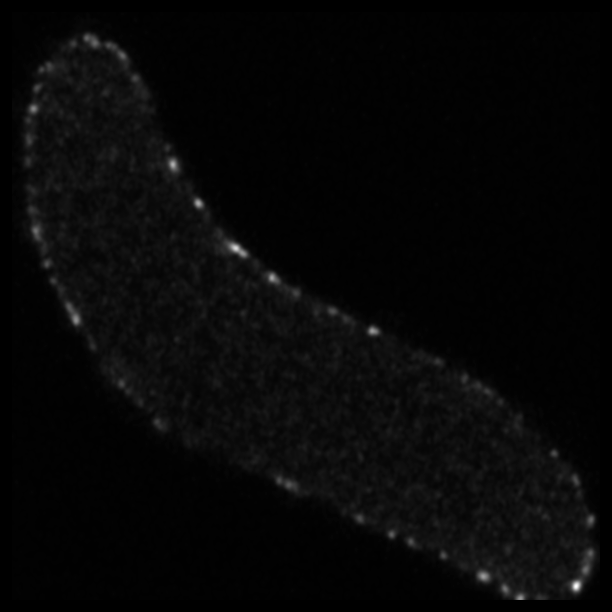

Supplement: Supplementary file 8 — Source data Fig. 8 [file 44318_2024_81_MOESM8_ESM.zip › Fig8A/Fig8A_xNup155-Nb1t/Fig8A_xNup155_Nb1t_xhNup155-Nb3i.tif]

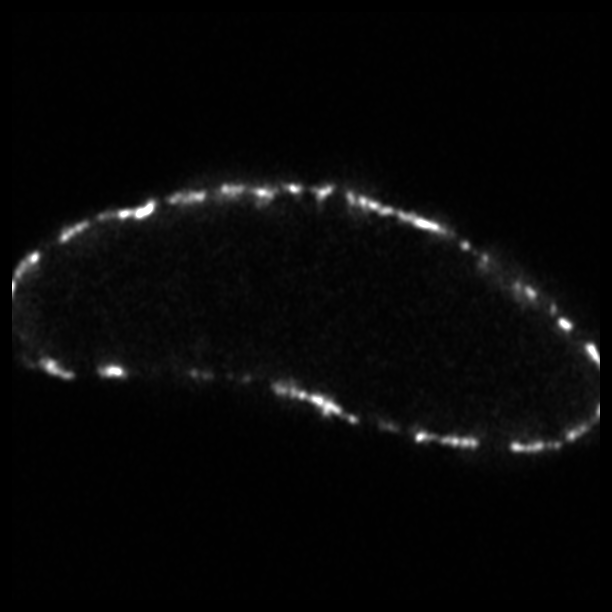

Supplement: Supplementary file 8 — Source data Fig. 8 [file 44318_2024_81_MOESM8_ESM.zip › Fig8A/Fig8A_xNup155-Nb1t/Fig8A_xNup155_Nb1t_xhNup93-Nb4i.tif]

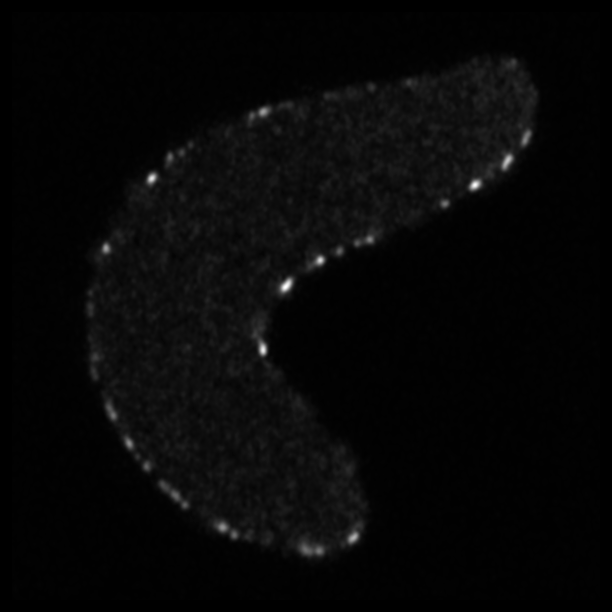

Supplement: Supplementary file 8 — Source data Fig. 8 [file 44318_2024_81_MOESM8_ESM.zip › Fig8A/Fig8A_xNup155-Nb1t/Fig8A_xNup155_Nb1t_xhNup98-Nb2i.tif]

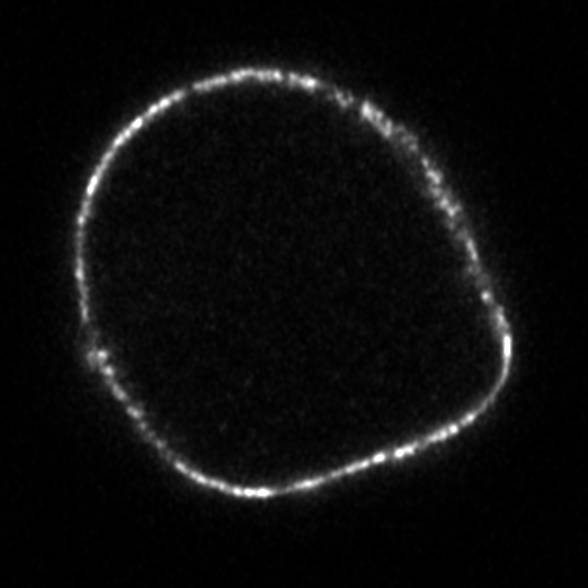

Supplement: Supplementary file 8 — Source data Fig. 8 [file 44318_2024_81_MOESM8_ESM.zip › Fig8A/Fig8A_xNup93-Nb1t_xhNup35-Nb1t/Fig8A_xNup93_Nb1t-xhNup35_Nb1t_Buffer.tif]

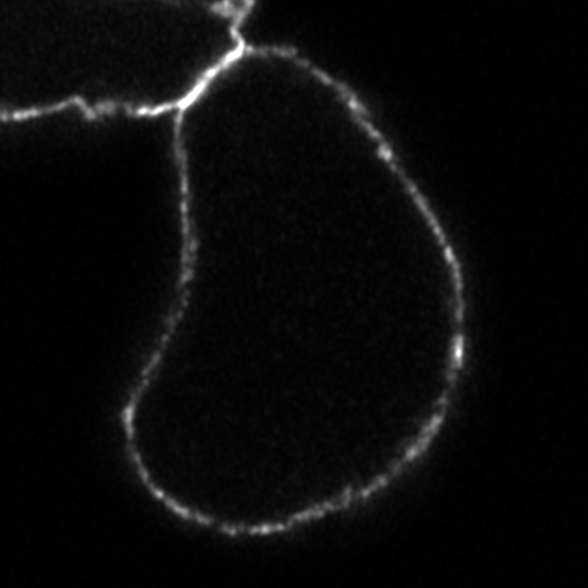

Supplement: Supplementary file 8 — Source data Fig. 8 [file 44318_2024_81_MOESM8_ESM.zip › Fig8A/Fig8A_xNup93-Nb1t_xhNup35-Nb1t/Fig8A_xNup93_Nb1t-xhNup35_Nb1t_NoninhibitoryNb.tif]

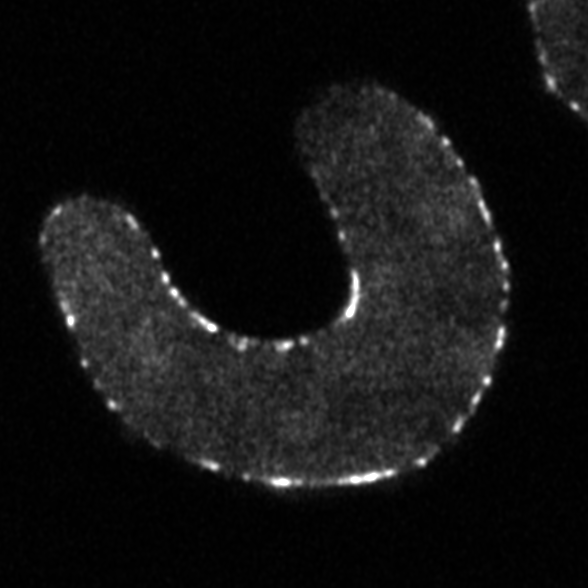

Supplement: Supplementary file 8 — Source data Fig. 8 [file 44318_2024_81_MOESM8_ESM.zip › Fig8A/Fig8A_xNup93-Nb1t_xhNup35-Nb1t/Fig8A_xNup93_Nb1t-xhNup35_Nb1t_xhNup155_Nb2i.tif]

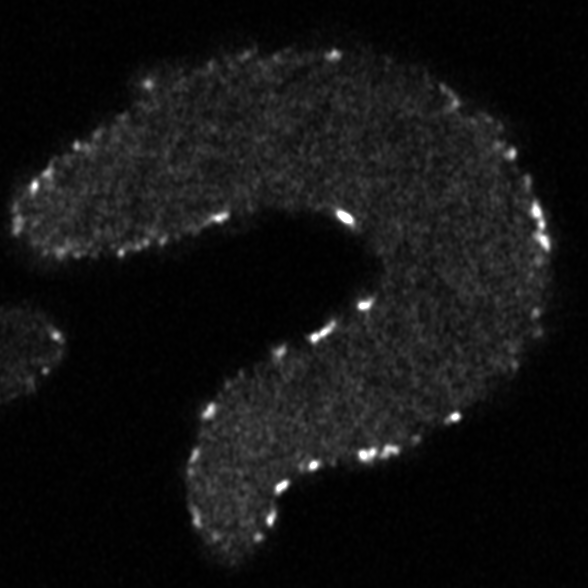

Supplement: Supplementary file 8 — Source data Fig. 8 [file 44318_2024_81_MOESM8_ESM.zip › Fig8A/Fig8A_xNup93-Nb1t_xhNup35-Nb1t/Fig8A_xNup93_Nb1t-xhNup35_Nb1t_xhNup155_Nb3i.tif]

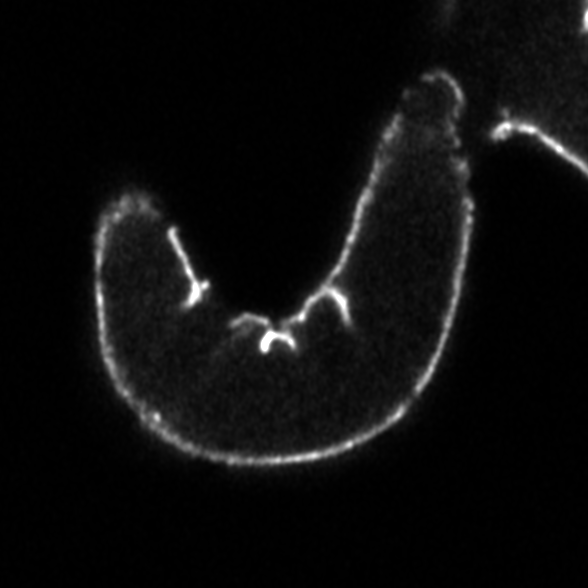

Supplement: Supplementary file 8 — Source data Fig. 8 [file 44318_2024_81_MOESM8_ESM.zip › Fig8A/Fig8A_xNup93-Nb1t_xhNup35-Nb1t/Fig8A_xNup93_Nb1t-xhNup35_Nb1t_xhNup93_Nb4i.tif]

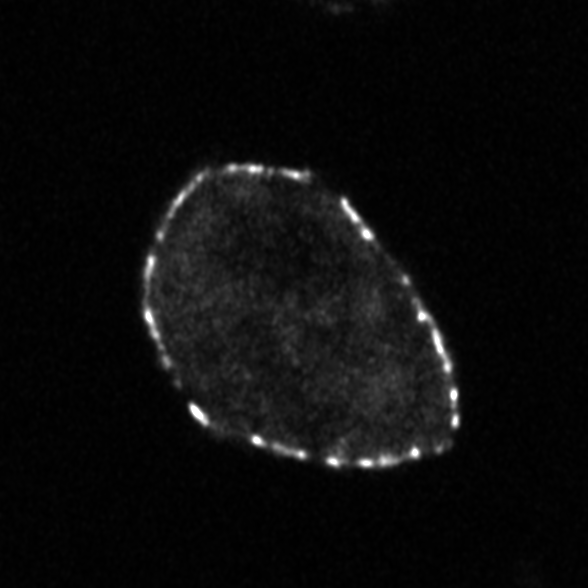

Supplement: Supplementary file 8 — Source data Fig. 8 [file 44318_2024_81_MOESM8_ESM.zip › Fig8A/Fig8A_xNup93-Nb1t_xhNup35-Nb1t/Fig8A_xNup93_Nb1t-xhNup35_Nb1t_xhNup98_Nb2i.tif]

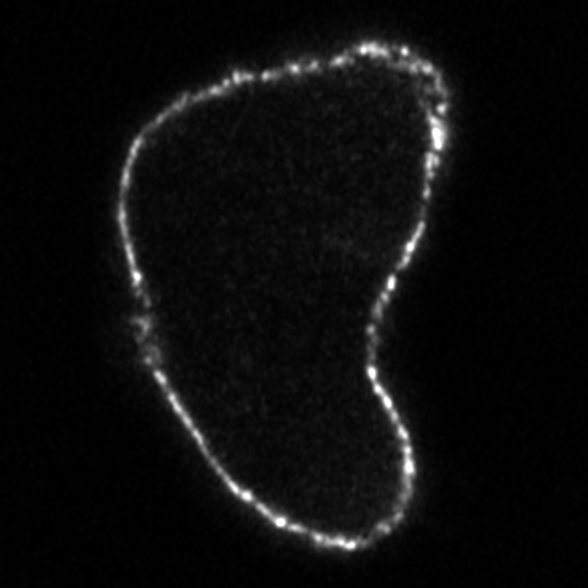

Supplement: Supplementary file 8 — Source data Fig. 8 [file 44318_2024_81_MOESM8_ESM.zip › Fig8A/Fig8A_xNup98-Nb1t/Fig8A_xhNup98-Nb1t_Buffer.tif]

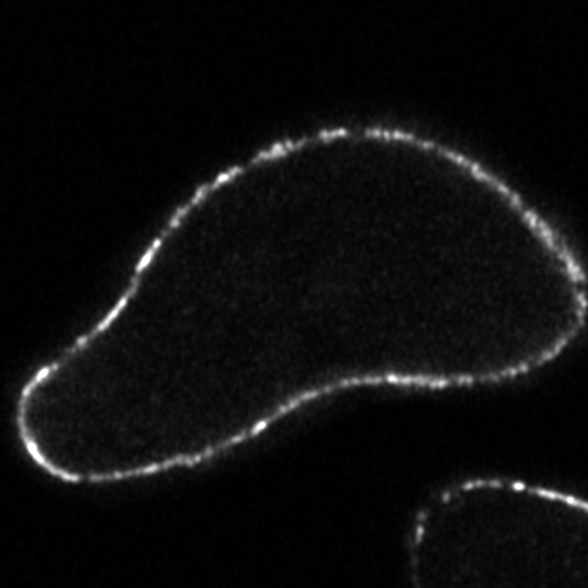

Supplement: Supplementary file 8 — Source data Fig. 8 [file 44318_2024_81_MOESM8_ESM.zip › Fig8A/Fig8A_xNup98-Nb1t/Fig8A_xhNup98-Nb1t_NoninhibitoryNb.tif]

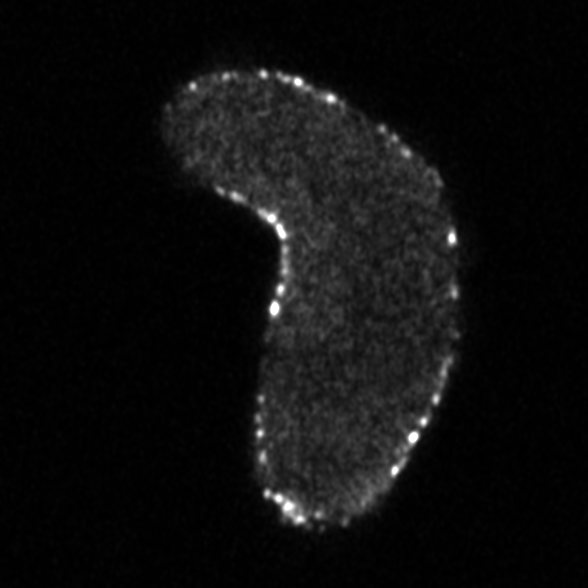

Supplement: Supplementary file 8 — Source data Fig. 8 [file 44318_2024_81_MOESM8_ESM.zip › Fig8A/Fig8A_xNup98-Nb1t/Fig8A_xhNup98-Nb1t_xhNup155_Nb2i.tif]

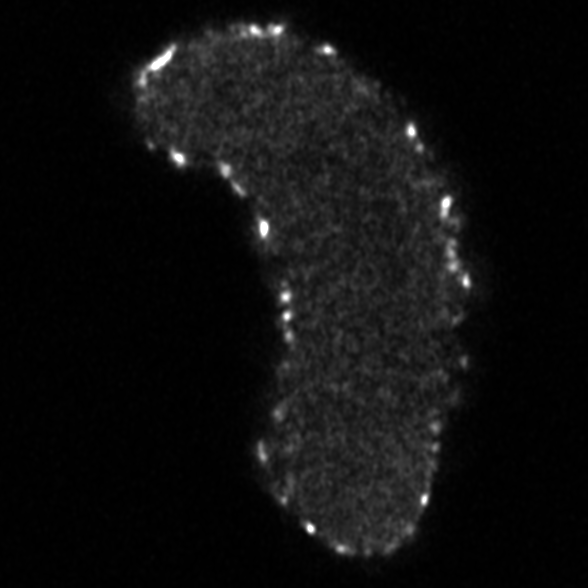

Supplement: Supplementary file 8 — Source data Fig. 8 [file 44318_2024_81_MOESM8_ESM.zip › Fig8A/Fig8A_xNup98-Nb1t/Fig8A_xhNup98-Nb1t_xhNup155_Nb3i.tif]

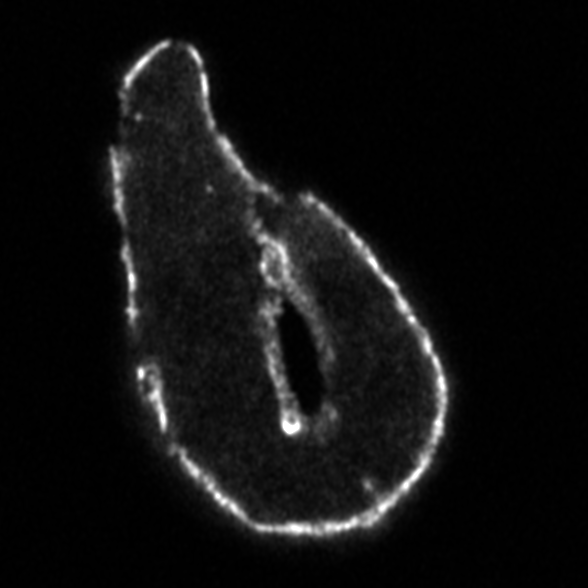

Supplement: Supplementary file 8 — Source data Fig. 8 [file 44318_2024_81_MOESM8_ESM.zip › Fig8A/Fig8A_xNup98-Nb1t/Fig8A_xhNup98-Nb1t_xhNup93_Nb4i.tif]

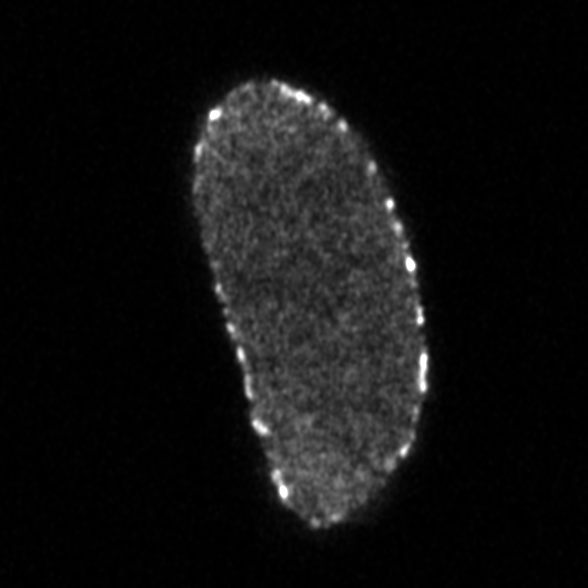

Supplement: Supplementary file 8 — Source data Fig. 8 [file 44318_2024_81_MOESM8_ESM.zip › Fig8A/Fig8A_xNup98-Nb1t/Fig8A_xhNup98-Nb1t_xhNup98_Nb2i.tif]

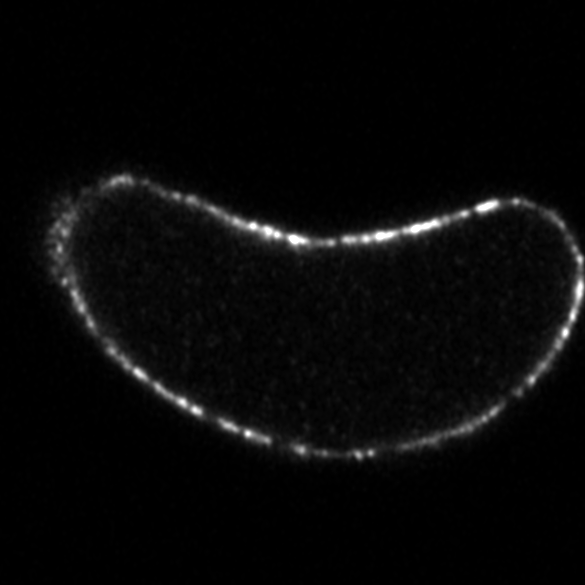

Supplement: Supplementary file 8 — Source data Fig. 8 [file 44318_2024_81_MOESM8_ESM.zip › Fig8A/Fig8A_xY-Nb1t_xNup62cmplx-Nb1t_xhNup214-Nb1t/Fig8A_xYcmplx_Nb1t_xNup62cmplx_Nb1t-xhNup214cmplx_Nb1t_Buffer.tif]

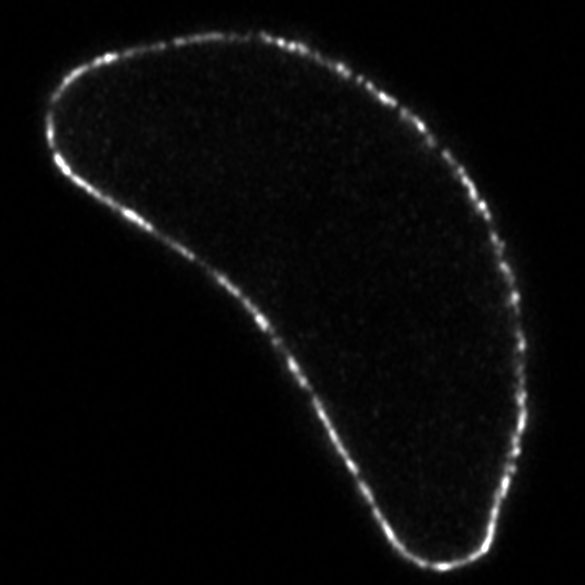

Supplement: Supplementary file 8 — Source data Fig. 8 [file 44318_2024_81_MOESM8_ESM.zip › Fig8A/Fig8A_xY-Nb1t_xNup62cmplx-Nb1t_xhNup214-Nb1t/Fig8A_xYcmplx_Nb1t_xNup62cmplx_Nb1t-xhNup214cmplx_Nb1t_NoninhibitoryNb.tif]

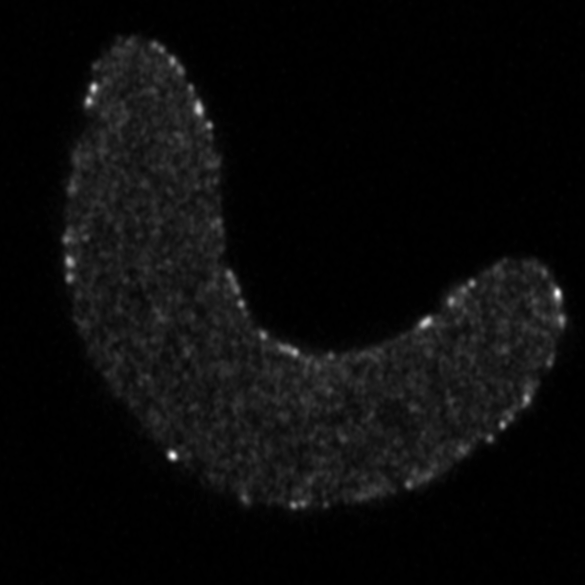

Supplement: Supplementary file 8 — Source data Fig. 8 [file 44318_2024_81_MOESM8_ESM.zip › Fig8A/Fig8A_xY-Nb1t_xNup62cmplx-Nb1t_xhNup214-Nb1t/Fig8A_xYcmplx_Nb1t_xNup62cmplx_Nb1t-xhNup214cmplx_Nb1t_xhNup155_Nb2i.tif]

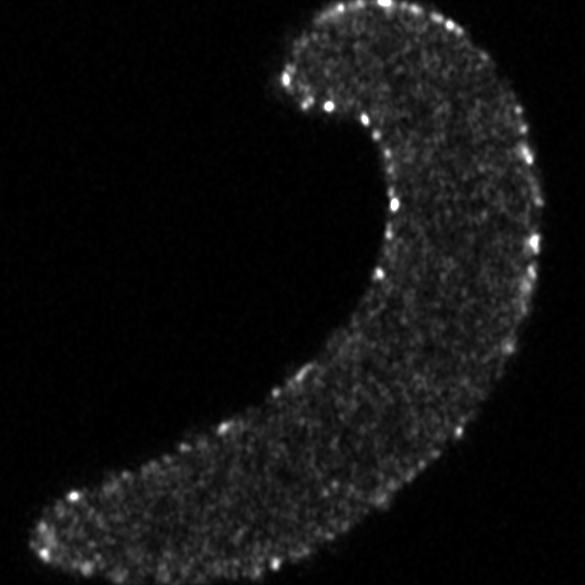

Supplement: Supplementary file 8 — Source data Fig. 8 [file 44318_2024_81_MOESM8_ESM.zip › Fig8A/Fig8A_xY-Nb1t_xNup62cmplx-Nb1t_xhNup214-Nb1t/Fig8A_xYcmplx_Nb1t_xNup62cmplx_Nb1t-xhNup214cmplx_Nb1t_xhNup155_Nb3i.tif]

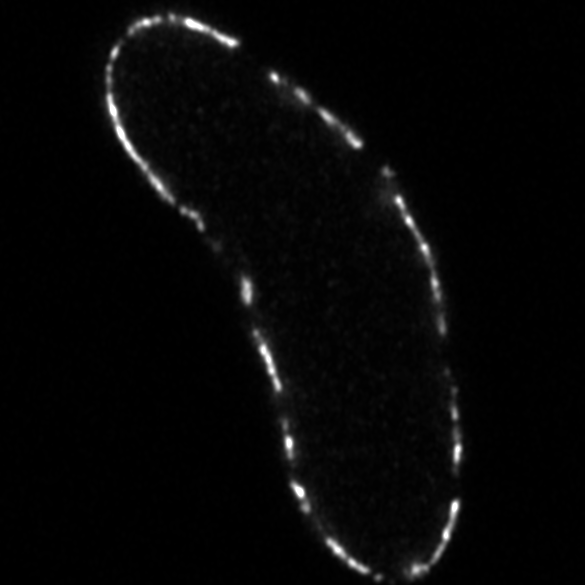

Supplement: Supplementary file 8 — Source data Fig. 8 [file 44318_2024_81_MOESM8_ESM.zip › Fig8A/Fig8A_xY-Nb1t_xNup62cmplx-Nb1t_xhNup214-Nb1t/Fig8A_xYcmplx_Nb1t_xNup62cmplx_Nb1t-xhNup214cmplx_Nb1t_xhNup93_Nb4i.tif]

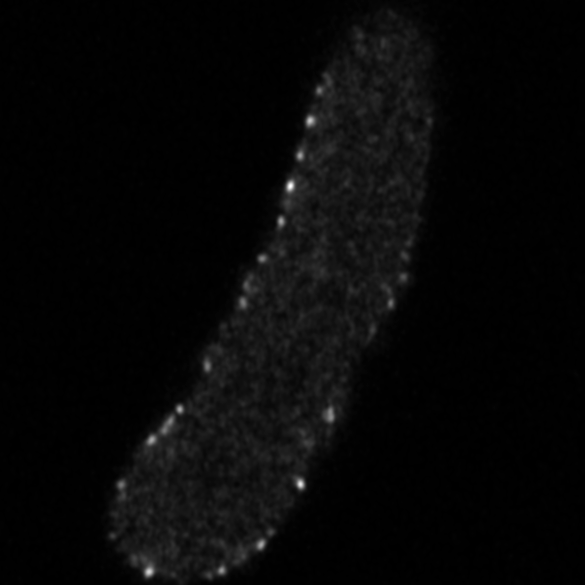

Supplement: Supplementary file 8 — Source data Fig. 8 [file 44318_2024_81_MOESM8_ESM.zip › Fig8A/Fig8A_xY-Nb1t_xNup62cmplx-Nb1t_xhNup214-Nb1t/Fig8A_xYcmplx_Nb1t_xNup62cmplx_Nb1t-xhNup214cmplx_Nb1t_xhNup98_Nb2i.tif]

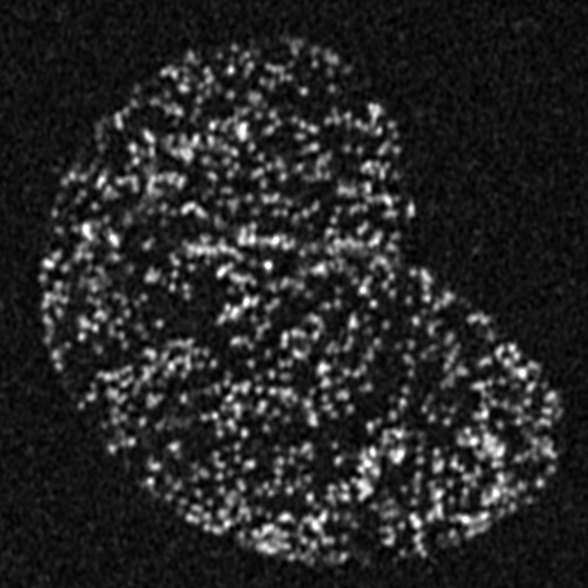

Supplement: Supplementary file 8 — Source data Fig. 8 [file 44318_2024_81_MOESM8_ESM.zip › Fig8B/Fig8B_xNup155-Nb1t_xNup98-Nb1t/Fig8B_xNup155-Nb1t_xNup98-Nb1t_Buffer.tif]

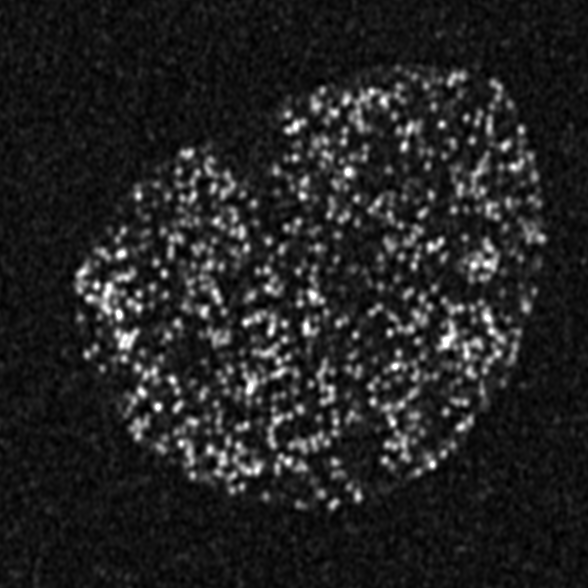

Supplement: Supplementary file 8 — Source data Fig. 8 [file 44318_2024_81_MOESM8_ESM.zip › Fig8B/Fig8B_xNup155-Nb1t_xNup98-Nb1t/Fig8B_xNup155-Nb1t_xNup98-Nb1t_NoninhibitoryNb.tif]

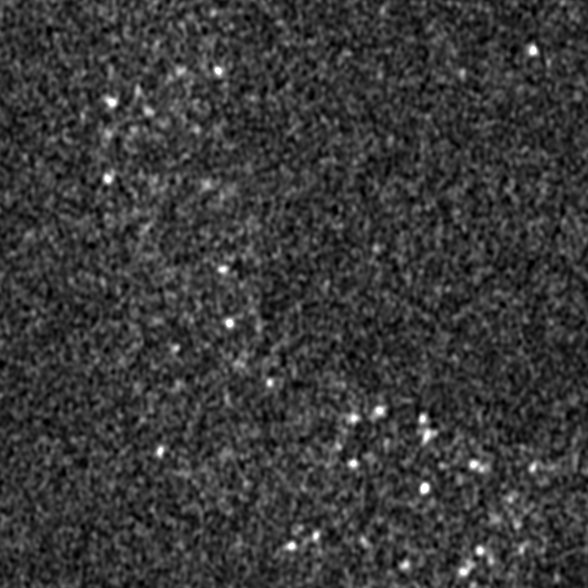

Supplement: Supplementary file 8 — Source data Fig. 8 [file 44318_2024_81_MOESM8_ESM.zip › Fig8B/Fig8B_xNup155-Nb1t_xNup98-Nb1t/Fig8B_xNup155-Nb1t_xNup98-Nb1t_xhNup155_Nb2i.tif]

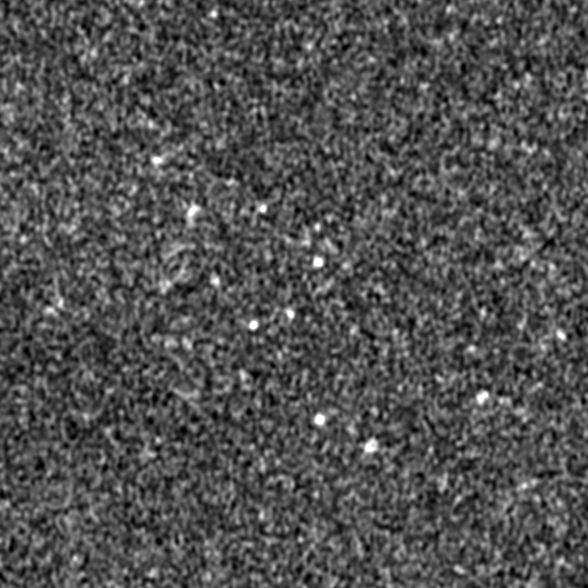

Supplement: Supplementary file 8 — Source data Fig. 8 [file 44318_2024_81_MOESM8_ESM.zip › Fig8B/Fig8B_xNup155-Nb1t_xNup98-Nb1t/Fig8B_xNup155-Nb1t_xNup98-Nb1t_xhNup155_Nb3i.tif]

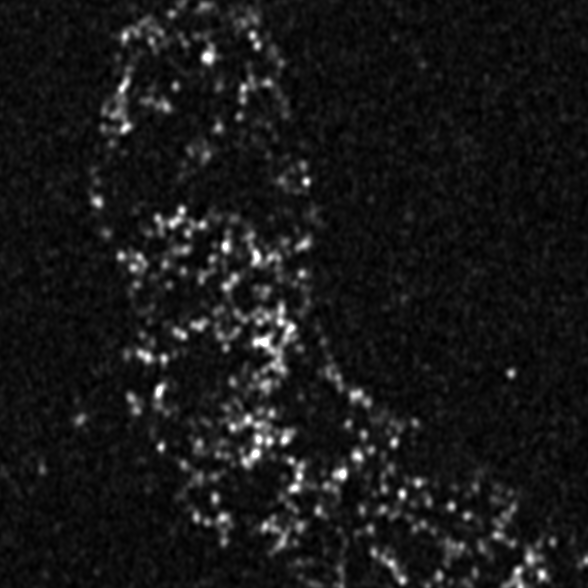

Supplement: Supplementary file 8 — Source data Fig. 8 [file 44318_2024_81_MOESM8_ESM.zip › Fig8B/Fig8B_xNup155-Nb1t_xNup98-Nb1t/Fig8B_xNup155-Nb1t_xNup98-Nb1t_xhNup93_Nb4i.tif]

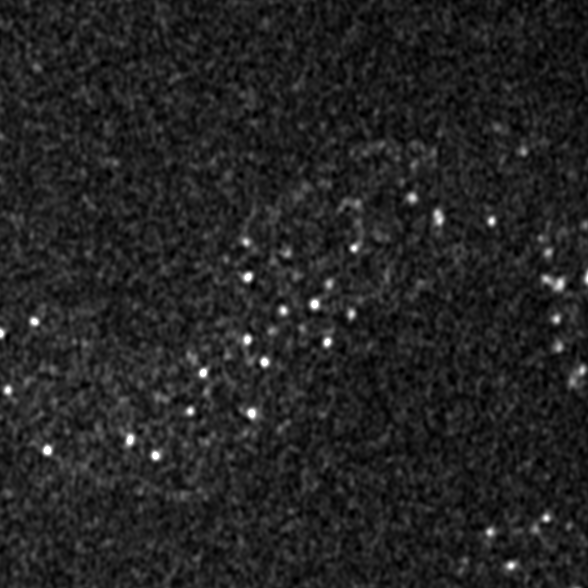

Supplement: Supplementary file 8 — Source data Fig. 8 [file 44318_2024_81_MOESM8_ESM.zip › Fig8B/Fig8B_xNup155-Nb1t_xNup98-Nb1t/Fig8B_xNup155-Nb1t_xNup98-Nb1t_xhNup98_Nb2i.tif]

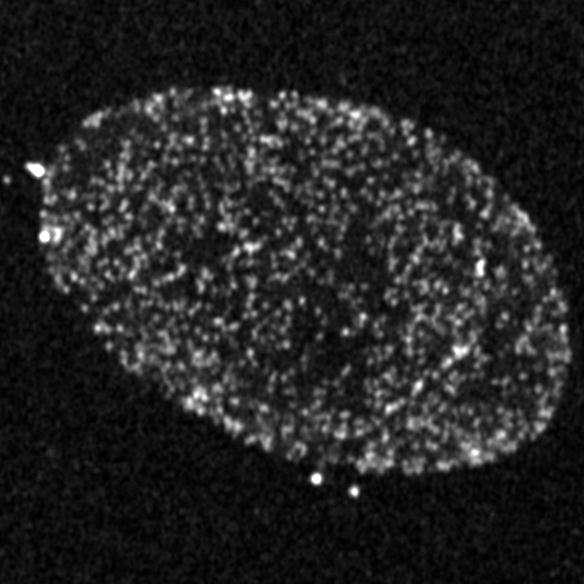

Supplement: Supplementary file 8 — Source data Fig. 8 [file 44318_2024_81_MOESM8_ESM.zip › Fig8B/Fig8B_xNup358-Nb1t/Fig8B_xNup358-Nb1t_Buffer.tif]

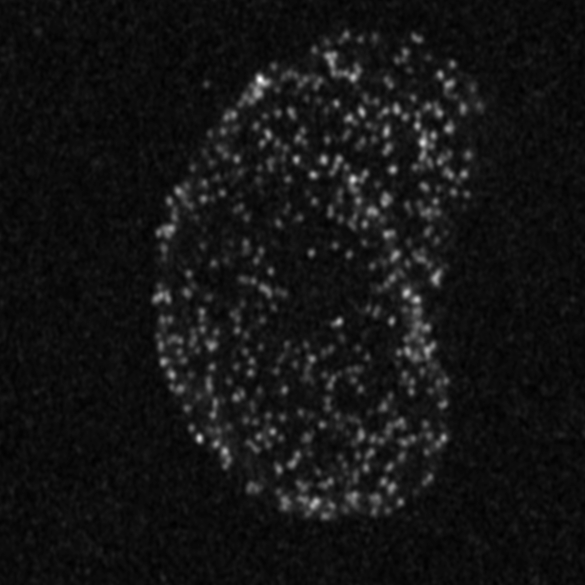

Supplement: Supplementary file 8 — Source data Fig. 8 [file 44318_2024_81_MOESM8_ESM.zip › Fig8B/Fig8B_xNup358-Nb1t/Fig8B_xNup358-Nb1t_NoninhibitoryNb.tif]

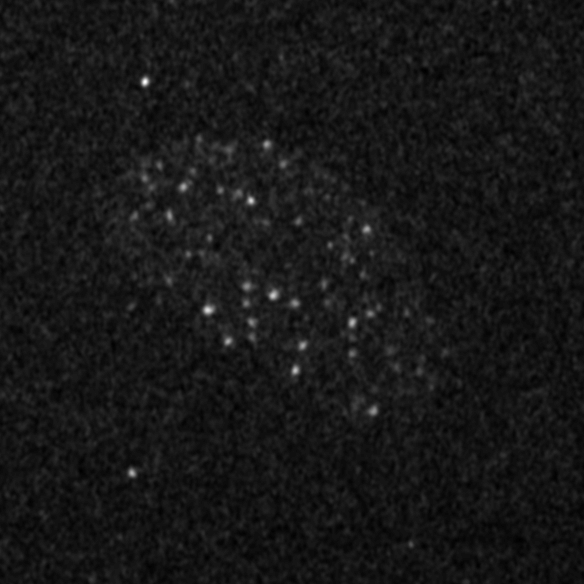

Supplement: Supplementary file 8 — Source data Fig. 8 [file 44318_2024_81_MOESM8_ESM.zip › Fig8B/Fig8B_xNup358-Nb1t/Fig8B_xNup358-Nb1t_xhNup155-Nb2i.tif]

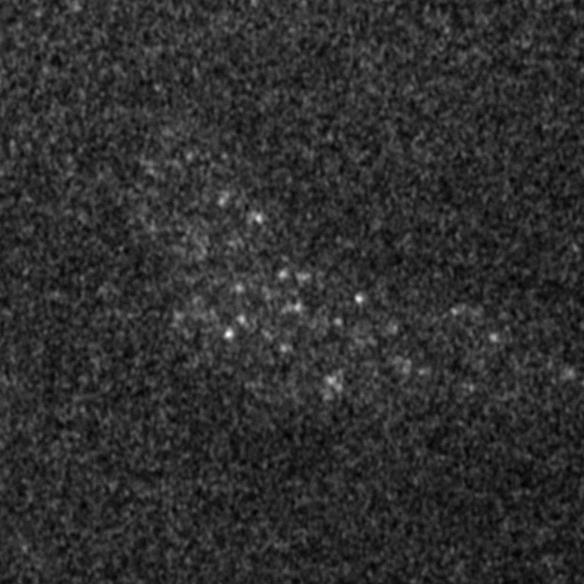

Supplement: Supplementary file 8 — Source data Fig. 8 [file 44318_2024_81_MOESM8_ESM.zip › Fig8B/Fig8B_xNup358-Nb1t/Fig8B_xNup358-Nb1t_xhNup155-Nb3i.tif]

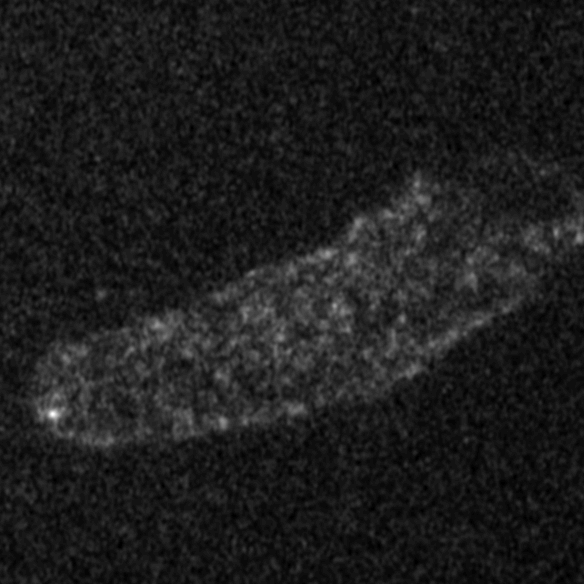

Supplement: Supplementary file 8 — Source data Fig. 8 [file 44318_2024_81_MOESM8_ESM.zip › Fig8B/Fig8B_xNup358-Nb1t/Fig8B_xNup358-Nb1t_xhNup93-Nb4i.tif]

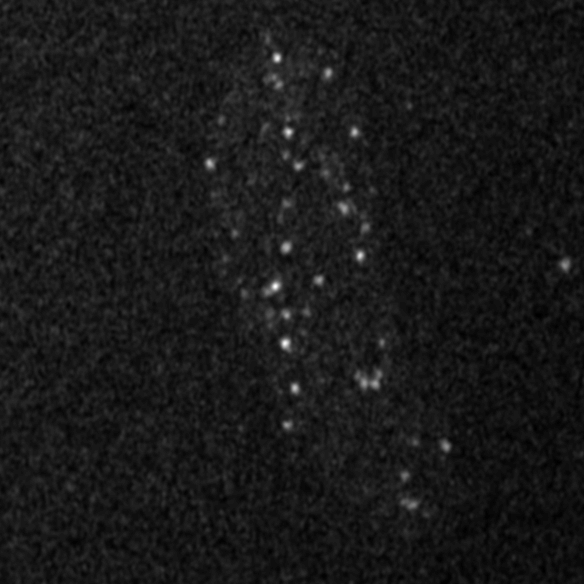

Supplement: Supplementary file 8 — Source data Fig. 8 [file 44318_2024_81_MOESM8_ESM.zip › Fig8B/Fig8B_xNup358-Nb1t/Fig8B_xNup358-Nb1t_xhNup98-Nb2i.tif]

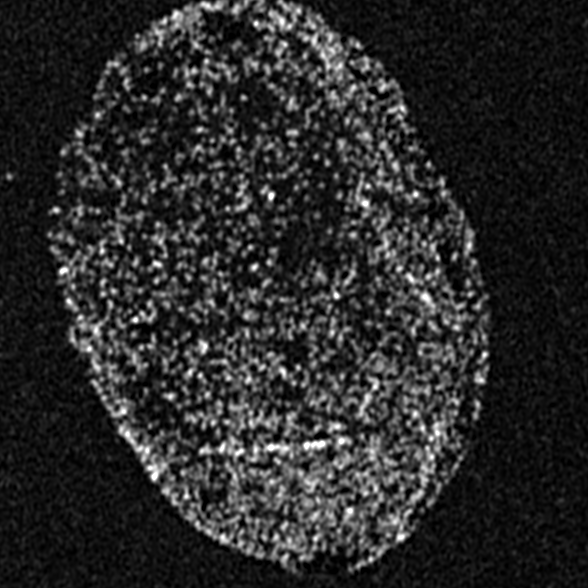

Supplement: Supplementary file 8 — Source data Fig. 8 [file 44318_2024_81_MOESM8_ESM.zip › Fig8B/Fig8B_xNup93-Nb1t_xhNup35-Nb1t/Fig8B_xNup93_Nb1t-xhNup35_Nb1t_Buffer_raw.tif]

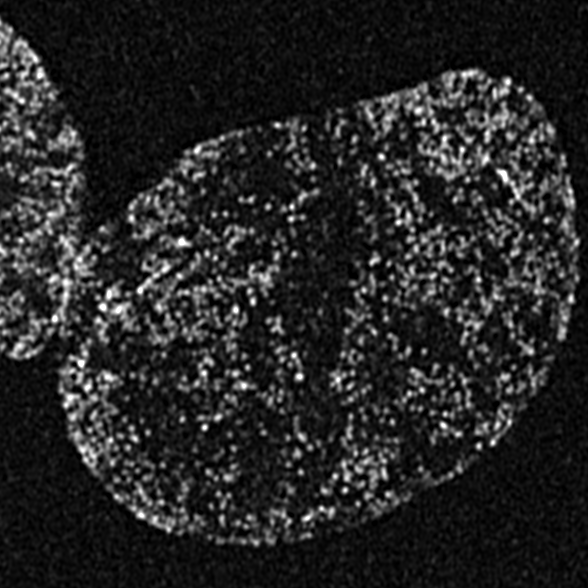

Supplement: Supplementary file 8 — Source data Fig. 8 [file 44318_2024_81_MOESM8_ESM.zip › Fig8B/Fig8B_xNup93-Nb1t_xhNup35-Nb1t/Fig8B_xNup93_Nb1t-xhNup35_Nb1t_NoninhibitoryNb_raw.tif]

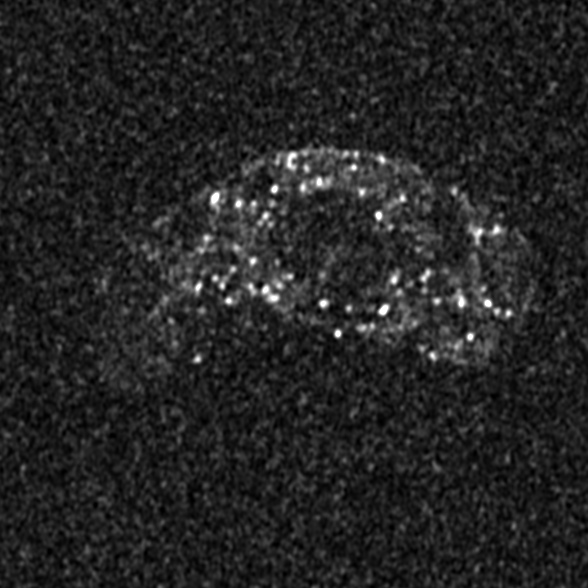

Supplement: Supplementary file 8 — Source data Fig. 8 [file 44318_2024_81_MOESM8_ESM.zip › Fig8B/Fig8B_xNup93-Nb1t_xhNup35-Nb1t/Fig8B_xNup93_Nb1t-xhNup35_Nb1t_xhNup155_Nb2i_raw.tif]

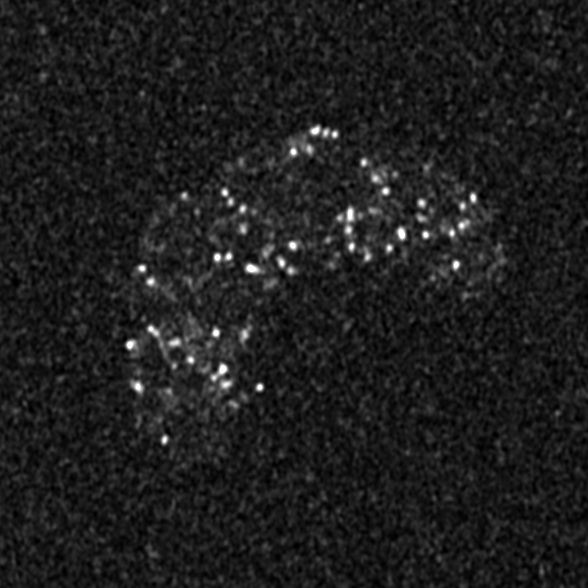

Supplement: Supplementary file 8 — Source data Fig. 8 [file 44318_2024_81_MOESM8_ESM.zip › Fig8B/Fig8B_xNup93-Nb1t_xhNup35-Nb1t/Fig8B_xNup93_Nb1t-xhNup35_Nb1t_xhNup155_Nb3i_raw.tif]

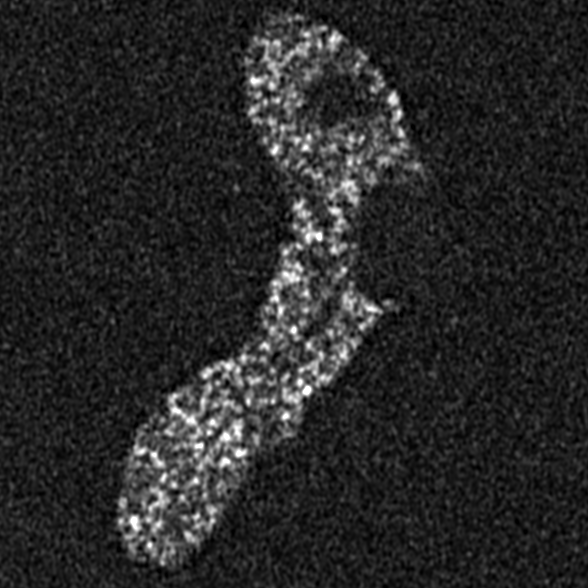

Supplement: Supplementary file 8 — Source data Fig. 8 [file 44318_2024_81_MOESM8_ESM.zip › Fig8B/Fig8B_xNup93-Nb1t_xhNup35-Nb1t/Fig8B_xNup93_Nb1t-xhNup35_Nb1t_xhNup93_Nb4i_raw.tif]

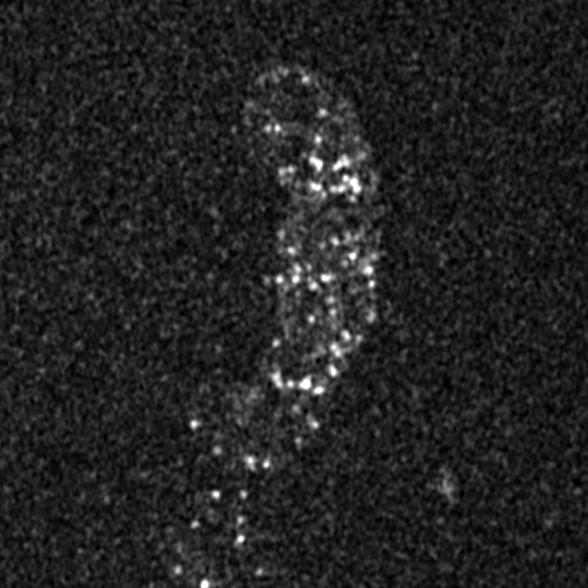

Supplement: Supplementary file 8 — Source data Fig. 8 [file 44318_2024_81_MOESM8_ESM.zip › Fig8B/Fig8B_xNup93-Nb1t_xhNup35-Nb1t/Fig8B_xNup93_Nb1t-xhNup35_Nb1t_xhNup98_Nb2i_raw.tif]

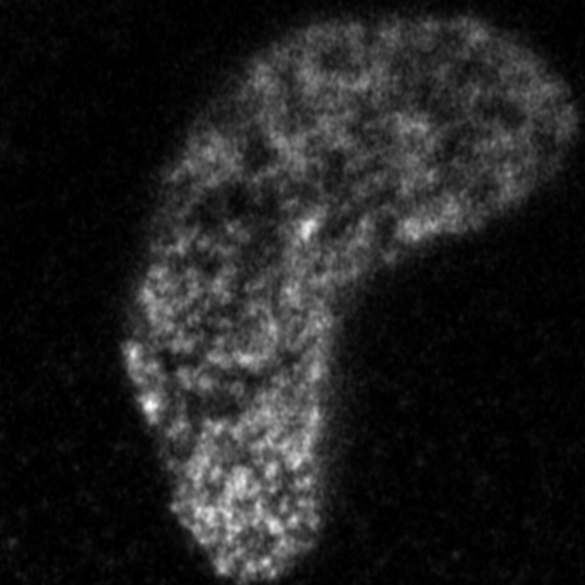

Supplement: Supplementary file 8 — Source data Fig. 8 [file 44318_2024_81_MOESM8_ESM.zip › Fig8B/Fig8B_xY-Nb1t_xNup62cmplx-Nb1t_xhNup214-Nb1t/Fig8B_xYcmplx_Nb1t_xNup62cmplx_Nb1t-xhNup214cmplx_Nb1t_Buffe.tif]

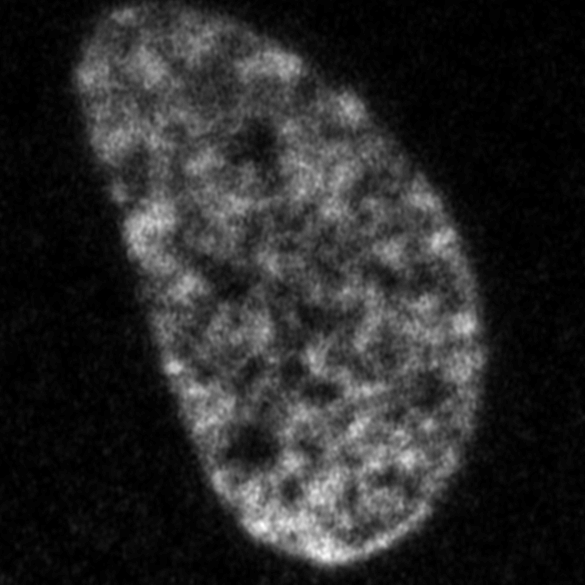

Supplement: Supplementary file 8 — Source data Fig. 8 [file 44318_2024_81_MOESM8_ESM.zip › Fig8B/Fig8B_xY-Nb1t_xNup62cmplx-Nb1t_xhNup214-Nb1t/Fig8B_xYcmplx_Nb1t_xNup62cmplx_Nb1t-xhNup214cmplx_Nb1t_NoninhibitoryNb.tif]

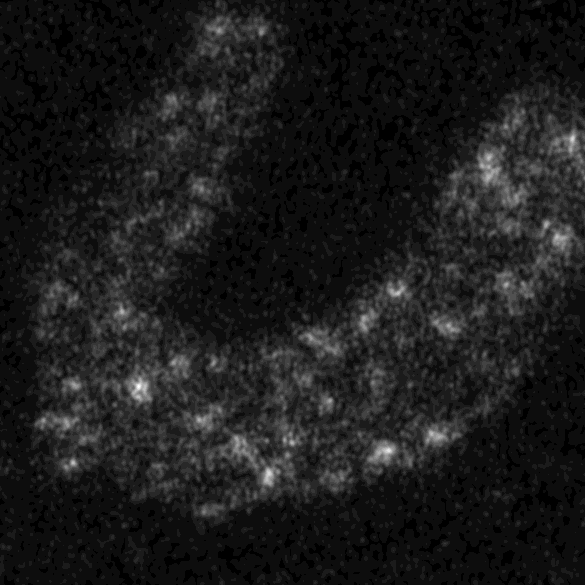

Supplement: Supplementary file 8 — Source data Fig. 8 [file 44318_2024_81_MOESM8_ESM.zip › Fig8B/Fig8B_xY-Nb1t_xNup62cmplx-Nb1t_xhNup214-Nb1t/Fig8B_xYcmplx_Nb1t_xNup62cmplx_Nb1t-xhNup214cmplx_Nb1t_xhNup155_Nb2i.tif]

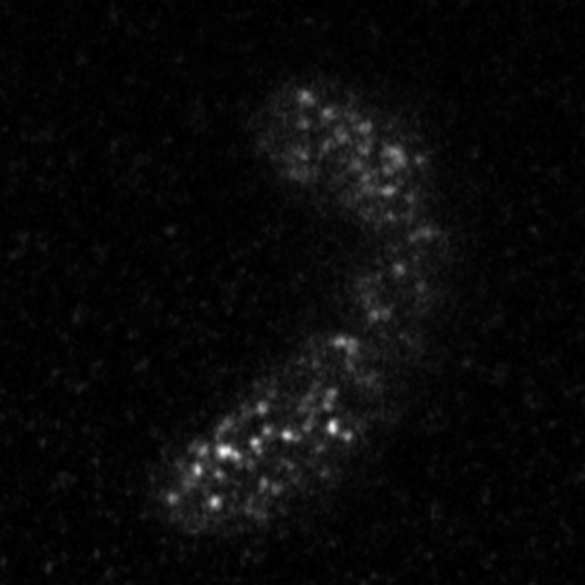

Supplement: Supplementary file 8 — Source data Fig. 8 [file 44318_2024_81_MOESM8_ESM.zip › Fig8B/Fig8B_xY-Nb1t_xNup62cmplx-Nb1t_xhNup214-Nb1t/Fig8B_xYcmplx_Nb1t_xNup62cmplx_Nb1t-xhNup214cmplx_Nb1t_xhNup155_Nb3i.tif]

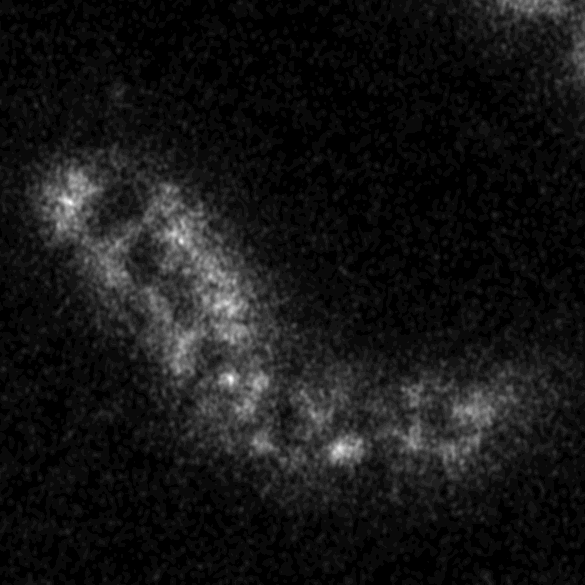

Supplement: Supplementary file 8 — Source data Fig. 8 [file 44318_2024_81_MOESM8_ESM.zip › Fig8B/Fig8B_xY-Nb1t_xNup62cmplx-Nb1t_xhNup214-Nb1t/Fig8B_xYcmplx_Nb1t_xNup62cmplx_Nb1t-xhNup214cmplx_Nb1t_xhNup93_Nb4i.tif]

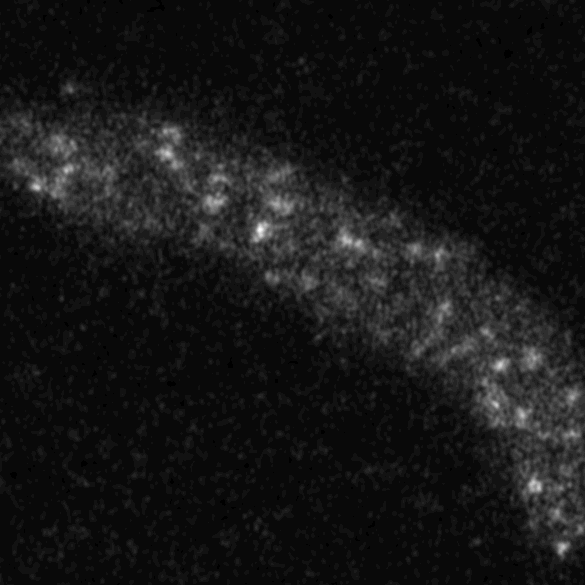

Supplement: Supplementary file 8 — Source data Fig. 8 [file 44318_2024_81_MOESM8_ESM.zip › Fig8B/Fig8B_xY-Nb1t_xNup62cmplx-Nb1t_xhNup214-Nb1t/Fig8B_xYcmplx_Nb1t_xNup62cmplx_Nb1t-xhNup214cmplx_Nb1t_xhNup98_Nb2i.tif]

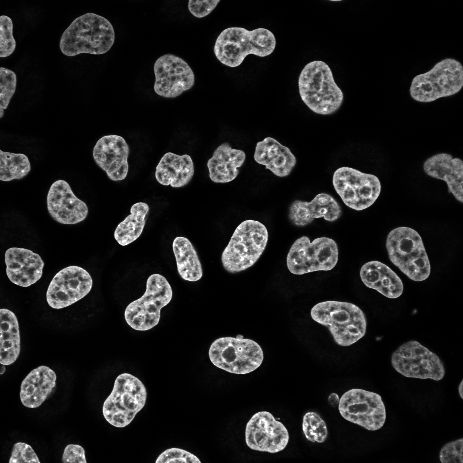

Supplement: Supplementary file 10 — Source data Fig. 10 [file 44318_2024_81_MOESM10_ESM.zip › Fig10A/Fig.10A-Mock.tif]

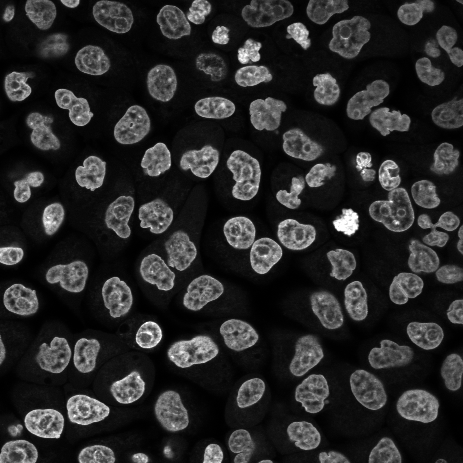

Supplement: Supplementary file 10 — Source data Fig. 10 [file 44318_2024_81_MOESM10_ESM.zip › Fig10A/Fig.10A-Nup358_siRNA.tif]

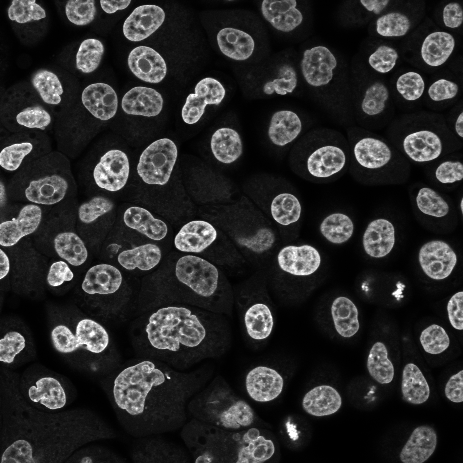

Supplement: Supplementary file 10 — Source data Fig. 10 [file 44318_2024_81_MOESM10_ESM.zip › Fig10A/Fig.10A-Nup358_siRNA_replicate.tif]

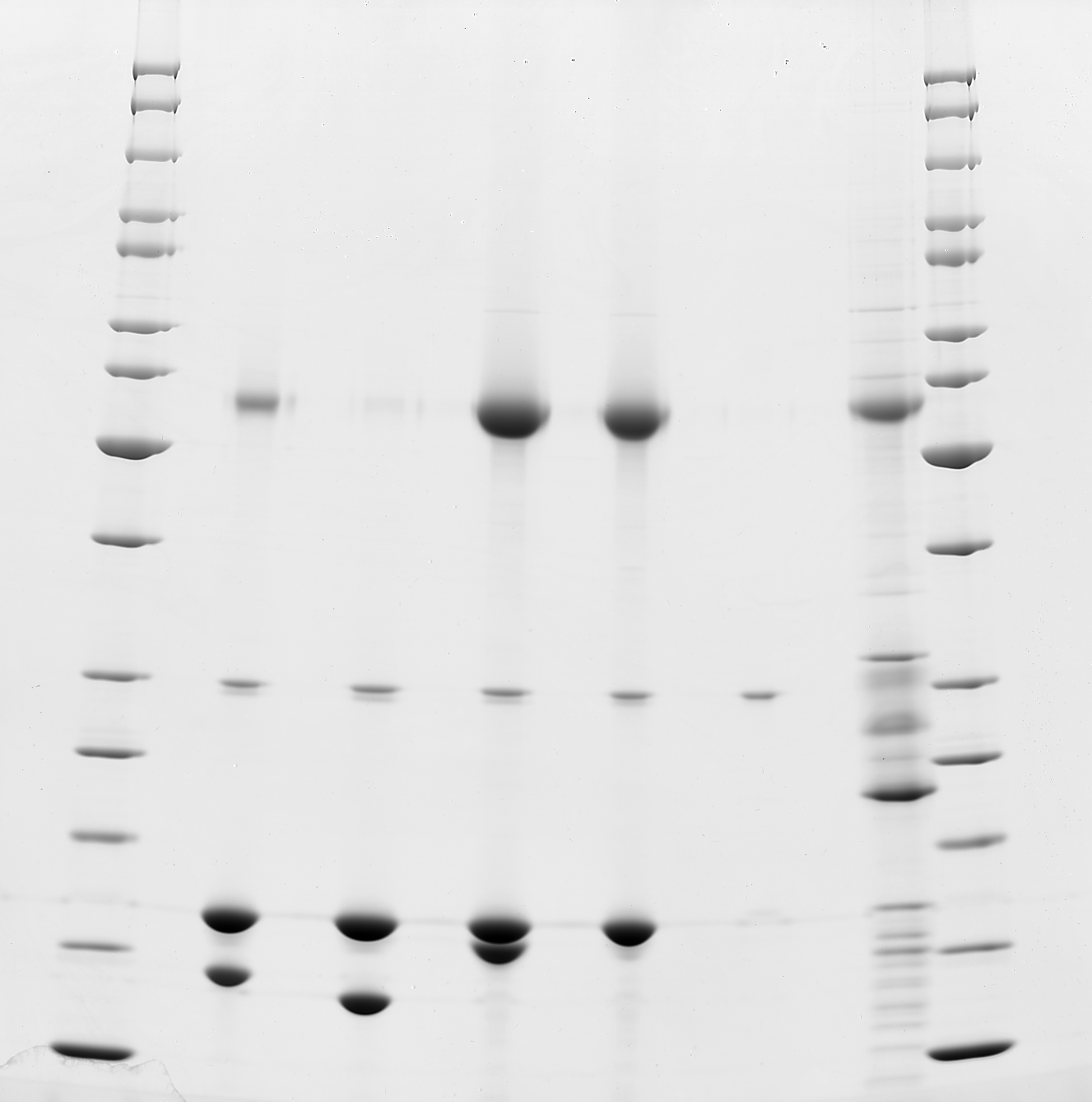

Supplement: Supplementary file 11 — Source data Fig. 11 [file 44318_2024_81_MOESM11_ESM.zip › Fig11C/Fig11C_Nup98_Nup88_gel.tif]

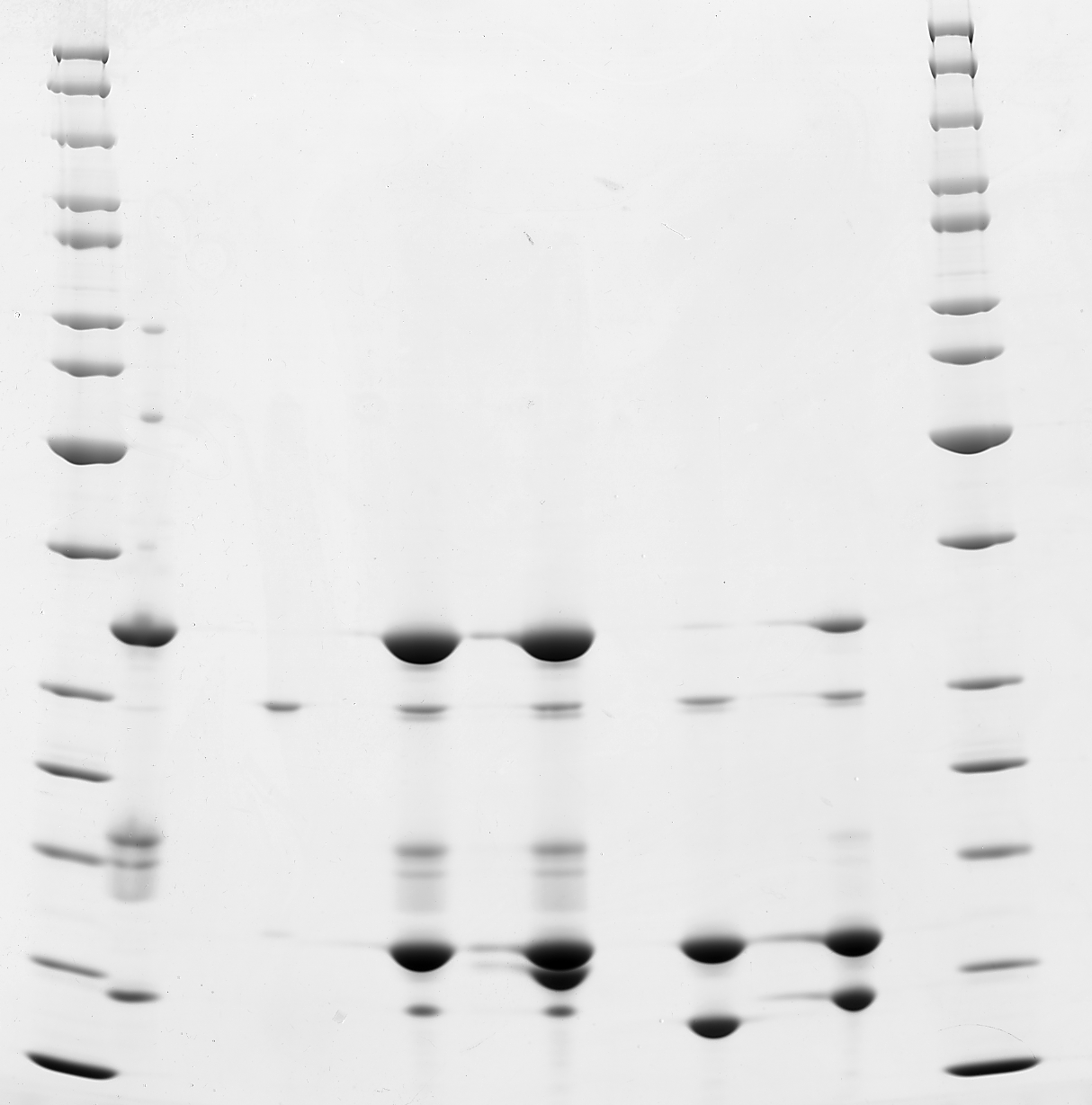

Supplement: Supplementary file 11 — Source data Fig. 11 [file 44318_2024_81_MOESM11_ESM.zip › Fig11C/Fig11C_Nup98_Nup96_gel.tif]

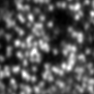

Supplement: Supplementary file 12 — Source data Fig. 12 [file 44318_2024_81_MOESM12_ESM.zip › Fig12A/Fig12A_BAPTA.tif]

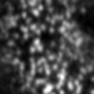

Supplement: Supplementary file 12 — Source data Fig. 12 [file 44318_2024_81_MOESM12_ESM.zip › Fig12A/Fig12A_Buffer.tif]

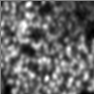

Supplement: Supplementary file 12 — Source data Fig. 12 [file 44318_2024_81_MOESM12_ESM.zip › Fig12A/Fig12A_Imp_beta.tif]

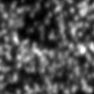

Supplement: Supplementary file 12 — Source data Fig. 12 [file 44318_2024_81_MOESM12_ESM.zip › Fig12A/Fig12A_WGA.tif]

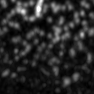

Supplement: Supplementary file 12 — Source data Fig. 12 [file 44318_2024_81_MOESM12_ESM.zip › Fig12B/Fig12B_Buffer.tif]

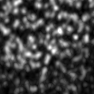

Supplement: Supplementary file 12 — Source data Fig. 12 [file 44318_2024_81_MOESM12_ESM.zip › Fig12B/Fig12B_ControlNb.tif]

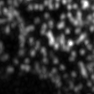

Supplement: Supplementary file 12 — Source data Fig. 12 [file 44318_2024_81_MOESM12_ESM.zip › Fig12B/Fig12B_xhNup98_Nb2i.tif]

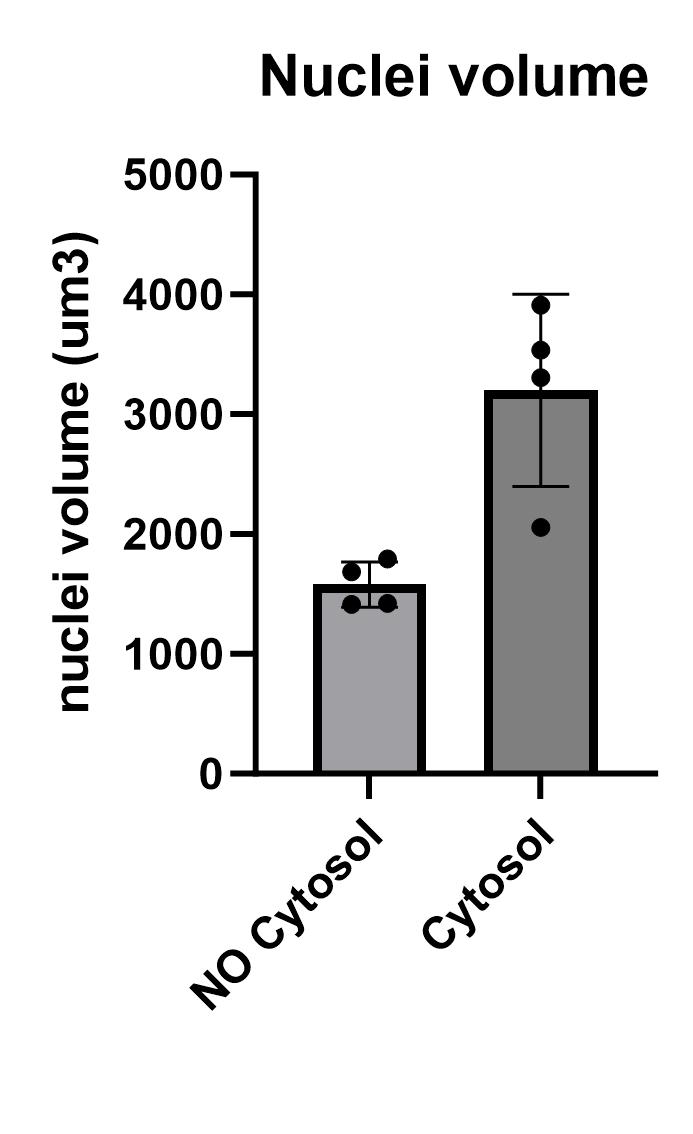

Supplement: Supplementary file 13 — EV Figure Source Data [file 44318_2024_81_MOESM13_ESM.zip › FigEV1B/Nuclei volume - Prism image.jpg]

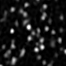

Supplement: Supplementary file 13 — EV Figure Source Data [file 44318_2024_81_MOESM13_ESM.zip › FigEV3/FigEV3-Ycomplex_Ndc1_Extract.tif]

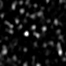

Supplement: Supplementary file 13 — EV Figure Source Data [file 44318_2024_81_MOESM13_ESM.zip › FigEV3/FigEV3-Ycomplex_Ndc1_Noextract.tif]

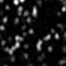

Supplement: Supplementary file 13 — EV Figure Source Data [file 44318_2024_81_MOESM13_ESM.zip › FigEV3/FigEV3-Ycomplex_gp210_Extract.tif]

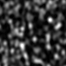

Supplement: Supplementary file 13 — EV Figure Source Data [file 44318_2024_81_MOESM13_ESM.zip › FigEV3/FigEV3-Ycomplex_gp210_Noextract.tif]

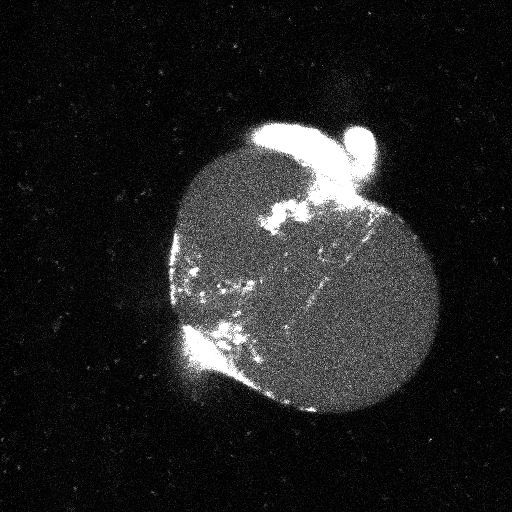

Supplement: Supplementary file 13 — EV Figure Source Data [file 44318_2024_81_MOESM13_ESM.zip › FigEV5B/Buffer.tif]

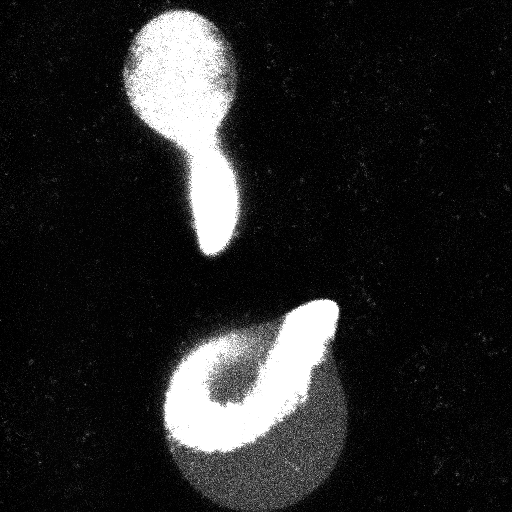

Supplement: Supplementary file 13 — EV Figure Source Data [file 44318_2024_81_MOESM13_ESM.zip › FigEV5B/Non-inhibitory_Nb_0mins.tif]

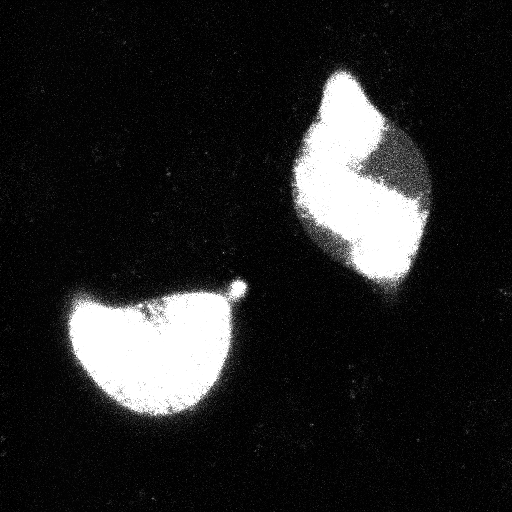

Supplement: Supplementary file 13 — EV Figure Source Data [file 44318_2024_81_MOESM13_ESM.zip › FigEV5B/Non-inhibitory_Nb_30mins.tif]

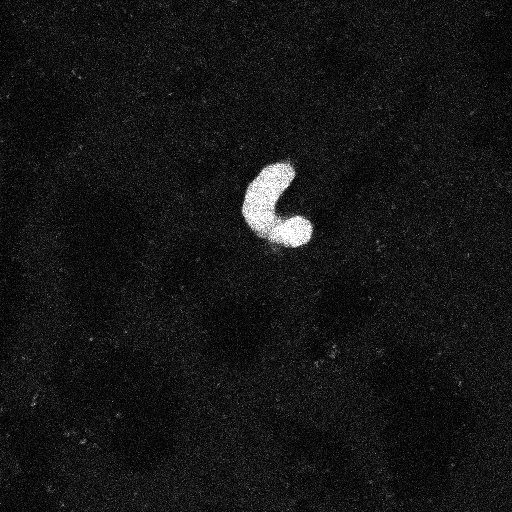

Supplement: Supplementary file 13 — EV Figure Source Data [file 44318_2024_81_MOESM13_ESM.zip › FigEV5B/xhNup155-Nb2i_0mins.tif]

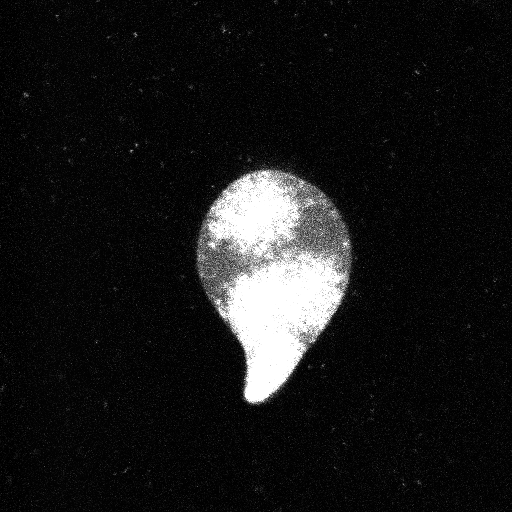

Supplement: Supplementary file 13 — EV Figure Source Data [file 44318_2024_81_MOESM13_ESM.zip › FigEV5B/xhNup155-Nb2i_30mins.tif]

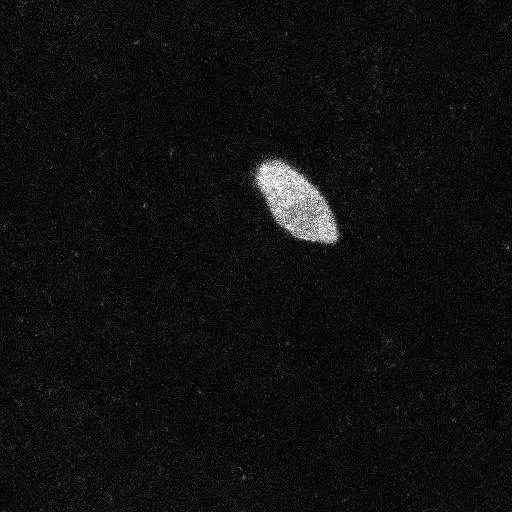

Supplement: Supplementary file 13 — EV Figure Source Data [file 44318_2024_81_MOESM13_ESM.zip › FigEV5B/xhNup155-Nb3i_0mins.tif]

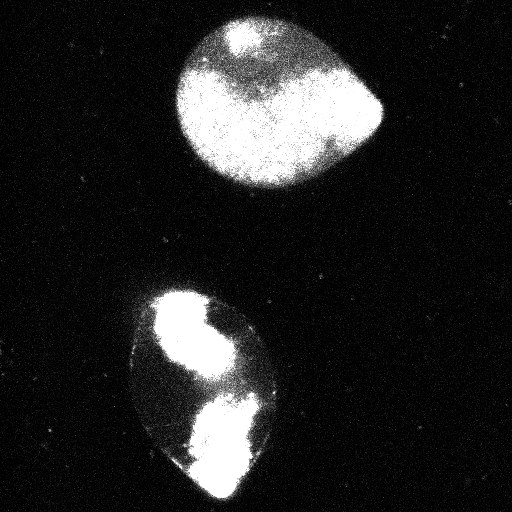

Supplement: Supplementary file 13 — EV Figure Source Data [file 44318_2024_81_MOESM13_ESM.zip › FigEV5B/xhNup155-Nb3i_30mins.tif]

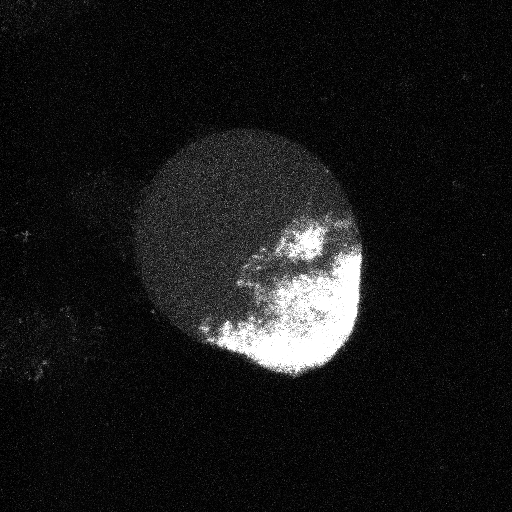

Supplement: Supplementary file 13 — EV Figure Source Data [file 44318_2024_81_MOESM13_ESM.zip › FigEV5B/xhNup93-Nb4i_0mins.tif]

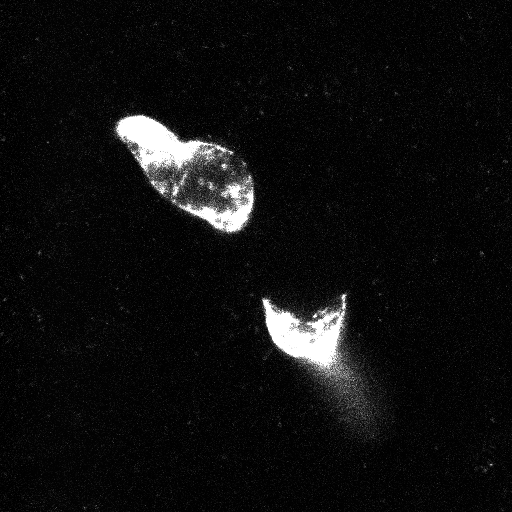

Supplement: Supplementary file 13 — EV Figure Source Data [file 44318_2024_81_MOESM13_ESM.zip › FigEV5B/xhNup93-Nb4i_30mins.tif]

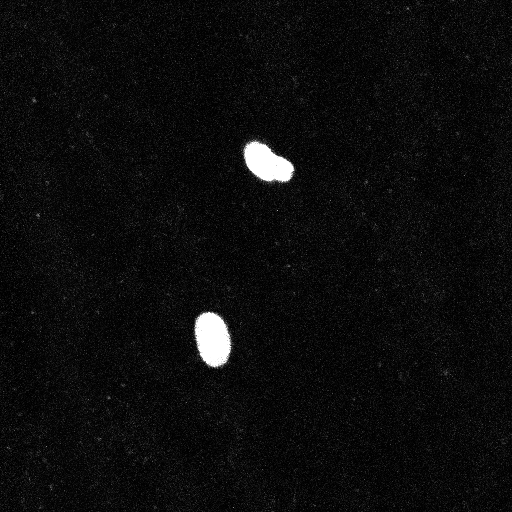

Supplement: Supplementary file 13 — EV Figure Source Data [file 44318_2024_81_MOESM13_ESM.zip › FigEV5B/xhNup98-Nb2i_0mins.tif]

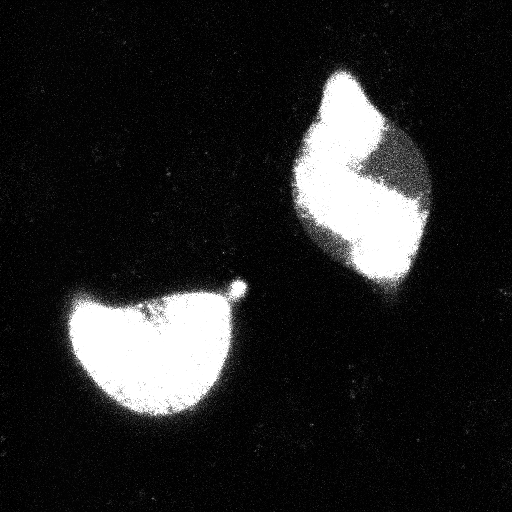

Supplement: Supplementary file 13 — EV Figure Source Data [file 44318_2024_81_MOESM13_ESM.zip › FigEV5B/xhNup98-Nb2i_30mins.tif]
