# Supplementary material for: A checkpoint function for Nup98 in nuclear pore formation suggested by novel inhibitory nanobodies
Source: EMBO J. 2024 Apr 22;43(11):6. doi: 10.1038/s44318-024-00081-w (PMC11148069; doi:10.1038/s44318-024-00081-w)
Supplement: Supplementary file 15 — Expanded View Figures [file 44318_2024_81_MOESM15_ESM.pdf]

Expanded View Figures

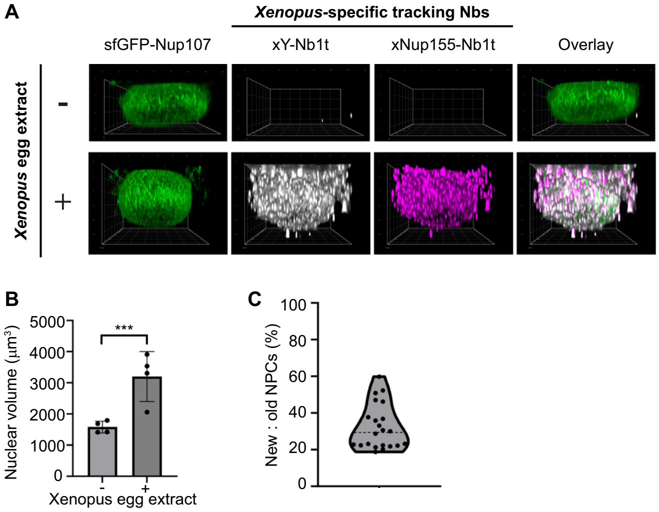

Figure EV1. HeLa cell nuclei grow in volume in the presence of *Xenopus* egg extracts.

(**A**) Interphase insertion of *Xenopus* NPCs from egg extract into human nuclei was performed as in Fig. 3. 3D reconstructions of nuclei were obtained from the acquired 3-channel z-stacks using the Arivis Vision4D software (version 3.1.3; VisionVR, 2020). (**B**) Volumes of 4 individual nuclei per condition from two independent experiments were integrated in the GFP channel using FiJi and plotted. Incubation with egg extract increased the average nuclear volume from 1500 to 3000 µm³. *** significant difference with a *P* value of 0.008 (unpaired *t* test). (**C**) Quantification of new NPC insertion during interphase assembly. NPCs were detected and quantified using a FiJi script. Numbers are normalized to the number of pre-existing human NPCs (= 100%). Each dot represents one quantified nucleus. Source data are available online for this figure.

© The Author(s)

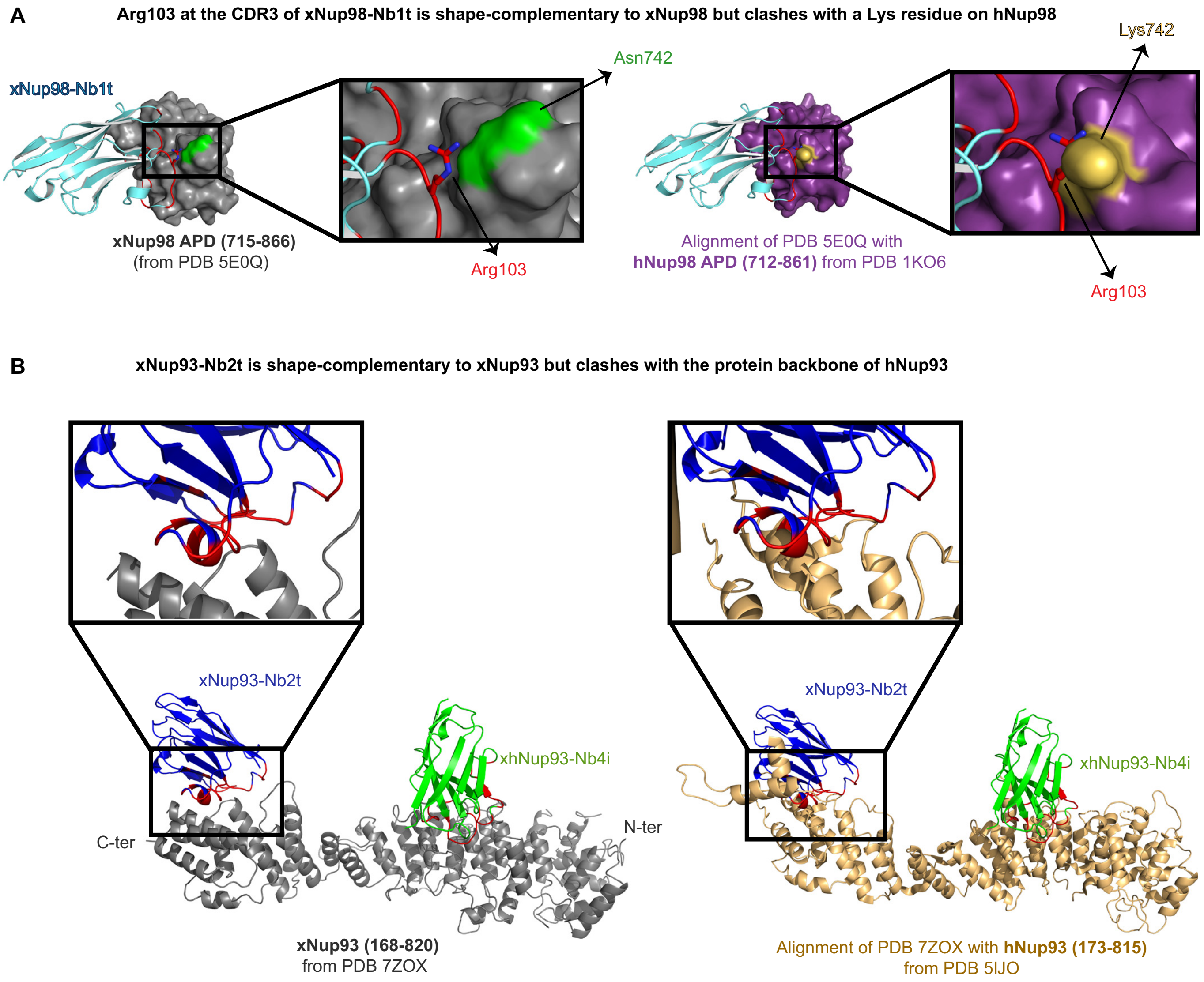

Figure EV2. *Xenopus*-specific anti-Nup nanobodies are incompatible with the corresponding human Nup targets.

(**A**) The crystal structure of the xNup98-xNup98-Nb1t complex shows that an arginine residue at the nanobody's CDR3 interacts with an asparagine residue at the APD of *Xenopus* Nup98 (left) (PDB 5E0Q; Pleiner et al, 2015). However, this arginine would clash with a lysine at the same position in hNup98, preventing its binding (right). (**B**) xNup93-Nb2t fits well to a pocket at the C-terminus of *Xenopus* Nup93 (PDB 7ZOX, Fig. 10), but would clash with the backbone of human Nup93 at the same protein region.

 © The Author(s)

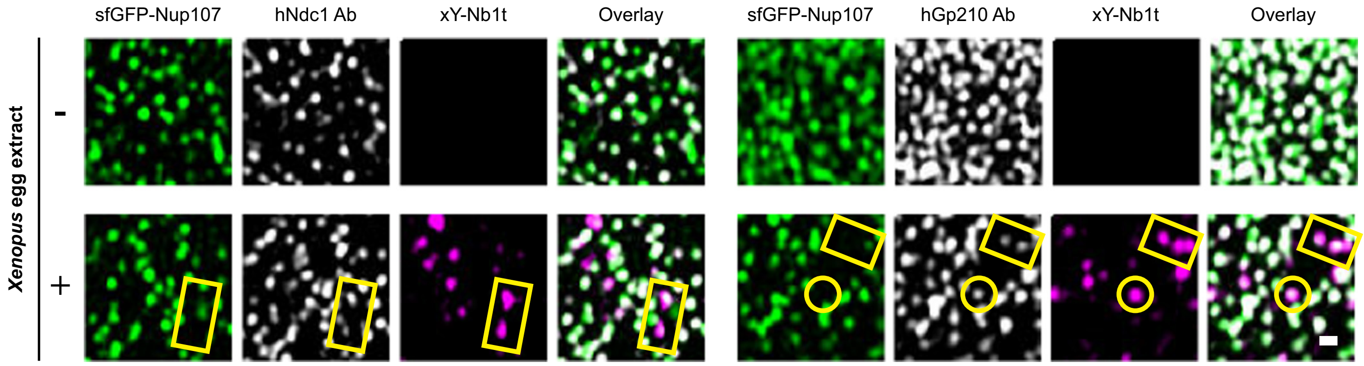

Figure EV3. Human membrane Nups get assembled into *Xenopus* NPCs.

Cells were stained with *Xenopus*-specific x-Ycmplx-Nb1t coupled to Alexa Fluor 647 and antibodies against human Ndc1 and human Gp210 (Stavru et al, 2006a, 2006b). Images were acquired as in as in Fig. 4. Circles or rectangles mark newly inserted pores. Scale bar, 0.25 μm. Source data are available online for this figure.

© The Author(s)

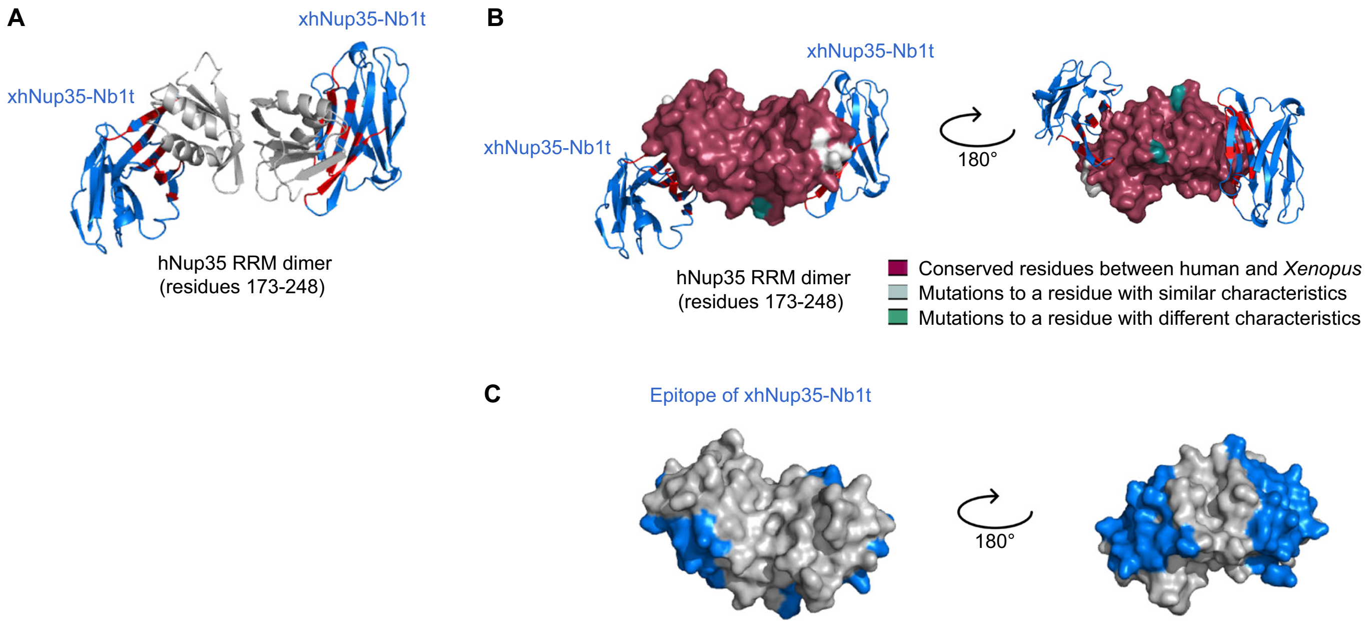

Figure EV4. xhNup35-Nb1t recognizes a conserved epitope at the RRM domain of Nup35.

(**A**) Crystal structure of the homodimeric RRM domain of *Homo sapiens* (h)Nup35 (gray) in complex with xhNup35-Nb1t (blue). The nanobody paratope is highlighted in red. See Appendix Table S1 for crystallographic statistics. (**B**) Surface representations of the Nup35 RRM domain color-coded according to amino acid conservation between human and *Xenopus* Nup35. The RRM domain and the nanobody epitope are highly conserved, as also indicated by the nanobody's cross-reaction between human and *Xenopus* Nup35 (see Figs. 4 and 6A, B). (**C**) The xhNup35-Nb1t epitope is highlighted in blue to allow for a straightforward comparison with (**B**). Although the Nup35 RRM domain mediates Nup35 homodimerization, which is necessary for the assembly of functional NPCs (Vollmer et al, 2012), xhNup35-Nb1t recognizes an epitope distant from the dimerization interface and is compatible with the Nup35 dimerization, explaining why it does not interfere with the formation of functional NPCs. In addition, the Nup35 dimer associates with membranes directly or through Ndc1 at very early assembly steps (Vollmer et al, 2012; Eisenhardt et al, 2014), and it links Nup93 to the β-propeller of Nup155, which is essential for the organization of the NPC inner ring and the assembly of the NPC scaffold (Hawryluk-Gara et al, 2008; De Magistris et al, 2018). However, these critical interactions occur through short linear motifs located at the Nup35 disordered termini (Vollmer et al, 2012; Eisenhardt et al, 2014; Mosalaganti et al, 2022), again explaining why xhNup35-Nb1t does not impede NPC assembly. Indeed, the lack of a phenotype is consistent with homodimerization being the only essential function of this RRM domain.

 © The Author(s)

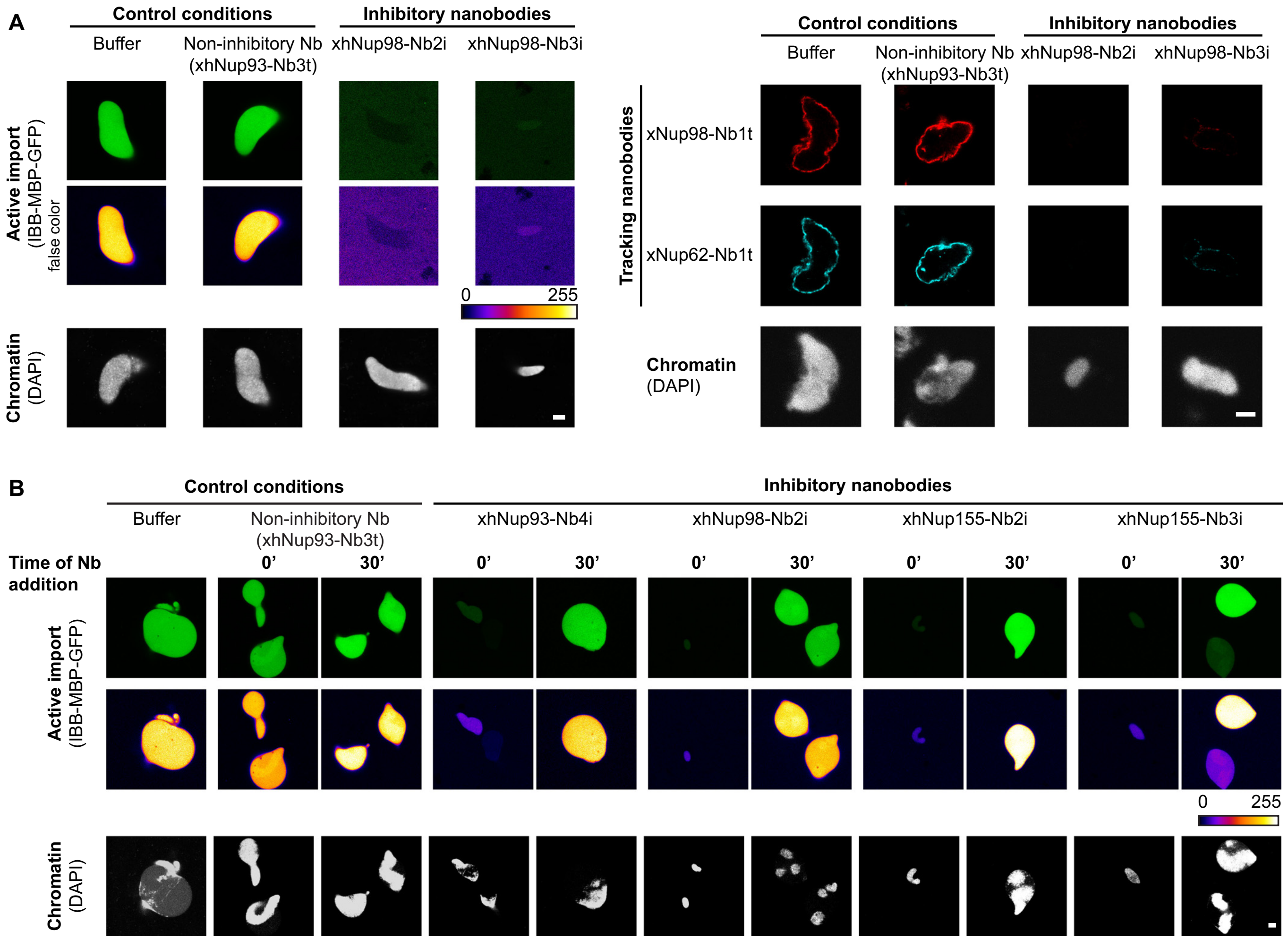

Figure EV5. Inhibitory anti-Nup nanobodies disrupt NPC assembly but do not block nuclear transport directly.

(**A**) 2 μM nanobodies were added to postmitotic NPC assembly reactions (as in Figs. 7 and 8). xhNup93-Nb3t had no deleterious effect. The presence of xhNup98-Nb2i and xhNup98-Nb3i, however, resulted in pseudonuclei that failed in active nuclear import of the IBB-MBP-GFP fusion (left) and in Nup recruitment to the NE (right). The coherent phenotype of the two inhibitory anti-Nup98 nanobodies is a stringent specificity control to rule out off-target effects as a cause, since the two belong to different classes and recognize different, though overlapping, epitopes (Fig. 11). They probably act by preventing the interaction of Nup98 with the Y-complex component Nup96 and with Nup88 (Fig. 11). Scale bar, 5 μm. (**B**) Nuclei were assembled as in Figs. 7 and 8, and inhibitory nanobodies were added either prior to chromatin addition and assembly initiation (0′) or thereafter (30 min after membrane addition). Next, IBB-MBP-GFP and DAPI were added, and nuclei were imaged 90 min later. Note that the late addition of inhibitory nanobodies allowed for very efficient active import, ruling out that the nanobodies interfere directly with import through functional NPCs. Scale bar, 5 μm. Source data are available online for this figure.

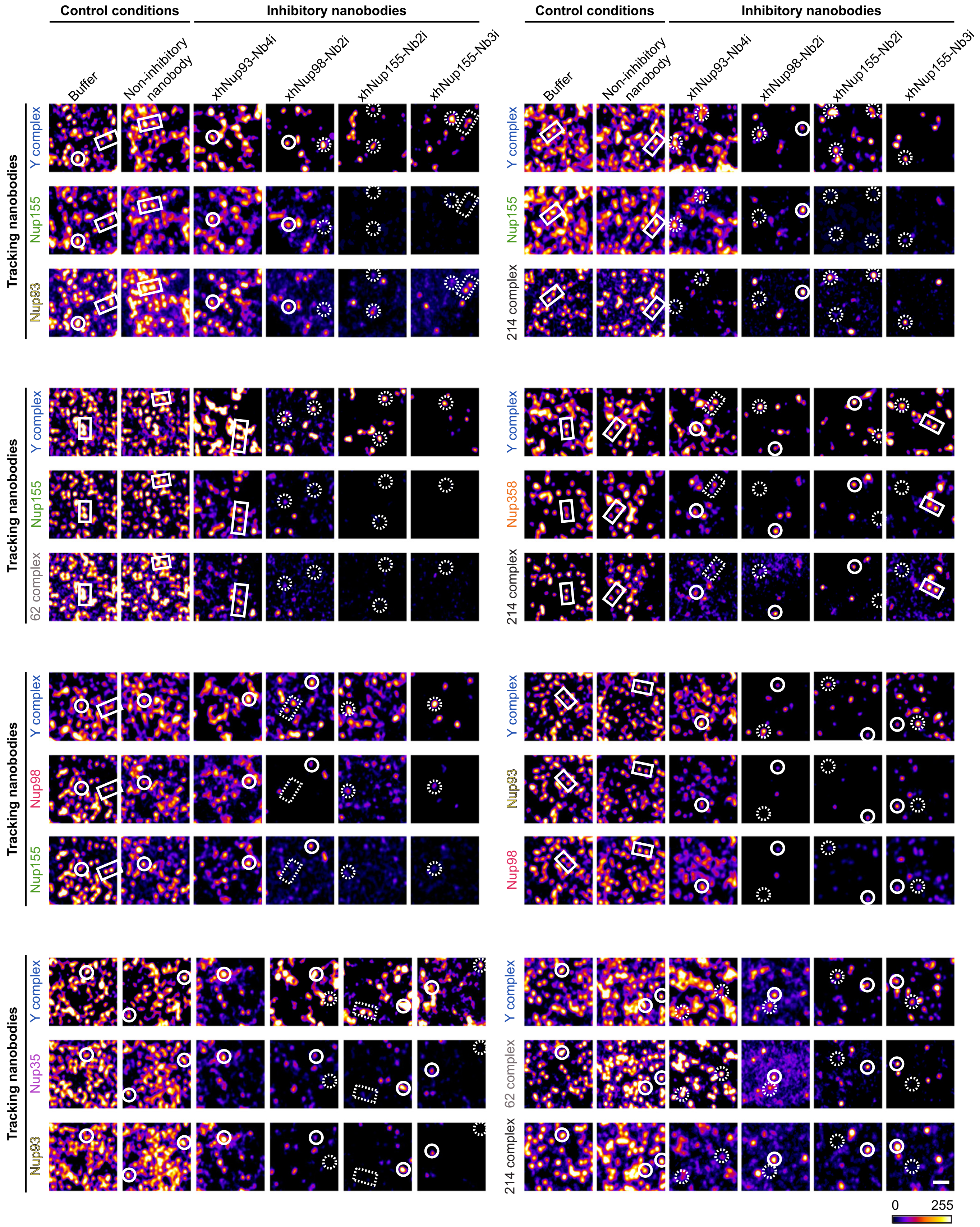
Control conditions
Inhibitory nanobodies
Buffer
Non-inhibitory nanobody
xhNup93-Nb4i
xhNup98-Nb2i
xhNup155-Nb2i
xhNup155-Nb3i
Tracking nanobodies
Y complex
Nup155
Nup93
214 complex
62 complex
Nup358
Nup98
Nup35
0
255

 © The Author(s)

Figure EV6. The assembly-inhibited 'NPCs' are decreased in number and altered in composition.

Postmitotic nuclear assembly in the presence of inhibitory nanobodies was as in Figs. 7 and 8, but 3-channel close-up views with single NPC resolution are shown. The following tracking nanobodies were used for staining: xY-Nb1t, xNup155-Nb1t, xNup93-Nb1t, xhNup35-Nb1t, xNup98-Nb1t, xNup62-Nb1t, xhNup214-Nb1t or xNup358-Nb1t. The non-inhibitory nanobodies used as controls were xhNup93-Nb3t, xNup93-Nb1t, xhNup35-Nb1t, xNup155-Nb1t, xNup358-Nb1t or xNup62-Nb1t. Pore-like structures, in which all three simultaneously probed Nup components were detected, are highlighted with white solid lines. Arrested structures with missing Nup components are highlighted with white dashed lines. A false-color representation (LUT) is shown to facilitate a comparison between the different images. Scale bar, 1 μm.

© The Author(s)
